# Global, regional, and national total burden related to hepatitis B and attributable risk factors in adults aged 65 years and older from 1990 to 2021 and projection to 2030

**DOI:** 10.3389/fpubh.2025.1654356

**Published:** 2025-09-25

**Authors:** Linying Gao, Yanfeng Ren, Songyue Hu, Yifan Zhang, Hongjing Bai, Jinbo Li, Keke Wang, Suping Wang, Yongliang Feng

**Affiliations:** 1 School of Public Health, Shanxi Medical University, Taiyuan, China; 2 Center of Clinical Epidemiology and Evidence Based Medicine, Shanxi Medical University, Taiyuan, China; 3 MOE Key Laboratory of Coal Environmental Pathogenicity and Prevention, Shanxi Medical University, Taiyuan, China; 4 Research Center for Reverse Etiology, Workstation of Academician, Shanxi Medical University, Taiyuan, China; 5 First Hospital of Shanxi Medical University, Taiyuan, China

**Keywords:** hepatitis B virus, global burden of disease, disability-adjusted life years, epidemiology, Joinpoint regression analysis, Bayesian age-period-cohort

## Abstract

**Background:**

Hepatitis B virus and its complications remain a major global public health threat. This study aimed to assess the global, regional, and national trends of total burden related to hepatitis B among older adults aged ≥65 years from 1990 to 2021 and predict trend to 2030.

**Methods:**

Drawing upon the Global Burden of Diseases (GBD) 2021, we obtained the data on incidence, prevalence, mortality, and disability-adjusted life years (DALYs) from 1990 to 2021, categorized by sex, age, and socio-demographic index (SDI). Associations of. these metrics and SDI were analyzed. Secular trends were evaluated employing average annual percentage changes (AAPC) and joinpoint regression, with future trajectories estimated through 2030 using the Bayesian age-period-cohort (BAPC) model.

**Results:**

From 1990 to 2021, the global age-standardized incidence rate (ASIR) of total burden related to hepatitis B among older adults aged ≥65 years declined from 710.53 to 591.79 per 100,000 population (AAPC −0.57%), and age-standardized DALYs rate (ASDR) declined from 927.65 to 605.93 per 100,000 population (AAPC -1.39%). ASIR and ASPR both showed significant decreasing trends in males and females, with persistently higher rates observed among males across all study years. The ASIR increased only in the >95 years age group, while a significant increase in DALYs was observed across all age strata, with the magnitude of increase escalating with advancing age. ASIR increased exclusively in high SDI countries (AAPC 0.16%). In 2021, Central Sub-Saharan Africa accounted for the peak ASIR (1,671.36 per 100,000 population), conversely North Africa and the Middle East showed the most pronounced ASDR decline (AAPC -2.74%). Both alcohol use and smoking emerged as the most consequential modifiable risk factors for DALYs in adults aged ≥65 years. Projections indicate declining trends in ASIR, ASDR, and ASMR for this population by 2030, whereas ASPR is projected to exhibit an upward trend.

**Conclusion:**

The global burden and trends of total burden related to hepatitis B among older adults aged ≥65 years showed a significant decline, with projections indicating continued reduction by 2030. However, notable regional and national disparities persist. Key challenges in managing health risks for adults aged ≥65 years remain centered on alcohol use and smoking.

## Highlights

- First comprehensive analysis of total burden related to hepatitis B trends (1990–2030) in adults aged ≥65 years, addressing a critical gap in aging-related HBV epidemiology.
- Reveals persistent gender and socioeconomic disparities in total burden related to hepatitis B, highlighting males and low SDI regions as priority targets for tailored interventions.
- Identifies diverging risk factor trends across SDI strata, informing region-specific prevention strategies.
- Integrates BAPC model to project future disease trajectories, aiding policymakers in long-term HBV elimination planning amid global aging.

## Introduction

Hepatitis B virus (HBV) represents a notable global public health burden that can cause both acute and chronic infections, damaging the liver and leading to severe long-term consequences. It represents the predominant driver of cirrhosis, hepatocellular carcinoma (HCC), liver transplantation, and liver-related deaths worldwide (1, 2). According to World Health Organization (WHO) data, an estimated 254 million individuals had chronic HBV infection in 2022, with an annual incidence of 1.2 million new cases (3). Updated epidemiological records from 187 nations reveal a significant elevation in viral hepatitis mortality, with estimates climbing from 1.1 million fatalities in 2019 to 1.3 million in 2022 (4). In 2016, the WHO has set forth a global elimination target for viral hepatitis as a significant public health concern by 2030 (5). Despite therapeutic advances in potent antivirals and demonstrated efficacy of hepatitis B vaccination programs, HBV-related socioeconomic burdens remain disproportionately high.

Older adults are at high risk for hepatitis B infection, and infected older adults often present with subclinical hepatitis and have a lower HBV clearance rate (6). Furthermore, the progression of chronic hepatitis B is influenced by multiple factors, including age. Studies indicate that older adults exhibit a higher likelihood of developing chronic infection following initial HBV exposure (7), with subsequent progression to liver cirrhosis and hepatocellular carcinoma (8). A notable disease cluster in a Japanese geriatric care center revealed that nearly 60% of infected individuals became hepatitis B surface antigen (HBsAg) carriers (9), highlighting the distinct epidemiological patterns and disease outcomes in older age groups compared to younger populations. Since 1990, the prevalence among younger populations has decreased by 76.8%, while the HBsAg positivity rate remains significantly higher in older populations. Major risk factors for younger individuals include mother-to-child transmission, unsafe injections, healthcare-related procedures, and high-risk behaviors, whereas risk factors for older adults encompass historical exposure, prolonged infection duration, immunosenescence, comorbidities, and gaps in diagnosis and treatment (1).

The total burden related to hepatitis B permits epidemiological imputation to alcohol use, drug use, high body mass index, and smoking. Beyond advanced age, key determinants of HBV acquisition and progression require consideration, with tobacco use demonstrating significant attributable risk for HBV-related hepatocarcinogenesis (9). Alcohol consumption significantly elevates the risks of both cirrhosis and hepatocellular carcinoma. The predominant health burden of substance abuse stems from bloodborne pathogens transmitted via unsafe injection behaviors (7), and drug use significantly contributes to the elevated hepatitis B prevalence rates observed globally (10). Additionally, the total burden related to hepatitis B demonstrated a significant upward trend in BMI-related attributable risk. Strategic prevention of modifiable risks demonstrates significant potential for alleviating the total burden related to hepatitis B (11).

To date, there remains a paucity of research specifically addressing the global burden and long-term trends of hepatitis B infection among older adults aged ≥65 years. Furthermore, disparities in the total burden related to hepatitis B across genders and countries with varying socioeconomic development levels have been underexplored. Leveraging estimates from the Global Burden of Diseases, Injuries, and Risk Factors Study (GBD) 2021, this study analyzed the incidence, prevalence, mortality, and DALYs for total burden related to hepatitis B to quantify the disease burden among older adults aged ≥65 years from 1990 to 2021 at global, regional, and national levels. Trends were stratified by sex, age, and socio-demographic index (SDI), with additional exploration of the associations between SDI and the total burden related to hepatitis B. Projections of disease burden trends were extended to 2030. The findings not only expand the evidence base for understanding the total burden related to hepatitis B, but also provide critical insights to support global and national efforts toward achieving the 2030 viral hepatitis elimination goal as per WHO global health strategy.

## Methods

### Study population and data collection

Developed by the Institute for Health Metrics and Evaluation (IHME) at the University of Washington, the GBD study systematically quantifies global health trajectories and population health metrics. The study delivers continuously refined metrics quantifying health loss from hundreds of diseases, injuries, and risk factors from 1990 to 2021. The dataset encompasses 204 countries and territories, stratified into quintile SDI tiers (low, low-middle, middle, high-middle, high), with parallel classification into 21 geographically-defined GBD regions. Detailed methodologies for GBD data collection and estimation, including statistical modeling and uncertainty analysis, have been extensively described in prior publication (12). For this study, total burden related to hepatitis B data for older adults aged ≥65 years were extracted, the target demographic was categorized into seven subgroups: 65–69, 70–74, 75–79, 80–84, 85–89, 90–94, and ≥95 years. Data on location, age, and sex-specific incidence, prevalence, mortality, DALYs, and risk factor-attributable DALYs and mortality were systematically compiled for subsequent analysis. The 95% uncertainty interval (UI) for each metric was derived from the 2.5th and 97.5th percentiles range of 1,000 posterior draws generated through Markov chain Monte Carlo (MCMC) simulations (13).

The data sources include vital registration systems, verbal autopsies, censuses, household surveys, disease-specific registries, health service contact data, and other relevant records (14). GBD 2021 systematically adjusts epidemiological data using sophisticated statistical models such as MR-BRT and DisMod-MR 2.1 to eliminate biases arising from differences in data sources, definitions, and measurement methods. It reduces the impact of heterogeneity on results through standardization and calibration steps, ensuring the internal consistency of estimates across regions, ages, sexes, and years (15). Even for countries without primary data, the model can derive estimates by drawing on available data, thereby generating data on the total hepatitis B-related burden for all countries worldwide.

DisMod-MR 2.1 generates relevant metrics for locations lacking raw epidemiological data by estimating prevalence through a cascade down the five levels of the GBD geographical hierarchy. Epidemiological data from higher-level locations in the hierarchy serve as priors for estimating epidemiological parameters of lower-level locations. Meanwhile, it also uses location-level covariates to provide a basis for estimating the prevalence and incidence in locations with missing data. For some etiologies, spatiotemporal Gaussian process regression (ST-GPR) disease models are used instead of DisMod-MR 2.1. This is a set of regression methods applicable to analyzing heterogeneous and incomplete data, capable of performing statistical smoothing on data across time, age, and location dimensions.

### Definitions

DALYs serve as a well-established epidemiological measure for evaluating population health status, representing the combined burden of disease through years of life lost (YLLs) due to premature mortality and years lived with disability (YLDs) attributed to existing cases of a disease or health condition (16).

The SDI, a composite development metric scaled from 0 to 1, integrates three key indicators: per capita income, mean years of education for individuals aged ≥15 years and total fertility rate among females aged ≤25 years (17). This index reflects regional socioeconomic development and quantifies structural maturity in population demographics. Higher SDI values typically indicate advanced development levels and improved population health outcomes.

Alcohol use was defined as ≥10 g absolute alcohol daily among actively drinking individuals during the preceding year (18). Drug use refers to the regular consumption of substances such as opioids, cannabis, and cocaine (9). Smoking was defined as current or former consumption of any combustible tobacco products and other smoked tobacco formulations. The high BMI was defined as a BMI exceeding the 20–25 kg/m^2^ for adults aged ≥20 years (9).

### Risk factor

We computed and articulated the percentage of the total burden related to hepatitis B epidemiologically imputed to alcohol use, drug use, high BMI, and smoking. Estimation of GBD risk factors is based on a comparative risk assessment framework, initially, we determined the level of attribution of each risk factor, by conducting a comprehensive review of prior studies and incorporating pairs of risk and outcomes (19). Subsequently, we estimated the relative risk in relation to exposure and meta-regression assumptions, and computed the distribution of exposure for each risk factor by age, sex, location, and year using DisMod-MR 2.1 (20). Lastly, we established the theoretical minimum risk exposure level (TMREL) and assessed the population attributable fraction (PAF) and attributable burden. Detailed methodologies for risk factor estimation used in the GBD 2021 study are described in additional literature (14).

### Statistical analysis

The age-standardized incidence rate (ASIR), age-standardized prevalence rate (ASPR), age-standardized mortality rate (ASMR), and age-standardized DALYs rate (ASDR) (per 100,000 population, respectively) of total burden related to hepatitis B were computed employing the WHO-endorsed GBD 2021 reference population pyramid for direct standardization (17). The age-standardized rates (ASR) were computed by implementing the subsequent formula:


$$\mathrm{ASR}=\frac{\sum_{i=1}^{N}a_{i}w_{i}}{\sum_{i=1}^{N}w_{i}}$$

Within the mathematical formulation, $a_{i}$ denotes the age-specific rate in the ith age group, while $w_{i}$ represents the count of individuals within the same age group as per the GBD 2021 standard population. N is the total number of age groups.

Joinpoint regression model was employed to evaluate the temporal trends for the total burden related to hepatitis B, a method particularly advantageous for its ability to objectively identify inflection points where significant changes in trends occur within the time series, and calculate the average annual percentage change (AAPC). The model includeds both linear (y = xb) and log-linear (ln y = xb) specifications. Population-based trends in ASIR, ASPR, ASMR, and ASDR were assessed using Log-linear models. The optimal number (maximum of five) and positioning of joinpoints were optimally identified via a exhaustive grid search algorithm, with model selection optimized via Monte Carlo permutation testing. Linear regression was used to compute the slope for each trend segment to characterize upward or downward trajectories of total burden related to hepatitis B. Concurrently, the AAPC was calculated using the weighted average method to quantify the mean annual rate of change across the entire study period (21). Detailed computational procedures have been described in prior studies (22). The key analytical outputs of the joinpoint model encompassed AAPC, and their corresponding 95% confidence intervals (CIs). If the AAPC and its 95% CIs are greater than 0, the results demonstrate a statistically significant increasing trend; if they are less than 0, the results demonstrate a statistically significant decreasing trend. Including 0 suggests no statistically significant trend. The formula for AAPC is expressed as:


$$\text{AAPC}=\{\exp(\frac{\sum w_{i}b_{i}}{\sum w_{i}})-1\}\times 100$$

Within the mathematical formulation: $b_{i}$ is the slope coefficient for the ith segment with i indexing the segments in the desired range of years and $w_{i}$ is the length of each segment in the range of years.

This study employed the Bayesian age-period-cohort (BAPC) model to predict future disease burdens, as this approach demonstrates superior capability in addressing the complex data structures, high-dimensional parameters, and data sparsity issues commonly encountered in large-scale epidemiological analyses such as GBD 2021 (23). The model integrates age, period, and cohort effects within a Bayesian generalized linear framework. The model usessecond-order random walk to smooth temporal trends and applies the Integrated Nested Laplace Approximation (INLA) method for efficientestimation of posterior distributions. A distinguishing merit of the BAPC model lies in its innovative application of the INLA method to approximate marginal posterior distributions. In contrast to MCMC techniques that often suffer from mixing difficulties and convergence uncertainty, this approach maintains high computational efficiency. The model’s flexibility and robustness in analyzing time-series data significantly enhance its applicability for long-term disease burden forecasting (15). The BAPC model has undergone robust methodological validation and gained widespread application in epidemiological studies due to its ability to: provide comprehensive data coverage, accurately characterize temporal trends, andeffectively analyze age-stratified data while accounting for complex cohort effects. In this study, leveraging GBD 2021 data and IHME’s population projections, we constructed a predictive model using the “BAPC” R package to generate detailed projections of future global disease burden trends.

Additionally, the association between SDI and individual health outcome was examined through locally weighted scatterplot smoothing (LOWESS) regression analysis on the aggregated dataset spanning 1990 to 2021. All analyses were conducted using R software (version 4.3.3) and the Joinpoint Regression Program (version 5.0.2). A two-sided *p*-value <0.05 was considered statistically significant.

## Results

### Global trends

The global number of incident cases among older adults aged ≥65 years rose from 2.40 million in 1990 to 4.82 million in 2021, representing a 93.09% increase (Supplementary Table S1). However, the ASIR in 2021 was lower than in 1990 (591.79 vs. 710.53 per 100,000 population) (Supplementary Table S1). Additionally, the proportion of incident cases in this age group relative to total burden related to hepatitis B cases exhibited an overall upward trend, albeit with fluctuations between 2004 and 2006 (Supplementary Figure S1). Relative to the general age distribution of hepatitis B cases, the incidence rate among older adults aged ≥65 years demonstrated a marked decline from 1990 to 2021, though an upward trend was identified in high SDI countries (Supplementary Figure S2).

From 1990 to 2021, the number of prevalent cases, deaths, and DALYs for the total burden related to hepatitis B among older adults aged ≥65 years exhibited significant increases of 107.56, 57.80, and 51.39%, respectively (Supplementary Tables S1, S7), while the ASIR (AAPC −0.57%), ASPR (AAPC −0.35%), ASMR (AAPC −1.34%), and ASDR (AAPC -1.39%) demonstrated a sustained decline from 1990 to 2021 (Supplementary Table S2).

### Global trends by sex

**Figure 1:**
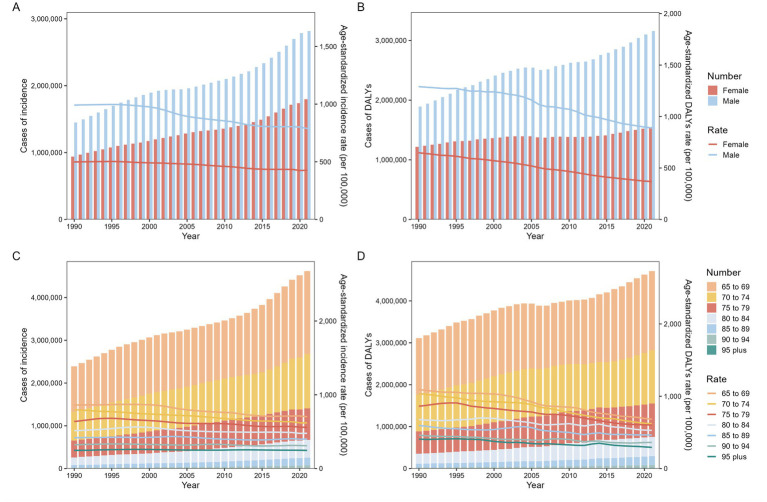
Incidence and DALYs of total burden related to hepatitis B in adults aged ≥65 years from 1990 to 2021 at the global level. **(A)** Cases and age-standardized incidence by sex; **(B)** Cases and age-standardized DALYs by sex; **(C)** Cases and age-standardized incidence by age; **(D)** Cases and age-standardized DALYs by age. DALYs disability-adjusted life-years.

From 1990 to 2021, the incident cases of the total burden related to hepatitis B among older adults aged ≥65 years increased for both males and females worldwide (males: from 1.45 million to 2.82 million, an increase of 94.33%; females: from 0.94 million to 1.80 million, an increase of 91.18%) (Supplementary Table S1). The ASIR in both sexes (AAPC −0.72% vs. −0.51%) exhibited declining trends during this period. Notably, the ASIR was persistently elevated in males compared to females throughout all age subgroups (Supplementary Table S2; Supplementary Figure S3; Figure 1). Comparative analysis demonstrated significantly higher ASPR in males compared to females in both 1990 and 2021 (1990: 4888.46 per 100,000 population vs. 3269.91 per 100,000 population; 2021: 4300.04 per 100,000 population vs. 2920.83 per 100,000 population). Similarly, the ASMR for males exceeded that for females in 2021 (49.61 per 100,000 population vs. 20.72 per 100,000 population) (Supplementary Table S7). Among older adults aged ≥65 years, the ASPR (AAPC −0.41% for males vs. −0.36% for females) and ASMR (AAPC -1.15% for males vs. -1.77% for females) demonstrated declining trends from 1990 to 2021, though the reduction was slower in males (Supplementary Table S2). Notably, males exhibited more pronounced reductions in AAPC relative to females throughout all older age groups, underscoring persistent gender disparities in disease burden trends (Supplementary Figure S6).

From 1990 to 2021, the ASDR of total burden related to hepatitis B among older adults aged ≥65 years showed minimal variation in decline, with males exhibiting a more modest reduction compared to females (AAPC -1.21% vs. -1.82%) (Supplementary Table S2). Within this age group, the ASDR for females was persistently and significantly lower than that for males in both 1990 (648.12 vs. 1290.72 per 100,000 population) and 2021 (368.70 vs. 889.58 per 100,000 population) (Supplementary Table S1). This gender disparity persisted stably across SDI strata, with males demonstrating persistently elevated burden metrics relative to females (Supplementary Figures S4, S5).

### Global trends by age subgroup

Globally, from 1990 to 2021, the incident cases of the total burden related to hepatitis B among older adults aged ≥65 years increased by at least half across all age groups. The ≥95 age group experienced the most significant growth, rising from 0.0025 million to 0.01 million (an increase of 432.56%) (Supplementary Table S1). However, the AAPC in total burden related to hepatitis B incidence demonstrated slight declines in most age groups, except for the ≥95 years subgroup, which showed a marginal increase (AAPC 0.01%). Significant declines were identified in the 65–69 years (AAPC −0.63%), 70–74 years (AAPC −0.83%), 75–79 years (AAPC −0.43%), 80–84 years (AAPC −0.22%), 85–89 years (AAPC −0.17%), and 90–94 years (AAPC −0.13%) subgroup (Supplementary Table S2).

The number of DALYs for the total burden related to hepatitis B increased significantly across all age groups from 1990 to 2021, with the magnitude of increase escalating with advancing age groups. The ≥95 years subgroup exhibited the largest rise in DALYs, surging from 4,000 to 16,000 cases (an increase of 284.19%). However, the ASDR for this subgroup (292.59 per 100,000 population) remained lower than other age groups (Supplementary Table S1). Overall, ASMR demonstrated substantial declines in all age groups, while the ASPR showed a marginal increase only in the ≥95 years subgroup (Supplementary Tables S1, S2; Supplementary Figure S7).

### Global trends by SDI

From 1990 to 2021, incident cases increased significantly throughout all SDI groups, demonstrating the most modest growth in the low SDI group (an increase of 92.38%) compared to the most pronounced expansion in the high SDI group (an increase of 103.11%). Among older adults aged ≥65 years, the ASIR of total burden related to hepatitis B increased only in high SDI countries (AAPC 0.16%), while declining trends were noted in other SDI categories, with the smallest reduction in low SDI countries (AAPC −0.50%) (Supplementary Table S2). Notably, the decline in ASIR among the ≥65-year population was consistently less pronounced compared to the general population across all SDI levels (Supplementary Figures S2; S8). During this period, the number of prevalent cases rose substantially in all SDI groups, exceeding 80% increases, with the middle SDI group experiencing the largest surge (an increase of 117.26%). Despite these rises, ASPR and ASMR declined in all SDI categories, with the most substantial declines observed in the middle SDI group (AAPC −0.94% for ASPR and −1.83% for ASMR) (Supplementary Table S2).

### Regional trends

From 1990 to 2021, Andean Latin America demonstrated the most pronounced increase in incident cases among older adults aged ≥65 years, rising from 6,700 to 20,000 cases (an increase of 205.23%). Among the 21 GBD regions, only High-income North America and Southern Latin America exhibited rising trends in ASIR for hepatitis B in this age group. In 2021, the ASIR in Central Sub-Saharan Africa (1,671.36 per 100,000 population) was approximately 16 times higher than that in High-income North America (104.78 per 100,000 population) (Supplementary Table S1). The most substantial decline in ASIR was observed in Tropical Latin America (AAPC -2.52%).

Notably, only Eastern Europe and High-income North America showed increasing trends in ASDR for total burden related to hepatitis B (AAPC 0.32 and 0.90%, respectively). Conversely, North Africa and the Middle East demonstrated the steepest decline in ASDR (AAPC -2.74%), while Australasia had the slowest reduction (AAPC −0.18%) (Supplementary Table S2; Supplementary Figure S9). In 2021, Eastern Sub-Saharan Africa reported the highest ASDR for hepatitis B among older adults aged ≥65 years (1,792.12 per 100,000 population) (Supplementary Table S1). Tropical Latin America and North Africa and the Middle East exhibited the most significant reductions in ASPR (AAPC -2.52%) and ASMR (AAPC -2.89%) rates, respectively. Central Sub-Saharan Africa exhibited the most elevated ASPR in 2021 (12,802.07 per 100,000 population), while the peak ASDR was documented in Eastern Sub-Saharan Africa (1,792.12 per 100,000 population) among older adults (Supplementary Table S1).

### National trends

**Figure 2:**
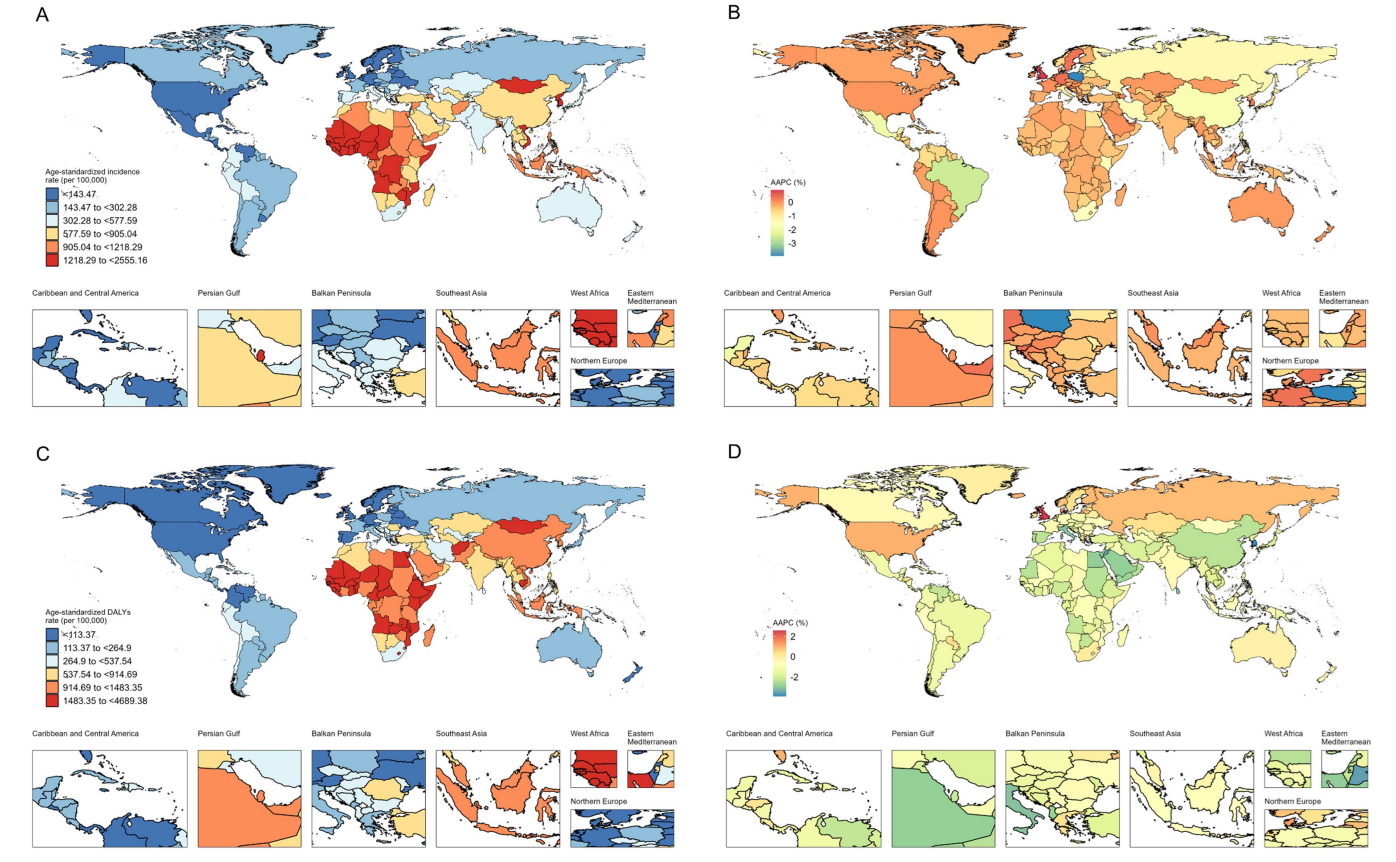
Age-standardized incidence and DALYs of total burden related to hepatitis B in adults aged ≥65 years in 204 countries and territories. **(A)** Age-standardized incidence in 2021; **(B)** AAPC in age-standardized incidence from 1990 to 2021; **(C)** Age-standardized DALYs in 2021; **(D)** AAPC in age-standardized DALYs from 1990 to 2021. DALYs disability-adjusted life-years, AAPC average annual percentage change.

From 1990 to 2021, incident cases of total burden related to hepatitis B among older adults aged≥65 years increased in nearly all countries and territories, with only five exceptions. For instance, Poland experienced a 47.20% decline, while the United Arab Emirates recorded the most substantial rise (an increase of 777.87%) (Supplementary Table S6; Supplementary Figure S11). Among 204 countries and territories, 182 demonstrated significant decreases in ASIR for total burden related to hepatitis B in this age group, with Poland showing the steepest reduction (AAPC -3.91%), followed by Brazil (AAPC -2.57%) and Mexico (AAPC -1.95%) (Figure 2; Supplementary Table S7). During the same period, 29 countries reported declines in hepatitis B-related DALYs cases, while 185 countries demonstrated reductions in ASDR. The Republic of Korea achieved the largest ASDR decline among older adults aged ≥65 years (AAPC -3.90%), followed by Bermuda (AAPC -3.58%). In contrast, the United Kingdom exhibited the most significant ASDR increase (AAPC 2.58%) (Supplementary Table S7). In 2021, the total burden related to hepatitis B among older adults remained concentrated in Africa. The Philippines exhibited the most elevated ASIR (75,930 per 100,000 population), while Egypt exhibited the most elevated ASDR (4,689.38 per 100,000 population) in this age group (Supplementary Table S8). Country-specific trends in total burden related to hepatitis B prevalence, mortality, and their corresponding AAPC values from 1990 to 2021 are detailed in Supplementary Tables S3, S4.

### Trends between SDI and the total burden related to hepatitis B

**Figure 3:**
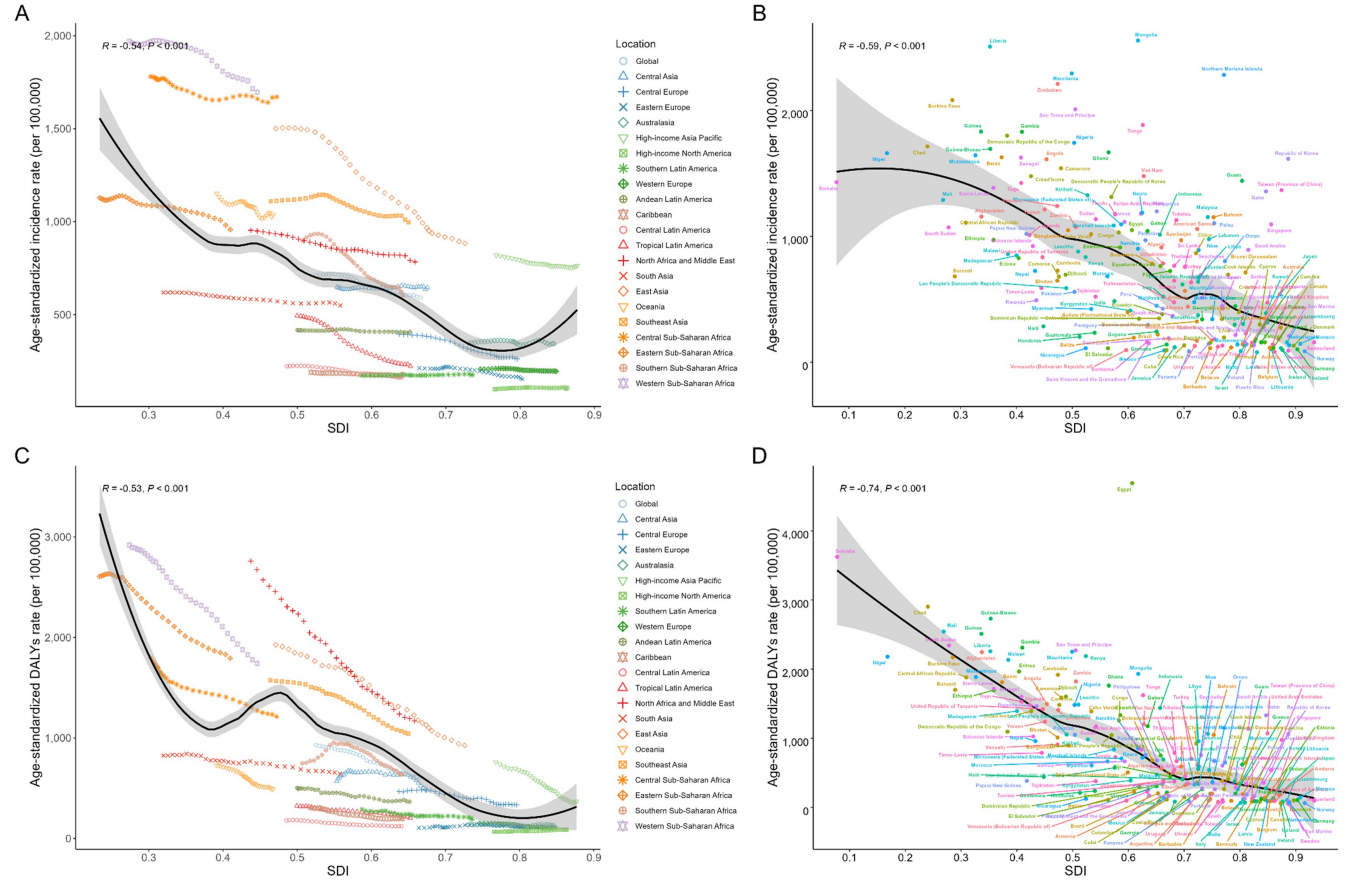
Global, regional, and national levels of total burden related to hepatitis B in adults aged ≥65 years by SDI. **(A)** Age-standardized incidence from 1990 to 2021 at the global and 21 regions levels; **(B)** Age-standardized incidence in 2021 in 204 countries and territories; **(C)** Age-standardized DALYs from 1990 to 2021 at the global and 21 regions levels; **(D)** Age-standardized DALYs in 2021 in 204 countries and territories. For each region, points from left to right depict estimates from each year from 1990 to 2021. Expected trends based on SDI and disease age-standardized rates in all locations were shown as the black line with LOWESS (locally weighted scatterplot smoothing) methods. DALYs disability-adjusted life-years, SDI socio-demographic index.

Globally and regionally, we identified strongly negative epidemiological associations between ASIR and ASDR with SDI (ASIR-SDI: R = −0.54; ASDR-SDI: R = −0.53, both *p* < 0.001) (Figure 3). In low SDI regions, the ASIR of total burden related to hepatitis B among adults aged ≥65 years was higher than in regions with other SDI levels, particularly in Western Sub-Saharan Africa and Central Sub-Saharan Africa (Figure 3). In low SDI regions, Southeast Asia and Western Sub-Saharan Africa exhibited notably higher ASDR, while Southern Sub-Saharan Africa exhibited an initial upward followed by a decline in ASDR over the years, whereas other regions experienced sustained decreases in ASDR (Figure 3).

Among 204 countries and territories, ASIR and ASDR exhibited declining trends as SDI increased. Somalia had the lowest SDI in 2021, yet its ASDR ranked second only to Egypt. The associations of ASPR and ASMR for total burden related to hepatitis B among adults aged 65 and older with SDI at global, regional, and national levels are shown in Supplementary Figure S12.

### Risk factors

**Figure 4:**
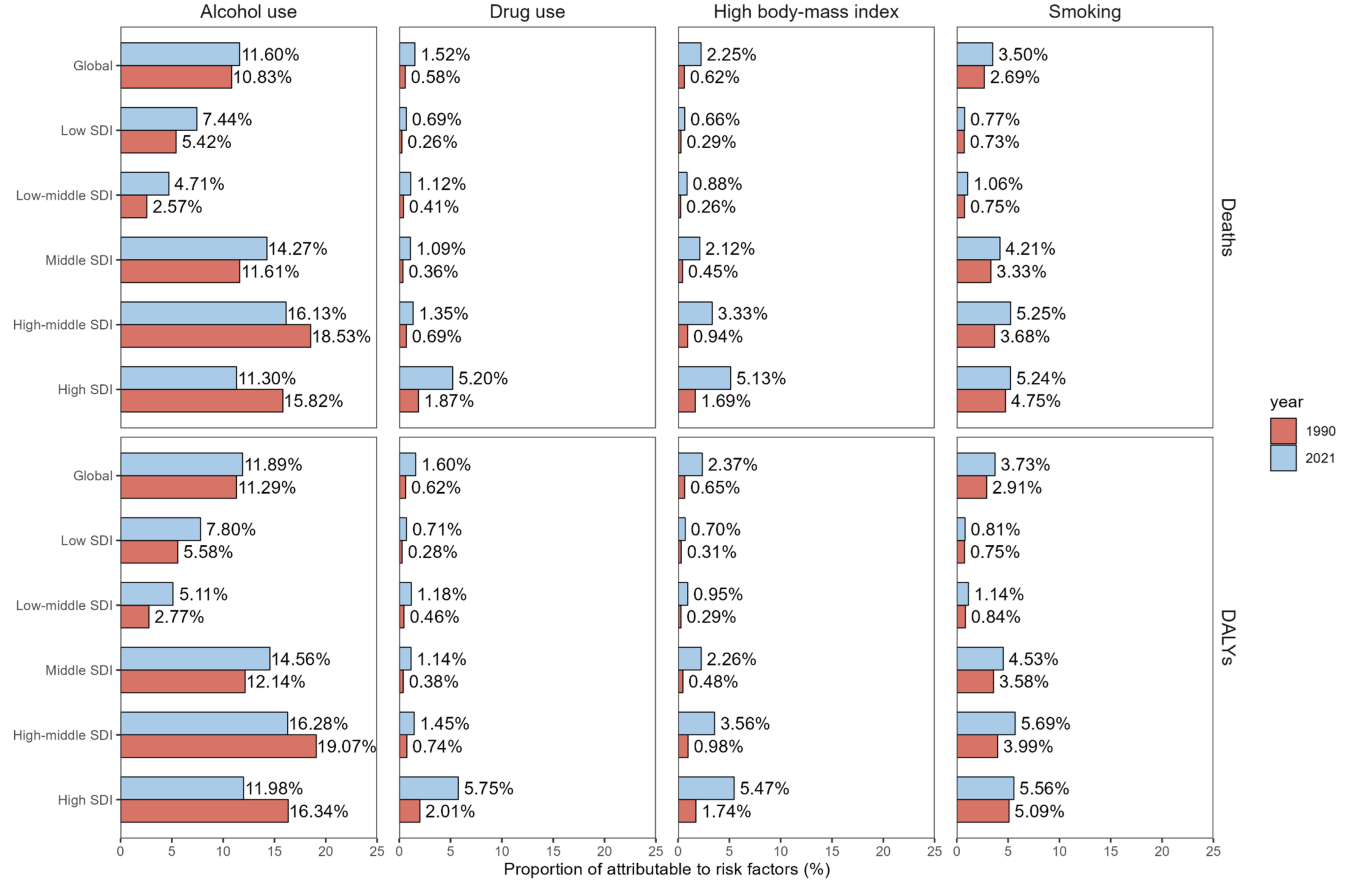
Proportion of deaths and DALYs for total burden related to hepatitis B attributable to alcohol use, drug use, high body-mass index and smoking among people aged ≥65 years from 1990 to 2021 at the global and SDI levels. DALYs disability-adjusted life-years, SDI socio-demographic index.

In 2021, alcohol use, drug use, high BMI, and smoking collectively accounted for 11.60, 1.52, 2.25, and 3.50%, respectively, the DALYs of the total burden related to hepatitis B among older adults aged ≥65 years, as reported in the GBD database (Figure 4). These risk factors contributed 71.69, 9.58, 14.19, and 22.28 DALYs per 100,000 population in this age group. From 1990 to 2021, alcohol use and smoking demonstrated declining contributions to the DALYs (AAPC -1.20% and −0.48%) of the total burden related to hepatitis B while drug use and high BMI exhibited increasing trends (AAPC 1.72 and 2.89%).

Notably, high SDI countries demonstrated the most substantial decrease in alcohol use-attributable total related to hepatitis B burden, with ASDR (AAPC -1.98%) and ASMR (AAPC -1.90%) both showing significant downward trends. In 2021, middle SDI countries recorded the highest ASDR (112.28 per 100,000 population) and ASMR (6.24 per 100,000 population) among older adults. Concurrently, middle SDI countries also showed the steepest increases in high BMI-related burden (AAPC 3.26%) and mortality (AAPC 3.34%), underscoring divergent risk factor dynamics across socioeconomic strata (Supplementary Table S3).

### Projected trends

**Figure 5:**
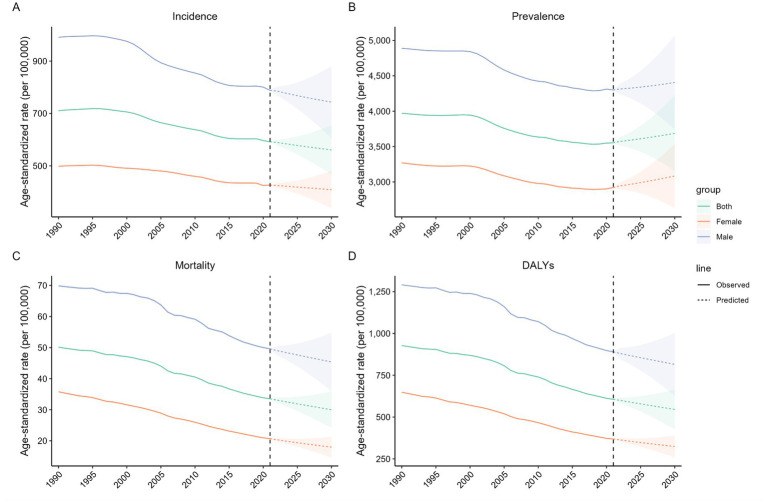
The temporal trends of age-standardized rates of the total burden related to hepatitis B in adults aged 65 years and older from 1990 and 2030 at the global level. **(A)** Age-standardized incidence; **(B)** Age-standardized prevalence; **(C)** Age-standardized mortality; **(D)** Age-standardized DALYs. The colorful shadow denotes the 95% highest density interval of prediction values. The predictive mean value is shown as a colorful dashed line. The vertical dashed line indicates where the prediction starts. DALYs disability-adjusted life-years.

This study utilized the BAPC model to project total burden related to hepatitis B trends among older adults aged ≥65 years beyond 2021. Results indicate that only the ASIR, ASDR, and ASMR are expected to rise (Figure 5). Between 2022 and 2030, the ASIR in this population is projected to decline by 5.25%, with male rates decreasing from 788.28 to 743.55 per 100,000 population (5.67% decrease) and female rates declining from 426.79 to 408.70 per 100,000 population (3.81% decrease) (Supplementary Table S8). Concurrently, the global ASDR is anticipated to fall by 9.95%, with males experiencing a more pronounced decline compared to females. In contrast, all gender groups are expected to exhibit minor increases in ASPR. The ASMR among older adults is projected to continue decreasing, dropping from 33.53 in 2021 to 30.01 in 2030 per 100,000 population (9.34% decrease) (Supplementary Table S8), underscoring divergent trajectories in incidence and mortality burden over the coming decade.

## Discussion

HBV infection and its associated complications continue to pose a significant global public health challenge. This study, leveraging GBD 2021 data, provides a comprehensive analysis of total burden related to hepatitis B among older adults aged ≥65 years from 1990 to 2021, examining trends in incidence, prevalence, mortality, and DALYs. Stratified analyses were conducted by sex, age group, SDI, country, geographic region, and risk factors, with projections to 2030. This study revealed significant global declines in the ASIR and ASDR of total burden related to hepatitis B from 1990 to 2021. The ASIR and ASDR were persistently elevated in males compared to females throughout all age subgroups. Notably, ASIR and ASDR decreased variably among all age groups. A critical observation was the exclusive rise in ASIR within the high SDI group, contrasting with stable or declining trends in other SDI categories. Encouragingly, no increases in ASDR were observed across any sex, age group, or SDI stratum. While most world regions achieved progressive ASMR reductions, Eastern Europe and High-income North America demonstrated stagnant mortality patterns. Projections using the BAPC model anticipate continued reductions in ASIR, ASDR, and ASMR among older adults aged ≥65 years from 2022 to 2030, underscoring the potential for sustained progress in mitigating total burden related to hepatitis B burden in aging populations globally. This study not only offers novel perspectives on understanding the global burden and long-term trends of total burden related to hepatitis B among older adults aged ≥65 years but also provides critical references and strategic guidance for public health policymakers.

Global data revealed that from 1990 to 2021, new cases showed a marked increase, while the ASIR demonstrated a moderate decline. This divergence was particularly pronounced in the 65–69 years age group. Since 1992, the WHO has recommended integrating the hepatitis B vaccine into routine immunization services through the Expanded Program on Immunization. The significant decline in infection rates among younger populations has consequently driven the reduction in ASIR. However, the observed disparity likely reflects the global population aging trend (24). United Nations projections estimate that the population aged ≥65 years will reach 1.6 billion by 2050, rising from 10% of the global population in 2021 to 16% (25), which amplifies the absolute number of cases even as age-standardized rates decline. The ASIR eliminates the impact of population aging on overall incidence rates by adjusting for age structure.

Greater attention should be directed toward mitigating the hepatitis B burden among males compared to females. From 1990 to 2021, males exhibited higher incident cases, greater magnitude of increase, and elevated ASIR than females, despite sustained declines in these metrics for both genders. Recent surveillance data from the 2024 Global Hepatitis Report indicate that 58% of the global burden of chronic hepatitis B and C infections are concentrated in male populations (26). A comprehensive retrospective study in China spanning 1973 to 2021 demonstrated significantly elevated HBsAg positivity in males relative to females, albeit with a progressive attenuation of this gender disparity over time (27). These findings align with our study, which may be explained by the role of estrogen in suppressing viral replication and reducing serum HBV levels. Alcohol consumption constitutes a major modifiable risk factor for hepatitis B. Heavy Episodic Drinking (HED), operationally defined as the consumption of ≥60 grams of pure alcohol per single drinking occasion within the past month, demonstrates significant gender-based variations. When considering only drinkers, the prevalence of HED among all adults is 38%, with 27% among women and 45% among men (28). This disparity in HED prevalence between genders may partially explain the differential hepatitis B incidence rates between males and females.

Globally, incident cases of total burden related to hepatitis B among all age groups aged ≥65 years increased by at least half from 1990 to 2021, likely attributable to the global population aging. In the United States, self-reported hepatitis B vaccination coverage (receipt of ≥3 doses) in 2018 indicated adults aged ≥50 years had a significantly lower rate of 19.1%. These findings highlight the relatively low hepatitis B vaccination rates among older adult populations (29). The ASIR, ASDR, ASPR, and ASMR of total burden related to hepatitis B exhibit an overall declining trend with advancing age. This pattern likely reflects the more stable lifestyle and behavioral patterns of older adults, which significantly attenuate their risk of HBV exposure through blood contact, sexual transmission, and other high-risk routes (30). Additionally, diminished engagement in activities associated with new HBV infections in older populations further contributes to these observed decreases. The ASMR declined significantly across all age groups, while ASPR showed only a marginal increase in the ≥95 years subgroup.

Notably, although the number of new cases has increased significantly throughout all SDI groups, the ASIR among the older adults in high SDI countries has bucked the trend by rising, while other SDI groups have shown declines. This may be related to the relatively adequate healthcare infrastructure in the region and the extended life expectancy among the older adults, which lead to more opportunities for chronic hepatitis B patients to develop related diseases (31). Among these, low SDI countries exhibited the smallest decline, and middle SDI countries demonstrated the largest decrease. Regardless of SDI levels, the extent of ASIR decline among older adults aged >65 years was generally smaller compared to the overall population. Among low to middle SDI countries, persistent barriers such as inadequate sanitation infrastructure and limited vaccination coverage continue to hinder progress in hepatitis prevention and treatment. Although more effective antiviral regimens have become increasingly accessible in low SDI regions, their implementation remains constrained by the high costs of long-term treatment and diagnostic testing (32). Additionally, factors including limited healthcare resources, insufficient health education, and low vaccine awareness contribute to suboptimal hepatitis B vaccination coverage (33, 34). Behavioral risk factors and environmental exposures may further exacerbate hepatitis B risks in these settings (34, 35).

From 1990 to 2021, Andean Latin America experienced the most pronounced surge in incident cases of total burden related to hepatitis B, likely attributable to the region’s persistent social crises and heightened uncertainty—a complex landscape that has catastrophically undermined public health systems (36). Among all 21 GBD regions, East Asia demonstrated remarkable declines in ASIR and ASDR. China played a pivotal role in driving these reductions across the region. Aging populations, characterized by immune decline and heightened healthcare dependency, face elevated HBV-related risks. To eliminate unsafe vaccination practices, China introduced auto-disposable syringes in 2007 and phased out all reusable injection equipment by 2010 (37), significantly curbing HBV transmission rates.

At the national level, hepatitis B among older adults aged ≥65 years demonstrates the most pronounced geographic clustering in Africa, with the Philippines having the highest ASIR and Egypt having the highest ASDR. The disparities in HBV burden across countries and regions can be attributed to several interconnected factors. Underdeveloped sanitation infrastructure and fragmented public health systems in low SDI regions hinder effective HBV transmission control (38). Vaccination coverage remains inadequate in these areas; for instance, while global three-dose infant vaccine coverage exceeds 80%, birth-dose coverage in Africa remains only 38% (39). Furthermore, access to HBV testing and therapeutic interventions continues to be constrained throughout the African continent (40). Low SDI countries often lack robust health education programs, leading to deficient public knowledge of hepatitis B transmission mechanisms, prophylactic approaches, and therapeutic regimens (34). Unhealthy lifestyle choices and environmental factors further exacerbate HBV infection risks (34). The study results demonstrated a significant negative correlation between the ASIR and ASDR of the total burden related to hepatitis B and the SDI at both global and regional levels, underscoring the critical role of socioeconomic development in shaping the disease burden.

Among older adults aged ≥65 years, the total burden related to hepatitis B is attributable to four major risk factors: alcohol use, drug use, high BMI, and smoking. In high SDI countries, alcohol use demonstrated the most pronounced decrease in burden, although this does not imply low alcohol consumption rates in these nations. Regions with high SDI demonstrated the highest prevalence of alcohol use. However, at equivalent levels of alcohol consumption, socioeconomically disadvantaged individuals and low income nations experienced disproportionately higher alcohol-attributable disease burden and mortality (41). Smoking has also demonstrated a significant reduction in disease burden. As socioeconomic status improves, heightened public awareness of smoking’s health hazards has prompted more individuals to adopt smoking cessation measures, effectively mitigating smoking-related health risks. Conversely, drug use and elevated BMI are exhibiting increasing trends. The health consequences of drug abuse manifest as chronic effects, with sustained immune suppression and progressive exacerbation of hepatic impairment rendering older adults individuals more susceptible to HBV-related diseases in later life (42). From 1990 to 2021, overweight and obesity rates increased globally, across all regions, and in every country (43). Co-infections with HIV, HCV, and HBV are relatively common among people who inject drugs, due to their shared transmission routes. Co-infection can accelerate disease progression, complicate treatment regimens, and increase mortality. The overlapping burden of these infections suggests that integrated service delivery models—such as combined testing, treatment, and harm reduction programs—could enhance both efficiency and effectiveness. Future studies should aim to explicitly quantify the burden attributable to co-infections (44).

The BAPC model predicts that the total burden related to hepatitis B burden among older adults aged ≥65 years after 2021 will show a rising trend only in the ASPR. Globally, approximately 39.57% of liver cancer mortality is attributable to hepatitis B (45). Antiviral therapy can suppress viral replication and demonstrate protective effects against hepatocellular carcinoma (HCC), while hepatitis B vaccination remains the most effective method to lower HCC incidence (46). The widespread adoption of vaccination and advancements in antiviral therapies have prolonged the survival of chronic hepatitis B patients (47). This trend reflects both the success of interventions in extending survival and the growing prevalence of long-term HBV carriers within aging populations. To reduce the total burden related to hepatitis B among older adults, it is essential to optimize vaccination strategies by conducting serological screening (HBsAg/anti-HBc) and implementing catch-up vaccination programs for unvaccinated older adults in high SDI countries—particularly males—and older adults in low SDI countries who lacked access to early vaccination coverage. Targeted risk factor control measures should be prioritized, including alcohol use restrictions (especially for males) and the promotion of harm reduction initiatives such as needle exchange programs (e.g., the Dutch model) for people who inject drugs. National governments should negotiate pricing agreements and establish centralized procurement plans to ensure affordable access to essential medications. Additionally, efforts must be intensified to improve the accessibility of prevention, screening, diagnosis, and treatment services in low- and middle-income countries, ensuring equitable healthcare delivery for aging populations worldwide.

This study has certain limitations that need to be taken into account when interpreting the findings. First, the estimation of disease burden largely depends on the quality and availability of the GBD 2021 data. The accuracy and reliability of the estimates in this study depend on the quality of the input data for modeling, which may be affected by insufficient disease screening or varying diagnostic criteria in some low and low-middle SDI regions, and potential misdiagnosis and missed diagnosis due to inadequate healthcare systems in underdeveloped countries may lead to underestimated case numbers, while the number of cases in high SDI countries is increased due to incidental diagnosis. The population base of the ≥95 years age group is small, and collecting health data for this group is challenging. Particularly in low-SDI regions, the imperfect registration systems for older adults may lead to uncertainties in the assumption of data completeness for this age group. Additionally, the historical span of our research, encompassing data from 1990 onwards, might be affected by evolving diagnostic criteria and medical technologies over the years. Finally, our projections up to 2030, though based on rigorous statistical models, but due to the information lag in this database and certain assumptions, which external factors could influence, the prediction results may not be accurate enough. However, our results still have significant public health implications for controlling the total burden related to hepatitis B globally.

## Conclusion

From 1990 to 2021, the total burden related to hepatitis B among older adults aged ≥65 years declined significantly worldwide, particularly in East Asia. Males consistently faced a more severe burden compared to females. Low SDI countries, especially the Philippines and Egypt, continue to experience a substantial burden and require targeted interventions. Alcohol use, drug use, high BMI, and smoking remain major challenges in total burden related to hepatitis B prevention and control. Despite the overall global burden decline, HBV and its complications persist as a critical public health threat. To mitigate the overall hepatitis B burden among the older adults, it is imperative to optimize vaccination strategies, implement control measures targeting risk factors, and ensure governmental commitments to guaranteeing the affordability of essential medicines and equitable access to healthcare services for the aging global population. This study provides a theoretical foundation for formulating public health policies by revealing the epidemiological characteristics and evolving trends of the total burden related to hepatitis B across diverse populations, regions, and risk factors, thereby facilitating the achievement of the global hepatitis B elimination goal.

## Data Availability

The original contributions presented in the study are included in the article/Supplementary material, further inquiries can be directed to the corresponding authors.
