# Correction: Global, regional, and national total burden related to hepatitis B and attributable risk factors in adults aged 65 years and older from 1990 to 2021 and projection to 2030

**DOI:** 10.3389/fpubh.2026.1782146

**Published:** 2026-01-29

**Authors:** Linying Gao, Yanfeng Ren, Songyue Hu, Yifan Zhang, Hongjing Bai, Jinbo Li, Keke Wang, Suping Wang, Yongliang Feng

**Affiliations:** 1 School of Public Health, Shanxi Medical University, Taiyuan, China; 2 Center of Clinical Epidemiology and Evidence Based Medicine, Shanxi Medical University, Taiyuan, China; 3 MOE Key Laboratory of Coal Environmental Pathogenicity and Prevention, Shanxi Medical University, Taiyuan, China; 4 Research Center for Reverse Etiology, Workstation of Academician, Shanxi Medical University, Taiyuan, China; 5 First Hospital of Shanxi Medical University, Taiyuan, China

**Keywords:** hepatitis B virus, global burden of disease, disability-adjusted life years, epidemiology, Joinpoint regression analysis, Bayesian age-period-cohort

In the **Acknowledgements**, the acknowledgement for the Shanxi Province Higher Education “Ten Billion Project” Science and Technology Guidance Project was erroneously omitted. The Acknowledgement should read:

The authors gratefully acknowledge the support of the Shanxi Province Higher Education “Ten Billion Project” Science and Technology Guidance Project. Data for this study were sourced from the Global Burden of Disease database maintained by the Institute for Health Metrics and Evaluation (IHME). The authors gratefully acknowledge the IHME team for their efforts in data collection, curation, and public sharing.

The original version of this article has been updated.

