# Supplementary material for: Global, regional, and national total burden related to hepatitis B and attributable risk factors in adults aged 65 years and older from 1990 to 2021 and projection to 2030
Source: Front Public Health. 2025 Sep 25;13:1654356. doi: 10.3389/fpubh.2025.1654356 (PMC12507896; doi:10.3389/fpubh.2025.1654356)

Supplementary materials

[Table S1 Age-standardized incidence and DALYs of total burden related to hepatitis B among people aged ≥65 years between 1990 and 2021 at the global and regional levels 1](#_Toc31275)

Table S2 The AAPCs of age-standardized incidence , DALYs, Prevalence, and Mortality of total burden related to hepatitis B among people aged ≥65 years from 1990 to 2021 at the global and regional levels 1

Table S3 Main risk factors for age-standardized DALYs and mortality of total burden related to hepatitis B among people aged ≥65 years from 1990 to 2021 1

Table S4 Age-standardized prevalence and mortality of total burden related to hepatitis B in adults aged ≥65 years between 1990 and 2021 at the global and regional levels 1

[Table S5 Age-standardized incidence and DALYs of total burden related to hepatitis B in adults aged ≥65 years between 1990 and 2021 in 204 countries and territories 5](#_Toc23938)

[Table S6 Age-standardized prevalence and mortality of total burden related to hepatitis B in adults aged ≥65 years between 1990 and 2021 in 204 countries and territories 28](#_Toc26156)

[Table S7 The AAPCs of age-standardized incidence, prevalence, mortality, and DALYs of total burden related to hepatitis B in adults aged ≥65 years from 1990 to 2021 in 204 countries and territories 52](#_Toc20365)

[Table S8 Prediction of age-standardized incidence, prevalence, mortality, and DALYs of total burden related to hepatitis B in adults aged ≥65 years from 2022 to 2030 68](#_Toc30458)

[Figure S1 The changes in the proportion of cases among total burden related to hepatitis B patients aged ≥65 years to the overall age of patients from 1990 to 2021 71](#_Toc19697)

[Figure S2 Temporal trend of age-standardized incidence, prevalence, mortality, and DALYs for total burden related to hepatitis B patients aged ≥65 years to the overall age of patients from 1990 to 2021 72](#_Toc16775)

[Figure S3 Temporal trend of age-standardized incidence, prevalence, mortality, and DALYs of total burden related to hepatitis B in adults aged ≥65 years from 1990 to 2021 at the global and SDI levels by sex 73](#_Toc11977)

[Figure S4 Temporal trend of age-standardized incidence, prevalence, mortality, and DALYs of total burden related to hepatitis B in children and adolescents from 1990 to 2021 at the global and SDI levels by sex 74](#_Toc15009)

[Figure S5 The AAPCs of age-standardized incidence, prevalence, mortality, and DALYs of total burden related to hepatitis B in adults aged ≥65 years from 1990 to 2021 at the global and SDI levels by sex 75](#_Toc15914)

[Figure S6 The AAPCs of age-standardized incidence, prevalence, mortality, and DALYs of total burden related to hepatitis B in adults aged ≥65 years from 1990 to 2021 by sex and age 76](#_Toc6286)

[Figure S7 Prevalence and mortality of total burden related to hepatitis B in adults aged ≥65 years from 1990 to 2021 at the global level 77](#_Toc3033)

[Figure S8 Temporal trend of age-standardized incidence, prevalence, mortality, and DALYs of total burden related to hepatitis B aged ≥65 years and overall age of patients from 1990 to 2021 at the global and SDI levels 78](#_Toc22082)

[Figure S9 The AAPCs of age-standardized incidence, prevalence, mortality, and DALYs of total burden related to hepatitis B in adults aged ≥65 years from 1990 to 2021 at the regional level by sex 79](#_Toc18200)

[Figure S10 The cases of change of total burden related to hepatitis B in adults aged ≥65 years between 1990 and 2021 in 204 countries and territories 80](#_Toc3476)

[Figure S11 Age-standardized prevalence and mortality of total burden related to hepatitis B in adults aged ≥65 years in 204 countries and territories 81](#_Toc5013)

[Figure S12 Global, regional, and national levels of total burden related to hepatitis B in adults aged ≥65 years by SDI 82](#_Toc18404)

Table S1 Age-standardized incidence and DALYs of total burden related to hepatitis B among people aged ≥65 years between 1990 and 2021 at the global and regional levels

| Characteristics | Incidence | | | | |  | DALYs | | | | |
| --- | --- | --- | --- | --- | --- | --- | --- | --- | --- | --- | --- |
|  | Cases in 1990 | Age-standardized rate in 1990 (per 100 000) | Cases in 2021 | Age-standardized rate in 2021 (per 100 000) | Cases  change (%) |  | Cases in 1990 | Age-standardized rate in 1990 (per 100 000) | Cases in 2021 | Age-standardized rate in 2021 (per 100 000) | Cases  change (%) |
| Global | 2391470 (1261914-3939760) | 710.53 (372.98-1173.88) | 4617673 (2413783-7751316) | 591.79 (308.45-995.21) | 93.09 |  | 3111129 (2530544-3816664) | 927.65 (752.80-1140.73) | 4709831 (3773559-5729571) | 605.93 (485.45-737.11) | 51.39 |
| Sex |  |  |  |  |  |  |  |  |  |  |  |
| Female | 941324 (436550-1799680) | 498.24 (230.41-952.95) | 1799632 (822423-3568933) | 426.79 (194.94-846.44) | 91.18 |  | 1218908 (950583-1529412) | 648.12 (504.14-815.14) | 1551511 (1187355-1957193) | 368.70 (282.25-465.04) | 27.29 |
| Male | 1450146 (735289-2604931) | 990.79 (499.48-1783.12) | 2818040 (1433721-5125766) | 788.28 (398.76-1438.68) | 94.33 |  | 1892220 (1543805-2315359) | 1290.72 (1050.20-1584.42) | 3158320 (2530590-3853776) | 889.58 (712.74-1085.91) | 66.91 |
| Age |  |  |  |  |  |  |  |  |  |  |  |
| 65 to 69 | 1064261 (587645-1709246) | 860.99 (475.40-1382.78) | 1938546 (1025091-3145777) | 702.77 (371.62-1140.43) | 82.15 |  | 1350144 (1097540-1647873) | 1092.27 (887.91-1333.13) | 1884296 (1507725-2291322) | 683.11 (546.59-830.66) | 39.56 |
| 70 to 74 | 673676 (360617-1101905) | 795.73 (425.95-1301.55) | 1272765 (704153-2163652) | 618.33 (342.09-1051.14) | 88.93 |  | 877065 (718307-1073186) | 1035.97 (848.45-1267.63) | 1276202 (1018574-1561445) | 620.00 (494.84-758.57) | 45.51 |
| 75 to 79 | 392540 (191549-676158) | 637.70 (311.18-1098.45) | 736014 (359046-1276958) | 558.07 (272.24-968.24) | 87.50 |  | 530370 (437335-645531) | 861.61 (710.47-1048.70) | 794488 (647961-953735) | 602.41 (491.31-723.16) | 49.80 |
| 80 to 84 | 181604 (83387-313882) | 513.36 (235.72-887.27) | 418438 (205827-705736) | 477.76 (235.01-805.79) | 130.41 |  | 239697 (190268-304003) | 677.57 (537.85-859.35) | 462199 (369375-563922) | 527.73 (421.74-643.87) | 92.83 |
| 85 to 89 | 63058 (31045-110238) | 417.30 (205.44-729.52) | 183175 (87340-331284) | 400.63 (191.02-724.57) | 190.49 |  | 89930 (70050-113140) | 595.13 (463.57-748.73) | 212023 (169045-256452) | 463.72 (369.73-560.90) | 135.76 |
| 90 to 94 | 13818 (6659-23482) | 322.46 (155.40-547.99) | 55358 (26753-102129) | 309.45 (149.55-570.89) | 300.63 |  | 19770 (14448-26621) | 461.36 (337.16-621.24) | 64676 (49941-81049) | 361.53 (279.17-453.06) | 227.14 |
| 95 plus | 2512 (1012-4849) | 246.71 (99.43-476.26) | 13377 (5574-25779) | 245.43 (102.26-472.98) | 432.56 |  | 4151 (2596-6308) | 407.71 (254.97-619.61) | 15947 (10937-21647) | 292.59 (200.66-397.17) | 284.19 |
| SDI |  |  |  |  |  |  |  |  |  |  |  |
| Low SDI | 198780 (103568-338184) | 1174.30 (604.00-2006.21) | 382409 (199239-636087) | 1005.77 (518.26-1682.35) | 92.38 |  | 357875 (265927-467828) | 2099.56 (1549.46-2760.28) | 479073 (358709-613569) | 1270.55 (950.84-1626.02) | 33.87 |
| Low-middle SDI | 365170 (194801-604106) | 800.54 (422.82-1330.05) | 783663 (439833-1266082) | 669.28 (373.17-1085.91) | 114.60 |  | 660288 (463977-914294) | 1489.04 (1039.90-2063.58) | 1038104 (750199-1370306) | 895.14 (645.62-1181.83) | 57.22 |
| Middle SDI | 905564 (463987-1511107) | 1117.84 (566.28-1875.59) | 1779486 (907976-3038550) | 755.24 (382.80-1296.67) | 96.51 |  | 1118335 (943530-1329539) | 1397.05 (1171.84-1674.25) | 1807967 (1464927-2181780) | 779.29 (631.14-941.58) | 61.67 |
| High-middle SDI | 599076 (307179-1003691) | 699.97 (356.62-1176.04) | 1017058 (495023-1783665) | 546.33 (264.83-961.15) | 69.77 |  | 661875 (555469-781810) | 772.92 (647.82-914.16) | 943133 (758741-1142211) | 509.35 (409.92-616.98) | 42.49 |
| High SDI | 321219 (179497-523042) | 308.01 (172.24-501.39) | 652442 (372900-1052750) | 324.32 (186.12-522.05) | 103.11 |  | 310607 (249067-381933) | 297.67 (238.54-366.17) | 438642 (343405-549999) | 217.82 (171.00-272.66) | 41.22 |
| Region |  |  |  |  |  |  |  |  |  |  |  |
| Andean Latin America | 6722 (3788-10710) | 416.76 (234.15-665.07) | 20516 (12552-31656) | 407.87 (249.19-629.78) | 205.23 |  | 8156 (4822-13165) | 503.72 (297.99-812.84) | 18423 (10805-29663) | 366.15 (214.80-589.55) | 125.87 |
| Australasia | 7809 (3106-14412) | 346.56 (137.27-641.09) | 17629 (7274-33765) | 343.09 (142.14-654.02) | 125.74 |  | 3158 (2017-4431) | 138.83 (88.63-194.86) | 6752 (4472-9424) | 131.80 (87.42-183.58) | 113.80 |
| Caribbean | 4343 (1942-7944) | 189.49 (84.28-347.47) | 8420 (3790-14917) | 177.86 (80.13-314.78) | 93.86 |  | 6965 (3962-11341) | 305.40 (173.70-497.41) | 9314 (5352-15260) | 196.73 (113.07-322.16) | 33.72 |
| Central Asia | 22735 (12688-38431) | 647.63 (360.66-1095.74) | 39871 (22726-63406) | 645.08 (365.22-1033.38) | 75.37 |  | 20063 (12953-29508) | 570.14 (367.44-839.75) | 29861 (18414-46291) | 479.10 (294.88-745.15) | 48.84 |
| Central Europe | 52839 (30553-86697) | 394.50 (227.15-647.18) | 58711 (31032-97816) | 262.58 (138.60-437.73) | 11.11 |  | 64134 (41720-91083) | 467.41 (304.27-664.03) | 74815 (47280-109687) | 334.30 (211.37-489.96) | 16.65 |
| Central Latin America | 14470 (7106-25599) | 221.89 (108.25-393.65) | 34652 (15056-66055) | 162.23 (70.17-309.98) | 139.48 |  | 11741 (8177-16617) | 179.57 (125.08-254.11) | 26204 (18424-36845) | 122.51 (86.16-172.20) | 123.18 |
| Central Sub-Saharan Africa | 28494 (11863-53435) | 1780.84 (707.72-3369.84) | 61192 (28939-111489) | 1671.36 (765.10-3090.75) | 114.75 |  | 29665 (18005-45757) | 1796.56 (1087.59-2767.27) | 45713 (26698-71060) | 1213.78 (709.35-1886.34) | 54.10 |
| East Asia | 1058329 (519972-1815784) | 1500.72 (718.12-2598.47) | 1834938 (845242-3279620) | 882.81 (402.13-1589.84) | 73.38 |  | 1331873 (1146202-1541809) | 1926.90 (1656.32-2234.87) | 1882297 (1520064-2305137) | 926.34 (748.47-1134.89) | 41.33 |
| Eastern Europe | 50113 (21498-97168) | 212.22 (90.63-411.13) | 51612 (21878-101093) | 152.99 (64.39-300.97) | 2.99 |  | 25272 (18825-33701) | 105.80 (78.71-141.34) | 39778 (29785-52980) | 116.62 (87.37-154.97) | 57.40 |
| Eastern Sub-Saharan Africa | 63467 (32055-109466) | 1128.22 (557.80-1961.27) | 117248 (58832-200348) | 957.56 (472.55-1649.31) | 84.74 |  | 147725 (111392-187719) | 2606.10 (1958.06-3322.71) | 217569 (161954-279895) | 1792.12 (1326.04-2308.34) | 47.28 |
| High-income Asia Pacific | 144269 (84858-227117) | 819.55 (479.98-1292.40) | 336180 (204728-525877) | 763.11 (469.09-1188.37) | 133.02 |  | 134595 (105511-164536) | 760.17 (595.11-930.38) | 166732 (124227-216057) | 371.18 (277.80-479.69) | 23.88 |
| High-income North America | 33981 (15216-59971) | 98.91 (44.35-174.54) | 67033 (31896-116272) | 104.78 (49.84-181.42) | 97.27 |  | 23195 (17774-30396) | 67.51 (51.70-88.54) | 57086 (44615-72096) | 89.32 (69.83-112.76) | 146.11 |
| North Africa and Middle East | 121041 (73255-191941) | 953.57 (572.10-1515.25) | 272124 (168702-413755) | 783.57 (484.02-1195.15) | 124.82 |  | 329020 (220479-458889) | 2759.64 (1834.02-3873.61) | 399238 (269749-551183) | 1168.95 (787.68-1614.80) | 21.34 |
| Oceania | 2382 (1061-4436) | 1152.03 (505.52-2164.75) | 5253 (2420-9854) | 1036.12 (472.27-1958.09) | 120.49 |  | 1542 (987-2489) | 728.90 (469.38-1167.22) | 2529 (1714-3632) | 491.21 (333.69-701.88) | 64.02 |
| South Asia | 255855 (137052-429429) | 619.19 (328.14-1043.10) | 669490 (376895-1070747) | 549.42 (307.58-881.75) | 161.67 |  | 341568 (241064-495820) | 825.17 (579.08-1201.19) | 774703 (538742-1025981) | 645.07 (446.68-856.03) | 126.81 |
| Southeast Asia | 210884 (113523-345003) | 1109.94 (590.24-1824.53) | 522631 (289663-844335) | 996.28 (546.75-1618.21) | 147.83 |  | 295685 (202791-437499) | 1573.94 (1069.73-2342.64) | 535593 (387128-710781) | 1043.82 (752.99-1384.11) | 81.14 |
| Southern Latin America | 7118 (4315-11434) | 171.71 (103.73-275.89) | 14206 (8409-22395) | 175.69 (104.11-276.84) | 99.58 |  | 12235 (6522-20321) | 291.39 (155.36-484.38) | 15465 (8852-24314) | 192.05 (109.88-301.89) | 26.41 |
| Southern Sub-Saharan Africa | 19587 (10020-33029) | 898.31 (454.91-1520.71) | 28265 (14785-47671) | 603.56 (313.98-1021.41) | 44.30 |  | 16008 (10645-23524) | 748.53 (497.42-1101.03) | 29192 (22031-37888) | 636.55 (481.08-825.65) | 82.35 |
| Tropical Latin America | 36027 (18466-61415) | 493.99 (251.08-844.39) | 49861 (22189-89310) | 222.32 (98.34-399.04) | 38.40 |  | 23847 (18402-29923) | 324.27 (249.86-407.08) | 46247 (35959-59058) | 204.93 (159.25-261.62) | 93.93 |
| Western Europe | 114784 (55404-200877) | 206.72 (100.18-361.67) | 170443 (84724-294119) | 194.21 (98.20-332.35) | 48.49 |  | 83478 (58102-116901) | 150.10 (104.24-210.36) | 91337 (62278-128212) | 104.80 (71.48-146.87) | 9.41 |
| Western Sub-Saharan Africa | 136220 (71865-232715) | 1971.39 (1027.71-3376.50) | 237397 (124271-396726) | 1696.27 (874.89-2853.80) | 74.27 |  | 201203 (145234-271238) | 2918.10 (2103.92-3931.45) | 240982 (182698-304591) | 1741.89 (1324.08-2195.49) | 19.77 |

AAPC average annual percentage change, DALYs disability-adjusted life-years, SDI socio-demographic index

Table S2 The AAPCs of age-standardized incidence , DALYs, Prevalence, and Mortality of total burden related to hepatitis B among people aged ≥65 years from 1990 to 2021 at the global and regional levels

| Characteristics | Incidence AAPC (95% CI) | P value |  | Prevalence AAPC (95% CI) | P value |  | Mortality AAPC (95% CI) | P value |  | DALYs AAPC (95% CI) | P value |
| --- | --- | --- | --- | --- | --- | --- | --- | --- | --- | --- | --- |
| Global | -0.57 (-0.59 to -0.56) | <0.001 |  | -0.35 (-0.37 to -0.34) | <0.001 |  | -1.34 (-1.37 to -1.30) | <0.001 |  | -1.39 (-1.41 to -1.37) | <0.001 |
| Sex |  |  |  |  |  |  |  |  |  |  |  |
| Female | -0.51 (-0.54 to -0.49) | <0.001 |  | -0.36 (-0.38 to -0.35) | <0.001 |  | -1.77 (-1.81 to -1.74) | <0.001 |  | -1.82 (-1.84 to -1.79) | <0.001 |
| Male | -0.72 (-0.73 to -0.70) | <0.001 |  | -0.41 (-0.42 to -0.40) | <0.001 |  | -1.15 (-1.19 to -1.11) | <0.001 |  | -1.21 (-1.24 to -1.18) | <0.001 |
| Age |  |  |  |  |  |  |  |  |  |  |  |
| 65 to 69 | -0.63 (-0.65 to -0.61) | <0.001 |  | -0.45 (-0.46 to -0.43) | <0.001 |  | -1.48 (-1.52 to -1.45) | <0.001 |  | -1.48 (-1.51 to -1.45) | <0.001 |
| 70 to 74 | -0.83 (-0.85 to -0.81) | <0.001 |  | -0.57 (-0.59 to -0.55) | <0.001 |  | -1.68 (-1.71 to -1.65) | <0.001 |  | -1.67 (-1.69 to -1.64) | <0.001 |
| 75 to 79 | -0.43 (-0.47 to -0.40) | <0.001 |  | -0.18 (-0.20 to -0.16) | <0.001 |  | -1.15 (-1.20 to -1.11) | <0.001 |  | -1.15 (-1.20 to -1.11) | <0.001 |
| 80 to 84 | -0.22 (-0.26 to -0.20) | <0.001 |  | -0.02 (-0.05 to -0.00) | 0.032 |  | -0.84 (-0.93 to -0.73) | <0.001 |  | -0.77 (-0.85 to -0.69) | <0.001 |
| 85 to 89 | -0.17 (-0.20 to -0.14) | <0.001 |  | 0.12 (0.11 to 0.13) | <0.001 |  | -0.75 (-0.83 to -0.67) | <0.001 |  | -0.75 (-0.83 to -0.67) | <0.001 |
| 90 to 94 | -0.13 (-0.15 to -0.11) | <0.001 |  | 0.18 (0.16 to 0.19) | <0.001 |  | -0.82 (-0.92 to -0.73) | <0.001 |  | -0.80 (-0.91 to -0.72) | <0.001 |
| 95 plus | 0.01 (-0.01 to 0.03) | 0.159 |  | 0.24 (0.23 to 0.26) | <0.001 |  | -1.06 (-1.13 to -0.98) | <0.001 |  | -1.07 (-1.14 to -0.99) | <0.001 |
| SDI |  |  |  |  |  |  |  |  |  |  |  |
| Low SDI | -0.50 (-0.52 to -0.48) | <0.001 |  | -0.67 (-0.69 to -0.66) | <0.001 |  | -1.52 (-1.54 to -1.50) | <0.001 |  | -1.60 (-1.62 to -1.58) | <0.001 |
| Low-middle SDI | -0.57 (-0.58 to -0.57) | <0.001 |  | -0.75 (-0.76 to -0.74) | <0.001 |  | -1.78 (-1.84 to -1.72) | <0.001 |  | -1.61 (-1.65 to -1.56) | <0.001 |
| Middle SDI | -1.27 (-1.29 to -1.25) | <0.001 |  | -0.94 (-0.95 to -0.93) | <0.001 |  | -1.83 (-1.86 to -1.80) | <0.001 |  | -1.90 (-1.94 to -1.88) | <0.001 |
| High-middle SDI | -0.80 (-0.82 to -0.76) | <0.001 |  | -0.15 (-0.17 to -0.13) | <0.001 |  | -1.25 (-1.31 to -1.19) | <0.001 |  | -1.40 (-1.45 to -1.34) | <0.001 |
| High SDI | 0.16 (0.15 to 0.17) | <0.001 |  | 0.12 (0.12 to 0.13) | <0.001 |  | -0.83 (-0.90 to -0.78) | <0.001 |  | -0.99 (-1.05 to -0.95) | <0.001 |
| Region |  |  |  |  | <0.001 |  |  |  |  |  |  |
| Andean Latin America | -0.09 (-0.11 to -0.07) | <0.001 |  | -0.36 (-0.38 to -0.35) | <0.001 |  | -1.00 (-1.18 to -0.86) | <0.001 |  | -1.03 (-1.12 to -0.91) | <0.001 |
| Australasia | -0.03 (-0.06 to -0.01) | 0.005 |  | -0.24 (-0.24 to -0.23) | <0.001 |  | -0.01 (-0.11 to 0.08) | 0.770 |  | -0.18 (-0.27 to -0.09) | <0.001 |
| Caribbean | -0.20 (-0.21 to -0.19) | <0.001 |  | -0.44 (-0.45 to -0.42) | <0.001 |  | -1.45 (-1.52 to -1.36) | <0.001 |  | -1.36 (-1.42 to -1.29) | <0.001 |
| Central Asia | -0.02 (-0.04 to 0.00) | 0.090 |  | -0.07 (-0.09 to -0.05) | <0.001 |  | -0.61 (-0.70 to -0.52) | <0.001 |  | -0.63 (-0.71 to -0.55) | <0.001 |
| Central Europe | -1.32 (-1.35 to -1.30) | <0.001 |  | -1.15 (-1.16 to -1.15) | <0.001 |  | -1.11 (-1.16 to -1.06) | <0.001 |  | -1.05 (-1.10 to -1.00) | <0.001 |
| Central Latin America | -1.04 (-1.09 to -1.01) | <0.001 |  | -0.63 (-0.64 to -0.62) | <0.001 |  | -1.29 (-1.38 to -1.18) | <0.001 |  | -1.29 (-1.37 to -1.19) | <0.001 |
| Central Sub-Saharan Africa | -0.20 (-0.21 to -0.18) | <0.001 |  | -0.47 (-0.47 to -0.46) | <0.001 |  | -1.27 (-1.29 to -1.25) | <0.001 |  | -1.25 (-1.27 to -1.23) | <0.001 |
| East Asia | -1.71 (-1.73 to -1.70) | <0.001 |  | -0.98 (-0.99 to -0.97) | <0.001 |  | -2.25 (-2.31 to -2.18) | <0.001 |  | -2.33 (-2.39 to -2.28) | <0.001 |
| Eastern Europe | -1.04 (-1.07 to -1.02) | <0.001 |  | -0.78 (-0.81 to -0.75) | <0.001 |  | 0.33 (0.22 to 0.46) | <0.001 |  | 0.32 (0.21 to 0.44) | <0.001 |
| Eastern Sub-Saharan Africa | -0.53 (-0.55 to -0.51) | <0.001 |  | -0.61 (-0.62 to -0.60) | <0.001 |  | -1.12 (-1.14 to -1.10) | <0.001 |  | -1.19 (-1.21 to -1.18) | <0.001 |
| High-income Asia Pacific | -0.23 (-0.24 to -0.22) | <0.001 |  | -0.44 (-0.45 to -0.43) | 0.016 |  | -2.09 (-2.17 to -2.03) | <0.001 |  | -2.25 (-2.32 to -2.19) | <0.001 |
| High-income North America | 0.17 (0.11 to 0.23) | <0.001 |  | -0.03 (-0.04 to -0.00) | <0.001 |  | 0.93 (0.87 to 0.99) | <0.001 |  | 0.90 (0.84 to 0.96) | <0.001 |
| North Africa and Middle East | -0.65 (-0.67 to -0.63) | <0.001 |  | -0.85 (-0.86 to -0.85) | <0.001 |  | -2.89 (-3.02 to -2.82) | <0.001 |  | -2.74 (-2.78 to -2.70) | <0.001 |
| Oceania | -0.35 (-0.38 to -0.32) | <0.001 |  | -0.32 (-0.33 to -0.31) | <0.001 |  | -1.28 (-1.30 to -1.25) | <0.001 |  | -1.29 (-1.31 to -1.26) | <0.001 |
| South Asia | -0.38 (-0.39 to -0.37) | <0.001 |  | -0.54 (-0.54 to -0.53) | <0.001 |  | -0.69 (-0.78 to -0.59) | <0.001 |  | -0.80 (-0.87 to -0.73) | <0.001 |
| Southeast Asia | -0.35 (-0.37 to -0.34) | <0.001 |  | -0.55 (-0.56 to -0.54) | <0.001 |  | -1.28 (-1.30 to -1.26) | <0.001 |  | -1.32 (-1.34 to -1.30) | <0.001 |
| Southern Latin America | 0.06 (0.02 to 0.09) | 0.005 |  | 0.17 (0.17 to 0.18) | <0.001 |  | -1.28 (-1.41 to -1.17) | <0.001 |  | -1.21 (-1.33 to -1.11) | <0.001 |
| Southern Sub-Saharan Africa | -1.29 (-1.33 to -1.26) | <0.001 |  | -1.20 (-1.22 to -1.18) | <0.001 |  | -0.60 (-0.65 to -0.54) | <0.001 |  | -0.56 (-0.62 to -0.50) | <0.001 |
| Tropical Latin America | -2.52 (-2.55 to -2.48) | <0.001 |  | -1.45 (-1.46 to -1.44) | <0.001 |  | -1.50 (-1.58 to -1.42) | <0.001 |  | -1.43 (-1.51 to -1.34) | <0.001 |
| Western Europe | -0.19 (-0.20 to -0.18) | <0.001 |  | -0.38 (-0.39 to -0.37) | <0.001 |  | -1.08 (-1.12 to -1.05) | <0.001 |  | -1.17 (-1.20 to -1.13) | <0.001 |
| Western Sub-Saharan Africa | -0.49 (-0.50 to -0.47) | <0.001 |  | -0.64 (-0.65 to -0.63) | <0.001 |  | -1.61 (-1.63 to -1.58) | <0.001 |  | -1.66 (-1.68 to -1.64) | <0.001 |

AAPC average annual percentage change, DALYs disability-adjusted life-years, SDI socio-demographic index

Table S3 Main risk factors for age-standardized DALYs and mortality of total burden related to hepatitis B among people aged ≥65 years from 1990 to 2021

| Risk factors by SDI | DALYs | | | |  | Mortality | | | |
| --- | --- | --- | --- | --- | --- | --- | --- | --- | --- |
|  | Age-standardized rate in 1990 (per 100 000) | Age-standardized rate in 2021 (per 100 000) | AAPC (95% CI) | P value |  | Age-standardized rate in 1990 (per 100 000) | Age-standardized rate in 2021 (per 100 000) | AAPC (95% CI) | P value |
| Alcohol use |  |  |  |  |  |  |  |  |  |
| Global | 102.72  (59.34-157.69) | 71.69  (38.40-115.72) | -1.20  (-1.26 to -1.12) | <0.001 |  | 5.26  (3.02-8.10) | 3.86  (2.07-6.22) | -1.05  (-1.13 to -0.97) | <0.001 |
| Low SDI | 112.74  (25.16-231.14) | 95.13  (27.60-191.05) | -0.53  (-0.55 to -0.50) | <0.001 |  | 5.92  (1.33-12.08) | 5.05  (1.45-10.06) | -0.49  (-0.51 to -0.46) | <0.001 |
| Low-middle SDI | 38.74  (12.98-75.48) | 43.68  (12.30-97.66) | 0.39  (0.35 to 0.44) | <0.001 |  | 2.05  (0.68-3.99) | 2.24  (0.64-5.02) | 0.29  (0.25 to 0.33) | <0.001 |
| Middle SDI | 162.49  (99.99-245.07) | 112.28  (64.92-174.56) | -1.21  (-1.30 to -1.12) | <0.001 |  | 8.44  (5.13-12.80) | 6.24  (3.58-9.70) | -1.00  (-1.10 to -0.90) | <0.001 |
| High-middle SDI | 145.41  (89.21-215.64) | 82.82  (47.27-125.28) | -1.83  (-1.93 to -1.71) | <0.001 |  | 7.49  (4.54-11.16) | 4.56  (2.59-6.90) | -1.64  (-1.75 to -1.52) | <0.001 |
| High SDI | 48.66  (17.81-87.86) | 26.49  (9.78-49.55) | -1.98  (-2.03 to -1.93) | <0.001 |  | 2.49  (0.89-4.54) | 1.39  (0.52-2.61) | -1.90  (-1.96 to -1.83) | <0.001 |
| Drug use |  |  |  |  |  |  |  |  |  |
| Global | 5.64  (3.11-9.52) | 9.58  (6.01-14.45) | 1.72  (1.70 to 1.74) | <0.001 |  | 0.28  (0.15-0.48) | 0.50  (0.31-0.76) | 1.88  (1.86 to 1.90) | <0.001 |
| Low SDI | 5.51  (2.11-11.02) | 8.76  (3.60-17.04) | 1.50  (1.45 to 1.57) | <0.001 |  | 0.28  (0.11-0.57) | 0.47  (0.19-0.93) | 1.72  (1.63 to 1.79) | <0.001 |
| Low-middle SDI | 6.22  (2.79-12.26) | 10.24  (5.08-18.21) | 1.61  (1.53 to 1.69) | <0.001 |  | 0.31  (0.14-0.62) | 0.54  (0.27-0.97) | 1.78  (1.71 to 1.86) | <0.001 |
| Middle SDI | 5.04  (2.47-9.20) | 8.71  (4.41-14.33) | 1.75  (1.71 to 1.80) | <0.001 |  | 0.26  (0.13-0.47) | 0.47  (0.24-0.78) | 1.95  (1.92 to 2.00) | <0.001 |
| High-middle SDI | 5.59  (2.69-10.37) | 7.26  (4.13-11.68) | 0.85  (0.80 to 0.91) | <0.001 |  | 0.28  (0.13-0.51) | 0.37  (0.21-0.60) | 0.98  (0.94 to 1.04) | <0.001 |
| High SDI | 5.99  (3.10-10.22) | 12.86  (7.98-19.22) | 2.48  (2.44 to 2.52) | <0.001 |  | 0.29  (0.15-0.51) | 0.65  (0.40-0.98) | 2.59  (2.55 to 2.63) | <0.001 |
| High body-mass index |  |  |  |  |  |  |  |  |  |
| Global | 5.92  (2.40-10.39) | 14.19  (5.57-24.92) | 2.89  (2.85 to 2.92) | <0.001 |  | 0.30  (0.12-0.53) | 0.74  (0.29-1.31) | 2.97  (2.93 to 3.01) | <0.001 |
| Low SDI | 6.04  (1.98-11.72) | 8.44  (3.00-16.10) | 1.07  (1.04 to 1.09) | <0.001 |  | 0.31  (0.10-0.60) | 0.43  (0.16-0.83) | 1.12  (1.09 to 1.14) | <0.001 |
| Low-middle SDI | 3.99  (1.44-7.97) | 8.10  (2.94-15.59) | 2.33  (2.28 to 2.39) | <0.001 |  | 0.20  (0.07-0.41) | 0.41  (0.15-0.79) | 2.32  (2.28 to 2.37) | <0.001 |
| Middle SDI | 6.34  (2.63-10.77) | 17.02  (6.60-29.59) | 3.26  (3.20 to 3.32) | <0.001 |  | 0.33  (0.13-0.56) | 0.90  (0.35-1.56) | 3.34  (3.27 to 3.42) | <0.001 |
| High-middle SDI | 7.44  (2.89-13.13) | 17.85  (6.67-31.68) | 2.88  (2.83 to 2.93) | <0.001 |  | 0.38  (0.15-0.67) | 0.92  (0.35-1.65) | 2.93  (2.85 to 3.00) | <0.001 |
| High SDI | 5.18  (2.06-9.51) | 12.12  (4.80-21.78) | 2.78  (2.71 to 2.85) | <0.001 |  | 0.27  (0.10-0.49) | 0.64  (0.25-1.15) | 2.82  (2.73 to 2.91) | <0.001 |
| Smoking |  |  |  |  |  |  |  |  |  |
| Global | 26.14  (8.84-44.48) | 22.28  (7.27-39.55) | -0.48  (-0.56 to -0.42) | <0.001 |  | 1.28  (0.43-2.19) | 1.15  (0.37-2.05) | -0.30  (-0.37 to -0.24) | <0.001 |
| Low SDI | 15.13  (4.29-30.10) | 9.82  (2.78-18.93) | -1.42  (-1.47 to -1.38) | <0.001 |  | 0.79  (0.22-1.58) | 0.52  (0.14-1.00) | -1.39  (-1.44 to -1.34) | <0.001 |
| Low-middle SDI | 11.43  (3.68-20.87) | 9.75  (3.00-18.01) | -0.55  (-0.59 to -0.50) | <0.001 |  | 0.57  (0.18-1.04) | 0.50  (0.15-0.92) | -0.45  (-0.50 to -0.39) | <0.001 |
| Middle SDI | 46.77  (15.98-78.99) | 33.99  (11.29-61.69) | -1.00  (-1.07 to -0.93) | <0.001 |  | 2.33  (0.79-3.95) | 1.78  (0.59-3.23) | -0.87  (-0.95 to -0.78) | <0.001 |
| High-middle SDI | 29.93  (9.81-52.33) | 28.46  (9.03-52.54) | -0.12  (-0.22 to -0.03) | 0.005 |  | 1.45  (0.47-2.54) | 1.45  (0.46-2.68) | 0.08  (-0.01 to 0.16) | 0.072 |
| High SDI | 15.20  (4.76-27.78) | 12.34  (3.67-22.84) | -0.75  (-0.82 to -0.68) | <0.001 |  | 0.75  (0.23-1.37) | 0.65  (0.19-1.22) | -0.48  (-0.55 to -0.40) | <0.001 |

# Table S4 Age-standardized prevalence and mortality of total burden related to hepatitis B in adults aged ≥65 years between 1990 and 2021 at the global and regional levels

| Characteristics | Prevalence | | | | |  | Mortality | | | | |
| --- | --- | --- | --- | --- | --- | --- | --- | --- | --- | --- | --- |
|  | Cases in 1990 | Age-standardized rate in 1990 (per 100 000) | Cases in 2021 | Age-standardized rate in 2021 (per 100 000) | Cases  change (%) |  | Cases in 1990 | Age-standardized rate in 1990 (per 100 000) | Cases in 2021 | Age-standardized rate in 2021 (per 100 000) | Cases  change (%) |
| Global | 13331489 (11959399-14878002) | 3971.14 (3557.20-4439.45) | 27671315 (24898540-30695110) | 3553.17 (3194.82-3945.23) | 107.56 |  | 161608 (130988-199163) | 50.12 (40.44-62.04) | 255012 (204039-310534) | 33.53 (26.82-40.84) | 57.80 |
| Sex |  |  |  |  |  |  |  |  |  |  |  |
| Female | 6166617 (5478100-6945500) | 3269.91 (2902.35-3686.57) | 12309661 (10957507-13737336) | 2920.83 (2599.69-3259.99) | 99.62 |  | 65816 (50970-83043) | 35.81 (27.61-45.37) | 86866 (66331-110119) | 20.72 (15.83-26.26) | 31.98 |
| Male | 7164873 (6460063-7952483) | 4888.46 (4398.17-5441.43) | 15361654 (13877251-16968935) | 4300.04 (3879.73-4759.46) | 114.40 |  | 95792 (77839-117648) | 69.75 (56.37-86.16) | 168145 (134558-205705) | 49.61 (39.69-60.73) | 75.53 |
| Age |  |  |  |  |  |  |  |  |  |  |  |
| 65 to 69 | 5836037 (5269711-6454631) | 4721.35 (4263.19-5221.79) | 11314608 (10268896-12415872) | 4101.84 (3722.74-4501.08) | 93.87 |  | 54951 (44627-67184) | 44.46 (36.10-54.35) | 76478 (61115-93096) | 27.73 (22.16-33.75) | 39.18 |
| 70 to 74 | 3731430 (3347148-4158232) | 4407.49 (3953.58-4911.62) | 7597566 (6835903-8403272) | 3691.02 (3320.99-4082.44) | 103.61 |  | 43362 (35528-53038) | 51.22 (41.96-62.65) | 62826 (50069-76781) | 30.52 (24.32-37.30) | 44.89 |
| 75 to 79 | 2233296 (1995785-2509267) | 3628.10 (3242.25-4076.43) | 4533856 (4071082-5061274) | 3437.75 (3086.86-3837.66) | 103.01 |  | 32714 (26973-39874) | 53.15 (43.82-64.78) | 48974 (40044-58930) | 37.13 (30.36-44.68) | 49.71 |
| 80 to 84 | 1063311 (943184-1212767) | 3005.75 (2666.17-3428.23) | 2611937 (2328357-2943293) | 2982.24 (2658.46-3360.57) | 145.64 |  | 18878 (14972-23981) | 53.36 (42.32-67.79) | 36395 (29038-44386) | 41.55 (33.15-50.68) | 92.79 |
| 85 to 89 | 369855 (321892-427672) | 2447.57 (2130.17-2830.19) | 1162597 (1014725-1338197) | 2542.76 (2219.35-2926.83) | 214.34 |  | 8936 (6926-11264) | 59.14 (45.83-74.54) | 21034 (16754-25455) | 46.00 (36.64-55.67) | 135.38 |
| 90 to 94 | 82101 (69044-96721) | 1915.92 (1611.21-2257.11) | 361374 (306555-425257) | 2020.05 (1713.62-2377.15) | 340.16 |  | 2264 (1649-3055) | 52.84 (38.49-71.28) | 7373 (5698-9254) | 41.21 (31.85-51.73) | 225.59 |
| 95 plus | 15460 (12636-18711) | 1518.51 (1241.11-1837.86) | 89378 (73021-107944) | 1639.87 (1339.76-1980.51) | 478.13 |  | 503 (313-767) | 49.37 (30.79-75.30) | 1932 (1320-2632) | 35.45 (24.22-48.30) | 284.38 |
| SDI |  |  |  |  |  |  |  |  |  |  |  |
| Low SDI | 1252691 (1120821-1396639) | 7439.37 (6633.10-8336.32) | 2280310 (2041941-2540732) | 6011.32 (5365.08-6724.36) | 82.03 |  | 18002 (13317-23606) | 115.74 (84.60-153.46) | 24950 (18713-31931) | 71.83 (53.74-91.89) | 38.60 |
| Low-middle SDI | 1969860 (1763936-2205482) | 4328.62 (3864.58-4863.82) | 3997253 (3591100-4434060) | 3422.86 (3068.87-3806.12) | 102.92 |  | 35419 (24713-49117) | 87.15 (60.25-121.10) | 55424 (39838-73300) | 50.71 (36.33-67.11) | 56.48 |
| Middle SDI | 5116755 (4561121-5720380) | 6351.61 (5647.90-7128.45) | 11116482 (9954315-12385868) | 4748.92 (4245.76-5303.69) | 117.26 |  | 57576 (48329-68939) | 77.62 (64.44-94.26) | 97930 (79051-118591) | 44.38 (35.78-53.85) | 70.09 |
| High-middle SDI | 3464199 (3099002-3881565) | 4062.76 (3627.90-4560.04) | 7202737 (6451195-8027689) | 3880.21 (3472.89-4328.08) | 107.92 |  | 34097 (28589-40403) | 41.26 (34.50-49.02) | 51320 (41221-62344) | 28.30 (22.74-34.40) | 50.51 |
| High SDI | 1519218 (1380791-1672901) | 1457.69 (1324.53-1605.60) | 3060742 (2780013-3371533) | 1516.47 (1380.53-1667.34) | 101.47 |  | 16401 (13074-20218) | 15.72 (12.52-19.39) | 25234 (19491-31852) | 12.12 (9.40-15.26) | 53.85 |
| Region |  |  |  |  |  |  |  |  |  |  |  |
| Andean Latin America | 25019 (21730-29265) | 1555.00 (1347.59-1821.77) | 70105 (61819-80079) | 1394.69 (1229.06-1594.13) | 180.21 |  | 441 (261-711) | 27.88 (16.52-44.99) | 1021 (599-1649) | 20.50 (12.03-33.09) | 131.75 |
| Australasia | 52832 (45234-61210) | 2354.62 (2013.85-2730.97) | 113121 (97671-130017) | 2188.69 (1892.80-2512.07) | 114.11 |  | 160 (102-225) | 7.12 (4.52-10.04) | 373 (246-528) | 7.12 (4.70-10.05) | 133.82 |
| Caribbean | 27192 (23699-31622) | 1190.04 (1035.26-1386.33) | 49055 (42422-56392) | 1035.53 (895.86-1189.85) | 80.41 |  | 377 (216-612) | 16.98 (9.73-27.63) | 505 (291-831) | 10.62 (6.12-17.44) | 34.08 |
| Central Asia | 107306 (93828-121153) | 3056.63 (2670.64-3453.77) | 184883 (162958-209304) | 2987.14 (2630.64-3388.18) | 72.30 |  | 1054 (677-1557) | 30.22 (19.36-44.73) | 1524 (933-2383) | 25.79 (15.76-40.44) | 44.63 |
| Central Europe | 238449 (214931-267770) | 1770.19 (1592.51-1991.83) | 276575 (247270-311872) | 1237.13 (1105.80-1395.15) | 15.99 |  | 3229 (2113-4580) | 24.16 (15.83-34.24) | 3797 (2414-5577) | 17.00 (10.81-24.96) | 17.57 |
| Central Latin America | 111112 (97155-125602) | 1718.02 (1500.11-1945.17) | 300159 (262289-345626) | 1410.05 (1231.15-1624.78) | 170.14 |  | 620 (432-877) | 9.83 (6.85-13.90) | 1404 (989-1975) | 6.67 (4.70-9.38) | 126.38 |
| Central Sub-Saharan Africa | 231560 (202382-263455) | 14799.03 (12885.09-16980.09) | 458547 (403273-518420) | 12802.07 (11215.17-14541.89) | 98.02 |  | 1427 (861-2198) | 96.50 (57.96-148.78) | 2233 (1299-3477) | 64.74 (37.61-100.90) | 56.41 |
| East Asia | 6210982 (5513022-6997113) | 8890.45 (7860.10-10064.85) | 13507835 (12037457-15050540) | 6553.71 (5829.32-7320.12) | 117.48 |  | 67891 (58373-78656) | 108.23 (92.74-125.87) | 102554 (82705-126029) | 53.47 (43.11-65.73) | 51.06 |
| Eastern Europe | 352388 (309255-401167) | 1499.45 (1314.03-1709.05) | 391724 (345285-443613) | 1165.52 (1027.19-1320.55) | 11.16 |  | 1282 (954-1705) | 5.42 (4.02-7.22) | 2001 (1497-2653) | 5.95 (4.46-7.87) | 56.02 |
| Eastern Sub-Saharan Africa | 389008 (344608-438288) | 6965.58 (6146.92-7890.85) | 702584 (621325-787501) | 5751.33 (5069.73-6474.99) | 80.61 |  | 7419 (5582-9442) | 143.22 (106.97-183.56) | 11283 (8356-14522) | 100.46 (73.56-129.79) | 52.10 |
| High-income Asia Pacific | 548275 (499449-601425) | 3128.19 (2844.05-3436.91) | 1223680 (1112192-1351369) | 2728.15 (2492.23-2996.69) | 123.19 |  | 7039 (5518-8633) | 40.60 (31.74-49.91) | 10148 (7494-13206) | 20.90 (15.54-27.13) | 44.15 |
| High-income North America | 210369 (186746-238407) | 612.22 (543.43-693.84) | 390525 (347879-442939) | 609.55 (543.13-691.16) | 85.64 |  | 1219 (933-1583) | 3.54 (2.71-4.60) | 3031 (2351-3833) | 4.73 (3.67-5.98) | 148.67 |
| North Africa and Middle East | 517717 (473263-564589) | 4082.29 (3723.46-4465.73) | 1083801 (998021-1176015) | 3124.21 (2871.23-3396.65) | 109.34 |  | 18511 (12283-26005) | 169.65 (111.29-240.13) | 21750 (14621-30047) | 67.77 (45.41-93.69) | 17.50 |
| Oceania | 19671 (17404-21999) | 9578.34 (8431.63-10777.37) | 43705 (38960-49455) | 8696.52 (7728.36-9869.06) | 122.18 |  | 75 (48-120) | 39.31 (25.41-62.60) | 126 (85-180) | 26.61 (18.07-37.84) | 68.74 |
| South Asia | 1351857 (1205251-1524330) | 3277.56 (2912.48-3710.76) | 3367620 (3022757-3736482) | 2768.36 (2480.41-3079.97) | 149.11 |  | 17583 (12319-25607) | 46.45 (32.26-68.08) | 41592 (28715-55348) | 37.18 (25.49-49.61) | 136.54 |
| Southeast Asia | 1136987 (1024681-1252686) | 6028.49 (5417.98-6670.42) | 2637082 (2376914-2911384) | 5070.27 (4558.66-5615.93) | 131.94 |  | 15655 (10594-23359) | 89.64 (59.87-135.07) | 28889 (20813-38322) | 60.14 (43.19-79.79) | 84.53 |
| Southern Latin America | 21866 (19281-24598) | 524.96 (462.15-591.77) | 44788 (39134-50754) | 554.53 (484.56-628.12) | 104.83 |  | 637 (340-1063) | 15.55 (8.30-25.97) | 816 (468-1286) | 10.05 (5.76-15.85) | 28.00 |
| Southern Sub-Saharan Africa | 102851 (90684-117028) | 4735.35 (4168.65-5398.35) | 151517 (133405-172885) | 3254.17 (2859.36-3723.07) | 47.32 |  | 857 (568-1263) | 42.47 (28.09-62.82) | 1523 (1152-1974) | 35.61 (26.98-46.12) | 77.71 |
| Tropical Latin America | 170397 (149556-194588) | 2355.20 (2060.32-2699.23) | 334262 (292730-381653) | 1495.53 (1308.73-1709.24) | 96.17 |  | 1219 (941-1529) | 17.48 (13.45-21.95) | 2399 (1853-3060) | 10.79 (8.33-13.76) | 96.78 |
| Western Europe | 653966 (578821-739214) | 1175.64 (1040.77-1328.68) | 928612 (820668-1049713) | 1046.83 (928.51-1180.10) | 42.00 |  | 4504 (3117-6337) | 8.02 (5.54-11.30) | 5287 (3592-7461) | 5.72 (3.89-8.06) | 17.39 |
| Western Sub-Saharan Africa | 851687 (761646-948667) | 12370.09 (11031.71-13824.29) | 1411136 (1262789-1572135) | 10106.25 (9018.57-11299.82) | 65.69 |  | 10408 (7503-14016) | 162.51 (116.80-218.90) | 12755 (9703-16073) | 99.23 (75.68-124.72) | 22.56 |

SDI socio-demographic index

Table S5 Age-standardized incidence and DALYs of total burden related to hepatitis B in adults aged ≥65 years between 1990 and 2021 in 204 countries and territories

| location | Incidence | | | | |  | DALYs (Disability-Adjusted Life Years) | | | | |
| --- | --- | --- | --- | --- | --- | --- | --- | --- | --- | --- | --- |
|  | Cases in 1990 | Age standardized rate in 1990 (per 100 000) | Cases in 2021 | Age standardized rate in 2021 (per 100 000) | Cases change (%) |  | Cases in 1990 | Age standardized rate in 1990 (per 100 000) | Cases in 2021 | Age standardized rate in 2021 (per 100 000) | Cases change (%) |
| Afghanistan | 6886 (4243-11092) | 1254.07 (766.67-2023.13) | 7257 (4535-10924) | 1156.98 (718.04-1748.57) | 5.38 |  | 23475 (13081-39228) | 4230.49 (2319.74-7182.72) | 14149 (8479-22472) | 2245.63 (1338.07-3588.68) | -39.73 |
| Albania | 751 (450-1193) | 465.40 (277.72-740.96) | 1847 (1140-2879) | 440.90 (271.70-688.22) | 145.95 |  | 1310 (855-1877) | 805.29 (524.93-1155.60) | 1434 (843-2304) | 341.86 (200.83-549.48) | 9.47 |
| Algeria | 9508 (6058-14594) | 938.81 (593.02-1447.66) | 26081 (16756-38921) | 912.16 (582.91-1365.67) | 174.31 |  | 8058 (4369-13923) | 917.89 (504.67-1560.37) | 14600 (8428-23632) | 561.50 (318.81-914.30) | 81.19 |
| American Samoa | 18 (8-31) | 1136.32 (522.52-1971.17) | 39 (20-67) | 1058.65 (541.80-1800.68) | 116.18 |  | 10 (7-15) | 628.72 (422.82-895.54) | 23 (16-33) | 612.40 (417.08-863.75) | 121.81 |
| Andorra | 11 (5-20) | 207.78 (90.71-383.52) | 25 (11-44) | 184.56 (82.66-324.62) | 127.97 |  | 14 (8-23) | 258.73 (144.26-433.41) | 25 (13-42) | 180.11 (97.56-308.77) | 79.35 |
| Angola | 4516 (1594-8883) | 1768.70 (594.48-3514.77) | 12515 (4841-23389) | 1612.39 (620.71-3059.79) | 177.15 |  | 7933 (4719-12326) | 3007.45 (1782.58-4702.54) | 11841 (6590-18882) | 1494.61 (833.75-2386.07) | 49.26 |
| Antigua and Barbuda | 9 (4-17) | 178.84 (73.02-333.93) | 16 (7-29) | 174.31 (73.54-321.03) | 73.02 |  | 16 (10-25) | 315.82 (188.79-494.39) | 18 (11-28) | 200.76 (121.09-313.12) | 12.94 |
| Argentina | 4953 (3020-8082) | 169.36 (102.95-276.19) | 8947 (5370-14069) | 170.67 (102.47-268.26) | 80.64 |  | 7548 (3951-12578) | 255.48 (133.81-426.26) | 8623 (4726-13996) | 165.26 (90.57-268.10) | 14.24 |
| Armenia | 1091 (616-1759) | 588.71 (331.34-949.41) | 2270 (1363-3550) | 566.76 (339.20-885.78) | 108.15 |  | 792 (514-1129) | 423.56 (274.58-604.52) | 1279 (801-1940) | 322.21 (201.72-488.56) | 61.57 |
| Australia | 6640 (2584-12330) | 354.06 (137.24-659.21) | 15078 (6142-29225) | 347.87 (142.51-670.90) | 127.08 |  | 2780 (1698-3979) | 146.66 (89.54-209.99) | 6025 (3850-8595) | 139.54 (89.29-198.63) | 116.74 |
| Austria | 1374 (495-2706) | 119.58 (43.45-234.21) | 1916 (744-3684) | 115.57 (46.12-219.15) | 39.47 |  | 1694 (918-2884) | 147.65 (79.52-251.53) | 1518 (901-2369) | 92.45 (55.01-143.68) | -10.42 |
| Azerbaijan | 3272 (2032-5121) | 946.06 (586.46-1481.40) | 6868 (4337-10397) | 968.76 (610.55-1473.90) | 109.91 |  | 2185 (1294-3433) | 638.28 (376.88-1006.52) | 4201 (2366-6987) | 591.00 (329.85-989.58) | 92.29 |
| Bahamas | 20 (7-39) | 157.79 (57.41-312.44) | 46 (19-88) | 142.47 (58.64-272.49) | 136.30 |  | 39 (23-61) | 308.14 (183.49-483.91) | 62 (37-99) | 190.20 (113.61-303.34) | 60.71 |
| Bahrain | 128 (82-198) | 1175.17 (753.28-1823.06) | 653 (418-1001) | 1152.18 (739.65-1762.70) | 411.84 |  | 254 (164-365) | 2528.55 (1613.82-3665.31) | 485 (296-741) | 1048.28 (638.57-1611.05) | 91.08 |
| Bangladesh | 39572 (23028-65459) | 1125.89 (651.68-1863.91) | 122304 (76015-191281) | 1048.50 (650.23-1641.50) | 209.07 |  | 63961 (38674-95294) | 1829.14 (1104.03-2727.09) | 98436 (56940-154911) | 896.04 (515.65-1409.98) | 53.90 |
| Barbados | 34 (13-65) | 115.18 (43.43-223.76) | 56 (21-108) | 112.45 (42.58-218.31) | 65.73 |  | 57 (35-88) | 195.84 (119.48-302.66) | 58 (35-92) | 117.95 (70.53-185.51) | 2.51 |
| Belarus | 1758 (602-3740) | 159.42 (54.74-339.07) | 1766 (673-3704) | 117.85 (44.62-247.94) | 0.46 |  | 1023 (650-1499) | 92.37 (58.66-135.51) | 1442 (876-2249) | 95.20 (57.92-148.14) | 41.00 |
| Belgium | 1728 (688-3168) | 117.07 (46.73-214.04) | 2509 (1094-4575) | 114.62 (51.03-206.91) | 45.24 |  | 1701 (1034-2602) | 114.98 (69.68-176.02) | 1904 (1169-2902) | 88.27 (54.40-134.18) | 11.94 |
| Belize | 17 (6-33) | 211.83 (77.10-419.30) | 44 (17-82) | 194.93 (75.68-359.86) | 162.83 |  | 23 (13-37) | 291.78 (164.12-469.69) | 51 (29-82) | 225.73 (128.67-359.94) | 120.54 |
| Benin | 2831 (1468-5003) | 1730.87 (894.23-3059.22) | 6121 (3569-9870) | 1627.67 (945.33-2628.90) | 116.25 |  | 5247 (3369-7573) | 3186.79 (2042.00-4610.53) | 6813 (4463-9759) | 1810.84 (1187.26-2592.45) | 29.84 |
| Bermuda | 8 (3-15) | 145.89 (62.11-270.29) | 17 (8-31) | 132.23 (59.30-236.40) | 115.35 |  | 13 (8-20) | 240.30 (142.86-365.58) | 11 (7-17) | 82.65 (49.95-130.80) | -17.61 |
| Bhutan | 118 (50-215) | 701.24 (290.36-1298.99) | 328 (159-564) | 647.97 (311.77-1119.56) | 176.41 |  | 210 (113-381) | 1245.42 (671.41-2233.63) | 505 (284-866) | 1010.92 (568.03-1728.82) | 140.34 |
| Bolivia (Plurinational State of) | 987 (661-1475) | 400.33 (268.56-597.51) | 2987 (1971-4384) | 396.69 (262.29-582.97) | 202.71 |  | 1761 (783-3253) | 695.43 (310.71-1281.22) | 3865 (1925-6951) | 505.73 (252.08-908.85) | 119.51 |
| Bosnia and Herzegovina | 1040 (544-1731) | 351.11 (180.96-587.76) | 1974 (1157-3111) | 323.97 (189.39-511.75) | 89.74 |  | 1786 (1135-2570) | 585.07 (371.26-842.86) | 2204 (1309-3323) | 360.33 (214.08-543.03) | 23.39 |
| Botswana | 420 (164-813) | 943.78 (361.96-1853.44) | 853 (397-1533) | 783.19 (357.57-1425.78) | 103.25 |  | 573 (314-1009) | 1336.57 (730.91-2351.59) | 726 (421-1141) | 681.23 (396.74-1071.73) | 26.68 |
| Brazil | 35416 (18178-60326) | 498.19 (253.53-851.00) | 48419 (21460-86778) | 220.67 (97.20-396.35) | 36.71 |  | 23411 (18135-29336) | 326.57 (252.53-409.34) | 44980 (35075-57254) | 203.75 (158.77-259.25) | 92.13 |
| Brunei Darussalam | 57 (26-98) | 768.91 (356.20-1344.10) | 183 (85-322) | 712.91 (325.98-1273.55) | 222.78 |  | 97 (62-138) | 1289.40 (829.30-1851.76) | 165 (108-241) | 643.95 (418.19-944.41) | 71.16 |
| Bulgaria | 4685 (1707-9309) | 396.52 (142.88-794.08) | 5053 (1764-9941) | 343.68 (119.85-674.48) | 7.84 |  | 6258 (3989-8857) | 518.56 (331.13-735.72) | 5549 (3235-8666) | 382.08 (222.57-597.31) | -11.33 |
| Burkina Faso | 7971 (3873-14000) | 2308.97 (1094.20-4082.10) | 14660 (7763-23807) | 2082.20 (1085.53-3402.49) | 83.91 |  | 11734 (7444-18159) | 3378.56 (2147.00-5203.99) | 15291 (9565-22955) | 2170.55 (1366.48-3236.36) | 30.32 |
| Burundi | 1207 (594-2100) | 651.44 (315.75-1141.03) | 2298 (1179-3909) | 682.10 (346.19-1172.40) | 90.38 |  | 4916 (2879-7751) | 2621.61 (1527.62-4135.78) | 5710 (3311-8831) | 1700.41 (978.17-2634.81) | 16.15 |
| Cabo Verde | 254 (103-480) | 1219.30 (498.36-2292.48) | 344 (153-620) | 1016.88 (452.92-1838.10) | 35.13 |  | 317 (199-521) | 1502.14 (949.08-2462.93) | 468 (264-693) | 1387.67 (784.29-2055.12) | 47.48 |
| Cambodia | 2937 (1059-5681) | 874.45 (307.91-1709.44) | 7041 (2815-13965) | 727.23 (286.87-1460.45) | 139.75 |  | 11933 (6602-21008) | 3562.29 (1950.02-6409.45) | 18096 (10298-29721) | 1917.39 (1083.56-3166.58) | 51.65 |
| Cameroon | 5123 (2515-8755) | 1610.29 (777.76-2773.55) | 13610 (7412-22425) | 1540.78 (827.58-2551.70) | 165.67 |  | 7981 (5009-11422) | 2498.84 (1558.54-3583.34) | 14032 (7754-21629) | 1587.41 (875.81-2453.10) | 75.82 |
| Canada | 8209 (3368-15043) | 265.37 (108.23-487.50) | 19170 (7496-34754) | 274.69 (108.21-496.27) | 133.54 |  | 2096 (1222-3408) | 67.41 (39.29-109.61) | 4091 (2470-6296) | 58.63 (35.40-90.14) | 95.16 |
| Central African Republic | 941 (359-1797) | 1205.27 (432.30-2348.97) | 1490 (577-2908) | 1108.25 (419.22-2204.82) | 58.27 |  | 2021 (1221-3133) | 2469.36 (1480.58-3858.17) | 2623 (1544-4027) | 1879.84 (1092.83-2918.35) | 29.74 |
| Chad | 4365 (1821-8015) | 1780.45 (728.67-3293.01) | 7208 (3108-13497) | 1714.11 (727.27-3243.43) | 65.11 |  | 8144 (5066-13085) | 3319.22 (2053.47-5344.92) | 12256 (7606-19069) | 2903.42 (1800.96-4526.79) | 50.48 |
| Chile | 1681 (932-2715) | 198.09 (109.19-320.66) | 4579 (2545-7299) | 197.25 (109.67-314.23) | 172.44 |  | 4002 (2156-6551) | 463.15 (249.53-758.69) | 6147 (3628-9546) | 265.12 (156.45-411.73) | 53.58 |
| China | 1020157 (502738-1753765) | 1499.42 (719.43-2601.53) | 1744490 (801330-3116302) | 866.80 (393.79-1561.38) | 71.00 |  | 1288984 (1104053-1495638) | 1933.08 (1654.28-2246.22) | 1815239 (1458906-2231471) | 923.99 (743.10-1136.12) | 40.83 |
| Colombia | 5763 (2286-11537) | 422.63 (166.05-849.08) | 16815 (5807-36256) | 348.71 (120.58-751.78) | 191.77 |  | 1620 (1029-2399) | 118.48 (75.29-175.65) | 4035 (2484-6202) | 84.24 (51.89-129.44) | 149.12 |
| Comoros | 111 (42-236) | 740.98 (271.49-1584.43) | 257 (113-477) | 679.92 (296.34-1270.33) | 131.28 |  | 310 (191-462) | 2058.78 (1254.68-3073.45) | 557 (318-853) | 1482.67 (840.23-2272.83) | 79.70 |
| Congo | 964 (369-1914) | 1183.71 (440.16-2381.57) | 1845 (764-3404) | 1030.83 (417.38-1923.85) | 91.41 |  | 1802 (1091-2690) | 2159.82 (1301.64-3244.20) | 2290 (1293-3753) | 1255.12 (709.28-2051.52) | 27.02 |
| Cook Islands | 8 (4-15) | 835.20 (380.88-1502.22) | 16 (8-28) | 695.88 (338.36-1216.23) | 95.85 |  | 5 (3-8) | 512.87 (294.41-811.36) | 10 (5-16) | 410.49 (219.86-686.88) | 83.42 |
| Costa Rica | 224 (86-429) | 155.31 (59.49-297.65) | 645 (261-1259) | 134.20 (54.31-262.07) | 187.38 |  | 229 (141-343) | 158.06 (97.50-237.34) | 698 (423-1078) | 145.78 (88.23-224.93) | 205.27 |
| Coted'Ivoire | 4536 (2037-7849) | 1771.34 (771.87-3109.53) | 11080 (5171-19215) | 1478.04 (672.16-2594.64) | 144.27 |  | 6413 (4281-9288) | 2488.18 (1657.63-3586.18) | 10400 (6340-15895) | 1391.90 (843.89-2116.55) | 62.15 |
| Croatia | 1945 (983-3315) | 374.73 (186.87-638.09) | 3549 (1996-5784) | 399.00 (224.82-650.66) | 82.45 |  | 3048 (1787-4613) | 577.28 (339.12-874.63) | 3018 (1854-4545) | 342.58 (210.46-515.77) | -0.98 |
| Cuba | 1105 (397-2343) | 118.39 (42.37-251.43) | 1678 (573-3433) | 96.11 (33.11-195.79) | 51.86 |  | 1758 (1076-2697) | 188.25 (115.12-288.97) | 1977 (1156-3121) | 113.89 (66.44-179.54) | 12.47 |
| Cyprus | 262 (119-474) | 326.24 (144.73-594.42) | 596 (291-1006) | 299.15 (146.08-505.58) | 127.15 |  | 122 (68-197) | 167.10 (93.14-274.53) | 160 (93-260) | 81.88 (47.45-134.05) | 31.25 |
| Czechia | 2439 (1074-4506) | 185.56 (80.83-344.64) | 4052 (2075-6811) | 184.02 (94.29-309.12) | 66.13 |  | 6046 (3964-8500) | 453.10 (296.05-638.66) | 6748 (3990-10252) | 309.38 (182.43-469.55) | 11.61 |
| Democratic People's Republic of Korea | 17509 (6602-34764) | 1489.62 (547.26-2989.59) | 35397 (13203-67975) | 1312.93 (488.94-2525.29) | 102.16 |  | 19415 (12876-27999) | 1668.41 (1097.37-2448.05) | 28234 (18612-40950) | 1045.57 (687.73-1522.87) | 45.43 |
| Democratic Republic of the Congo | 21441 (8587-41245) | 1914.38 (726.67-3734.11) | 44270 (19782-81795) | 1799.51 (775.81-3378.62) | 106.48 |  | 16734 (9167-28019) | 1438.09 (781.08-2408.98) | 27760 (14460-46125) | 1094.77 (572.06-1824.47) | 65.89 |
| Denmark | 1023 (387-1973) | 128.47 (48.90-246.77) | 1524 (624-2855) | 131.43 (54.42-245.16) | 48.98 |  | 579 (347-911) | 73.67 (43.93-115.97) | 1143 (687-1736) | 100.07 (60.19-151.92) | 97.35 |
| Djibouti | 64 (29-117) | 760.07 (334.97-1409.57) | 291 (124-569) | 695.52 (292.22-1368.65) | 356.06 |  | 165 (97-267) | 1959.11 (1151.12-3158.67) | 660 (368-1035) | 1601.70 (883.16-2520.18) | 300.52 |
| Dominica | 9 (4-18) | 164.22 (66.59-314.24) | 11 (5-21) | 154.63 (62.64-287.76) | 21.62 |  | 18 (10-28) | 304.19 (180.02-486.68) | 15 (9-23) | 197.79 (117.12-316.84) | -16.84 |
| Dominican Republic | 1123 (540-1989) | 382.30 (182.36-679.04) | 2923 (1480-5046) | 346.22 (175.17-597.79) | 160.40 |  | 1306 (674-2248) | 460.13 (237.81-792.60) | 2512 (1166-4585) | 297.94 (138.30-543.89) | 92.42 |
| Ecuador | 1637 (1077-2467) | 389.97 (256.26-587.41) | 5429 (3581-8093) | 381.37 (251.40-568.68) | 231.67 |  | 2176 (1381-3253) | 516.88 (327.69-774.10) | 4782 (2871-7500) | 336.54 (201.92-528.18) | 119.73 |
| Egypt | 25705 (12671-45340) | 1379.68 (664.70-2456.71) | 46969 (23565-79158) | 1049.79 (514.00-1805.51) | 82.72 |  | 182228 (115429-253651) | 11715.50 (7315.58-16474.70) | 195301 (119086-291478) | 4689.38 (2862.21-6964.76) | 7.17 |
| El Salvador | 340 (108-708) | 137.71 (43.49-286.92) | 610 (188-1239) | 111.28 (34.75-224.89) | 79.27 |  | 397 (224-673) | 160.56 (90.46-272.81) | 583 (310-1017) | 106.58 (56.63-185.72) | 46.95 |
| Equatorial Guinea | 110 (59-189) | 797.60 (423.02-1381.50) | 262 (154-416) | 764.46 (447.04-1215.00) | 138.12 |  | 222 (106-378) | 1503.02 (715.45-2583.29) | 266 (122-466) | 751.79 (342.77-1317.77) | 20.17 |
| Eritrea | 557 (227-1069) | 897.94 (348.06-1763.12) | 1469 (609-2794) | 827.69 (337.15-1597.38) | 163.70 |  | 1806 (1117-2703) | 2859.37 (1741.79-4336.05) | 3442 (2038-5134) | 1965.02 (1150.98-2950.44) | 90.62 |
| Estonia | 383 (155-748) | 211.00 (85.42-411.31) | 421 (185-801) | 158.77 (70.59-298.96) | 10.00 |  | 178 (112-266) | 98.41 (62.02-146.88) | 283 (177-431) | 110.72 (69.25-168.05) | 58.67 |
| Eswatini | 237 (97-445) | 1123.96 (450.26-2131.24) | 384 (173-697) | 912.10 (398.77-1678.32) | 62.06 |  | 349 (201-565) | 1680.34 (958.79-2754.22) | 566 (289-963) | 1335.14 (688.69-2268.56) | 62.11 |
| Ethiopia | 16559 (7884-29016) | 1119.45 (511.23-1994.32) | 31952 (15907-54374) | 972.22 (479.22-1662.70) | 92.95 |  | 45984 (29172-63774) | 3083.06 (1949.11-4283.40) | 55518 (40878-71418) | 1696.49 (1234.35-2190.78) | 20.73 |
| Fiji | 185 (102-307) | 770.31 (417.64-1293.27) | 420 (241-687) | 725.12 (408.51-1200.75) | 127.68 |  | 137 (84-216) | 548.16 (336.33-869.16) | 265 (169-390) | 444.17 (282.56-652.45) | 93.43 |
| Finland | 853 (303-1729) | 125.40 (44.60-253.80) | 1445 (552-2773) | 114.57 (44.19-218.23) | 69.34 |  | 672 (422-1023) | 98.06 (61.57-149.37) | 1609 (974-2497) | 129.49 (78.59-200.61) | 139.50 |
| France | 12391 (6148-21173) | 158.02 (79.63-269.92) | 21276 (11652-35373) | 158.72 (87.78-262.52) | 71.71 |  | 13309 (8126-20089) | 173.79 (105.58-262.56) | 16329 (9810-24995) | 123.49 (74.39-188.14) | 22.69 |
| Gabon | 523 (226-997) | 1096.25 (465.74-2102.03) | 811 (361-1396) | 1042.47 (450.13-1820.20) | 55.07 |  | 952 (558-1450) | 1973.05 (1150.59-3021.28) | 934 (501-1502) | 1174.76 (626.74-1896.47) | -1.93 |
| Gambia | 512 (271-879) | 1922.79 (1001.25-3314.62) | 1342 (795-2070) | 1830.27 (1079.17-2834.34) | 162.35 |  | 754 (500-1079) | 2798.67 (1854.77-4004.29) | 1705 (936-2645) | 2310.70 (1261.90-3597.66) | 126.26 |
| Georgia | 1776 (851-3087) | 348.44 (166.38-607.07) | 1937 (968-3178) | 340.30 (170.61-558.09) | 9.06 |  | 2199 (1303-3407) | 429.02 (253.99-665.48) | 1497 (896-2422) | 264.46 (158.54-427.55) | -31.93 |
| Germany | 10855 (5408-18466) | 91.67 (45.90-155.32) | 18655 (9524-30575) | 103.06 (53.38-167.80) | 71.85 |  | 17184 (10134-27140) | 144.61 (84.99-228.38) | 18907 (11488-29194) | 107.84 (65.66-166.02) | 10.03 |
| Ghana | 8430 (3485-15339) | 1857.38 (745.83-3410.03) | 20674 (8782-37621) | 1667.20 (692.92-3058.30) | 145.24 |  | 11158 (7152-16511) | 2470.92 (1570.05-3681.41) | 21048 (12945-30890) | 1760.86 (1077.40-2591.49) | 88.64 |
| Greece | 6798 (3337-11875) | 481.07 (236.38-840.40) | 10034 (5023-17113) | 445.82 (227.85-753.02) | 47.60 |  | 2452 (1651-3498) | 173.59 (116.71-247.82) | 4443 (2811-6313) | 198.00 (125.36-279.20) | 81.21 |
| Greenland | 4 (1-7) | 172.65 (61.84-349.71) | 9 (3-19) | 163.20 (52.57-341.26) | 142.09 |  | 2 (1-3) | 94.67 (55.41-150.97) | 5 (3-9) | 90.98 (48.78-155.28) | 142.44 |
| Grenada | 11 (4-22) | 166.61 (61.04-332.68) | 14 (5-27) | 141.07 (52.57-279.65) | 23.81 |  | 18 (11-29) | 272.94 (160.71-435.07) | 21 (13-32) | 215.11 (131.44-331.07) | 14.85 |
| Guam | 82 (48-134) | 1526.05 (884.03-2492.01) | 276 (165-437) | 1441.27 (865.00-2284.84) | 236.96 |  | 61 (41-82) | 1150.66 (774.47-1547.57) | 118 (82-158) | 618.85 (428.33-828.87) | 92.65 |
| Guatemala | 780 (293-1593) | 298.73 (109.76-616.43) | 2190 (797-4283) | 234.77 (84.71-461.33) | 180.77 |  | 869 (529-1324) | 331.82 (202.35-508.00) | 2215 (1303-3621) | 237.13 (139.40-387.95) | 154.95 |
| Guinea | 5681 (2682-9904) | 2001.17 (932.54-3506.55) | 7945 (3788-13892) | 1832.26 (861.76-3218.86) | 39.86 |  | 9792 (6759-13804) | 3416.81 (2349.60-4831.41) | 10937 (7289-15817) | 2510.75 (1671.16-3632.51) | 11.68 |
| Guinea-Bissau | 595 (258-1082) | 1969.72 (835.36-3622.33) | 823 (351-1518) | 1694.61 (712.83-3168.73) | 38.38 |  | 1461 (941-2073) | 4707.37 (3018.55-6694.38) | 1327 (860-1899) | 2730.26 (1767.44-3921.98) | -9.17 |
| Guyana | 88 (36-167) | 291.91 (119.34-560.89) | 126 (51-236) | 243.34 (96.24-460.21) | 43.58 |  | 171 (93-288) | 573.50 (311.98-963.90) | 161 (86-278) | 305.04 (164.21-527.76) | -6.05 |
| Haiti | 802 (334-1538) | 319.70 (129.41-620.70) | 1497 (633-2732) | 285.52 (118.13-526.92) | 86.63 |  | 1614 (784-2973) | 636.34 (306.53-1184.00) | 2361 (1116-4520) | 441.19 (207.53-842.20) | 46.27 |
| Honduras | 400 (161-786) | 248.47 (97.93-490.31) | 1053 (423-2101) | 202.38 (79.66-407.08) | 162.95 |  | 321 (173-550) | 198.84 (106.65-341.62) | 1098 (556-1995) | 210.17 (106.20-381.59) | 242.13 |
| Hungary | 3996 (2455-6415) | 285.75 (175.16-459.28) | 5993 (3712-9385) | 298.78 (185.12-467.89) | 49.98 |  | 8064 (4813-12249) | 564.40 (336.42-857.80) | 7534 (4362-11722) | 377.47 (218.49-587.27) | -6.58 |
| Iceland | 27 (11-53) | 101.52 (40.44-198.32) | 53 (21-106) | 97.23 (38.89-193.56) | 94.81 |  | 17 (10-26) | 63.38 (38.78-95.87) | 44 (25-74) | 81.44 (45.52-134.81) | 162.31 |
| India | 179182 (92197-303746) | 545.99 (275.95-931.83) | 483620 (261155-793486) | 485.13 (259.68-800.31) | 169.90 |  | 215983 (156024-299391) | 647.43 (465.44-898.80) | 567809 (368904-780241) | 573.04 (370.77-789.08) | 162.90 |
| Indonesia | 77637 (42328-126953) | 1116.68 (599.11-1840.41) | 182921 (104821-287942) | 1013.66 (574.78-1610.18) | 135.61 |  | 122161 (78714-190542) | 1813.32 (1152.51-2836.91) | 239607 (171377-319853) | 1438.00 (1026.32-1921.56) | 96.14 |
| Iran (Islamic Republic of) | 18470 (11494-29040) | 942.70 (577.56-1483.42) | 37440 (22799-57501) | 618.04 (375.56-950.18) | 102.71 |  | 13538 (9672-18591) | 725.55 (512.55-1002.74) | 24078 (19395-30671) | 405.26 (325.16-518.73) | 77.85 |
| Iraq | 3296 (2205-5038) | 531.74 (355.94-811.82) | 8824 (5920-13023) | 521.08 (350.21-769.37) | 167.70 |  | 5859 (3446-9145) | 941.46 (552.96-1471.98) | 11677 (6920-17811) | 692.67 (408.84-1058.03) | 99.32 |
| Ireland | 469 (186-891) | 115.26 (45.63-219.55) | 839 (328-1583) | 112.33 (44.08-211.15) | 78.98 |  | 255 (160-384) | 62.61 (39.12-94.20) | 482 (277-758) | 64.53 (37.13-101.25) | 88.86 |
| Israel | 689 (357-1151) | 147.07 (75.76-245.74) | 1650 (891-2680) | 138.37 (74.91-224.59) | 139.38 |  | 607 (376-911) | 128.71 (79.69-193.26) | 816 (505-1228) | 68.47 (42.46-102.94) | 34.34 |
| Italy | 36220 (16051-65140) | 422.52 (187.92-757.44) | 40913 (17221-78098) | 303.84 (130.19-572.99) | 12.96 |  | 25769 (20736-31882) | 299.19 (240.54-370.59) | 16276 (12560-20530) | 116.61 (90.61-146.65) | -36.84 |
| Jamaica | 217 (77-442) | 128.82 (46.21-262.15) | 316 (107-662) | 119.38 (40.83-248.48) | 45.77 |  | 206 (122-318) | 122.34 (72.54-188.83) | 219 (130-345) | 82.97 (49.31-130.49) | 6.24 |
| Japan | 108729 (59014-175075) | 717.64 (388.26-1156.97) | 189892 (98248-315733) | 537.33 (283.79-884.81) | 74.65 |  | 52561 (43517-62950) | 347.04 (286.64-416.24) | 72406 (55714-90144) | 196.30 (153.66-242.95) | 37.76 |
| Jordan | 639 (286-1153) | 713.63 (313.69-1298.39) | 3338 (1665-5750) | 639.65 (313.48-1109.32) | 422.10 |  | 888 (541-1384) | 1029.87 (621.62-1617.00) | 1683 (1000-2634) | 339.47 (200.90-531.28) | 89.58 |
| Kazakhstan | 3564 (2086-5633) | 377.83 (221.01-597.49) | 5376 (3383-8193) | 374.80 (234.18-574.00) | 50.81 |  | 4552 (3022-6523) | 475.49 (314.84-682.39) | 8029 (4559-12994) | 555.19 (315.73-901.44) | 76.37 |
| Kenya | 8236 (4198-13772) | 1306.43 (655.48-2198.48) | 14487 (6919-24870) | 835.19 (389.93-1449.24) | 75.89 |  | 14999 (9353-25849) | 2404.37 (1499.30-4126.15) | 36490 (25499-53462) | 2187.76 (1522.72-3203.43) | 143.29 |
| Kiribati | 41 (18-75) | 1501.71 (648.08-2787.77) | 64 (29-116) | 1326.40 (590.18-2461.28) | 54.90 |  | 37 (24-57) | 1285.23 (823.93-2011.81) | 49 (31-73) | 982.19 (629.68-1482.36) | 31.13 |
| Kuwait | 143 (87-231) | 410.29 (250.30-663.52) | 669 (411-1068) | 389.31 (238.01-621.96) | 367.87 |  | 176 (117-246) | 510.11 (337.73-714.56) | 457 (267-717) | 275.74 (161.81-433.99) | 159.02 |
| Kyrgyzstan | 997 (433-1898) | 433.29 (187.30-824.38) | 1435 (667-2654) | 409.96 (188.89-762.55) | 43.92 |  | 1373 (847-2114) | 596.92 (367.13-922.74) | 1394 (818-2239) | 389.61 (228.42-626.32) | 1.53 |
| Lao People's Democratic Republic | 1085 (406-2167) | 656.72 (242.12-1339.86) | 2000 (718-4235) | 588.42 (207.34-1263.53) | 84.26 |  | 3119 (1764-5307) | 1883.57 (1042.00-3260.62) | 3529 (2043-5646) | 1053.57 (608.88-1684.94) | 13.12 |
| Latvia | 386 (133-850) | 121.64 (41.88-266.69) | 306 (125-632) | 79.68 (33.32-162.72) | -20.74 |  | 314 (205-452) | 99.49 (64.82-142.85) | 327 (199-500) | 88.02 (53.45-134.48) | 3.99 |
| Lebanon | 1711 (1133-2624) | 1022.30 (676.71-1562.33) | 5336 (3522-7997) | 978.55 (645.46-1463.92) | 211.84 |  | 1845 (1078-2872) | 1138.46 (662.00-1781.30) | 3017 (1846-4572) | 538.94 (331.49-814.99) | 63.52 |
| Lesotho | 787 (325-1535) | 1103.93 (448.22-2174.95) | 783 (347-1443) | 879.36 (379.61-1652.79) | -0.54 |  | 1084 (582-2078) | 1536.72 (814.33-2973.59) | 1326 (737-2167) | 1486.74 (829.56-2426.72) | 22.39 |
| Liberia | 2698 (1420-4689) | 2709.54 (1408.41-4735.30) | 3532 (1825-5836) | 2507.41 (1276.91-4171.35) | 30.93 |  | 3273 (2131-4769) | 3284.84 (2130.73-4769.58) | 3173 (2025-4598) | 2257.61 (1441.38-3268.26) | -3.06 |
| Libya | 1018 (557-1688) | 723.91 (395.25-1202.59) | 2368 (1408-3693) | 673.32 (399.09-1052.34) | 132.71 |  | 1793 (873-3627) | 1290.31 (623.55-2626.42) | 3336 (1865-5562) | 957.48 (531.15-1603.70) | 86.08 |
| Lithuania | 568 (190-1161) | 144.05 (48.32-293.68) | 568 (211-1185) | 104.10 (39.61-214.68) | -0.03 |  | 297 (189-443) | 75.43 (47.99-112.63) | 500 (311-748) | 95.86 (59.77-143.20) | 68.47 |
| Luxembourg | 78 (33-140) | 154.85 (65.18-279.03) | 149 (61-266) | 158.19 (65.46-280.44) | 92.20 |  | 81 (47-127) | 162.24 (94.74-257.03) | 98 (59-150) | 103.54 (62.93-158.36) | 21.04 |
| Madagascar | 3574 (1465-7236) | 924.67 (369.68-1892.34) | 6122 (2326-12170) | 847.51 (312.87-1708.66) | 71.27 |  | 7608 (4473-12605) | 1952.77 (1145.63-3230.10) | 9996 (5704-16389) | 1393.00 (795.09-2276.26) | 31.38 |
| Malawi | 2799 (1218-5128) | 948.14 (401.18-1764.96) | 4817 (2318-8614) | 858.73 (405.17-1548.50) | 72.12 |  | 8085 (5006-11598) | 2699.51 (1672.79-3879.98) | 11919 (7460-17590) | 2133.83 (1320.19-3169.52) | 47.42 |
| Malaysia | 7692 (4853-12083) | 1136.15 (715.51-1784.12) | 27153 (17814-40839) | 1152.93 (755.71-1734.92) | 253.00 |  | 7469 (5251-9977) | 1089.90 (765.84-1458.00) | 25183 (16961-34632) | 1064.09 (713.52-1468.64) | 237.16 |
| Maldives | 31 (14-60) | 532.58 (226.48-1029.47) | 100 (43-181) | 449.05 (192.86-815.37) | 220.54 |  | 79 (48-121) | 1357.18 (823.98-2107.14) | 103 (64-153) | 473.74 (295.25-700.32) | 30.23 |
| Mali | 4284 (2121-7601) | 1431.61 (697.42-2574.27) | 8379 (4192-14548) | 1289.14 (635.71-2258.20) | 95.58 |  | 9326 (6196-13092) | 3056.71 (2009.16-4319.90) | 16605 (10635-23917) | 2543.87 (1631.31-3665.52) | 78.06 |
| Malta | 35 (12-70) | 87.57 (29.27-176.89) | 87 (35-168) | 88.28 (35.20-169.31) | 150.75 |  | 39 (24-61) | 98.17 (59.69-151.26) | 66 (40-103) | 66.62 (39.95-103.58) | 67.67 |
| Marshall Islands | 15 (7-28) | 1174.31 (513.51-2178.51) | 26 (12-44) | 1085.77 (494.44-1888.52) | 72.79 |  | 15 (9-25) | 1141.17 (653.10-1895.61) | 21 (12-33) | 837.78 (482.28-1364.78) | 34.83 |
| Mauritania | 2217 (1085-3937) | 2581.41 (1242.68-4614.85) | 3954 (1505-7142) | 2293.96 (859.83-4177.75) | 78.33 |  | 4157 (2225-6967) | 4785.06 (2559.01-8040.37) | 3837 (2338-5861) | 2251.74 (1370.01-3433.57) | -7.68 |
| Mauritius | 295 (124-537) | 490.64 (202.04-900.66) | 803 (302-1469) | 477.51 (178.39-879.25) | 172.27 |  | 571 (371-772) | 952.44 (621.44-1288.41) | 556 (337-820) | 331.39 (201.55-488.33) | -2.53 |
| Mexico | 5794 (3262-9563) | 173.45 (97.06-286.80) | 10017 (5370-16662) | 94.01 (50.09-156.98) | 72.89 |  | 6500 (4609-8997) | 195.41 (138.43-270.34) | 14194 (10398-18962) | 132.81 (97.32-177.43) | 118.36 |
| Micronesia (Federated States of) | 48 (22-86) | 1182.59 (536.30-2116.48) | 55 (26-94) | 1094.97 (485.94-1910.85) | 13.80 |  | 42 (26-65) | 986.60 (606.20-1542.56) | 37 (22-56) | 707.27 (426.64-1094.06) | -12.04 |
| Monaco | 10 (4-19) | 145.31 (61.78-271.80) | 14 (5-26) | 146.09 (60.02-274.39) | 34.49 |  | 9 (5-14) | 125.05 (70.44-202.18) | 14 (8-23) | 144.77 (80.16-247.42) | 56.40 |
| Mongolia | 2221 (1405-3453) | 2580.45 (1622.17-4019.26) | 3754 (2375-5661) | 2555.16 (1613.93-3860.74) | 68.99 |  | 2091 (1301-3159) | 2359.79 (1463.28-3570.48) | 2876 (1679-4638) | 1930.98 (1129.42-3125.59) | 37.52 |
| Montenegro | 77 (35-138) | 153.20 (69.91-275.91) | 135 (65-240) | 140.77 (66.37-252.62) | 76.52 |  | 128 (80-190) | 253.34 (156.94-377.14) | 254 (158-391) | 262.72 (162.51-404.42) | 98.81 |
| Morocco | 8359 (4904-13496) | 726.68 (423.91-1175.82) | 18880 (11782-29196) | 682.79 (425.27-1055.52) | 125.86 |  | 10329 (5815-17302) | 916.07 (510.50-1543.78) | 17874 (9985-29238) | 664.17 (368.76-1087.31) | 73.05 |
| Mozambique | 8277 (4174-14379) | 1863.58 (916.72-3268.20) | 13172 (6592-22552) | 1644.05 (811.56-2851.20) | 59.14 |  | 10300 (6917-14455) | 2385.01 (1597.38-3352.62) | 14695 (8566-23884) | 1885.40 (1087.10-3073.35) | 42.68 |
| Myanmar | 7652 (4074-13258) | 432.61 (227.80-750.77) | 16470 (9264-27104) | 422.79 (236.39-697.32) | 115.25 |  | 17468 (9094-32411) | 927.00 (479.81-1731.48) | 24412 (13029-39817) | 606.56 (321.15-992.42) | 39.75 |
| Namibia | 541 (227-999) | 1022.25 (411.41-1920.75) | 948 (416-1686) | 901.51 (385.45-1627.26) | 75.13 |  | 634 (330-1197) | 1217.81 (628.49-2304.29) | 886 (509-1434) | 857.23 (493.49-1386.10) | 39.82 |
| Nauru | 4 (2-7) | 1296.87 (574.74-2345.74) | 5 (2-9) | 1188.06 (549.45-2161.55) | 18.70 |  | 4 (2-5) | 1112.01 (679.92-1665.08) | 3 (1-5) | 719.36 (340.44-1164.79) | -15.20 |
| Nepal | 5144 (3409-7928) | 776.40 (515.99-1188.42) | 13944 (9269-20437) | 725.74 (483.52-1065.08) | 171.06 |  | 7339 (3737-14073) | 1071.58 (540.13-2064.30) | 18267 (10505-29028) | 959.02 (549.72-1524.15) | 148.90 |
| Netherlands | 2536 (1504-4060) | 132.57 (78.62-212.32) | 4669 (2875-7204) | 135.45 (83.63-208.89) | 84.15 |  | 1071 (654-1632) | 56.06 (34.18-85.51) | 2250 (1380-3415) | 65.56 (40.31-99.44) | 110.21 |
| New Zealand | 1169 (470-2201) | 309.43 (124.15-583.43) | 2551 (1114-4775) | 317.25 (139.22-593.01) | 118.14 |  | 379 (295-477) | 99.70 (77.61-125.67) | 728 (570-921) | 90.37 (70.80-114.42) | 92.20 |
| Nicaragua | 156 (66-299) | 131.62 (54.98-252.39) | 442 (207-792) | 111.78 (51.90-201.23) | 182.40 |  | 206 (116-340) | 173.50 (97.74-286.94) | 493 (284-809) | 124.50 (71.73-204.68) | 139.25 |
| Niger | 3858 (1653-7564) | 1945.98 (798.87-3869.29) | 9821 (3923-17731) | 1660.82 (648.07-3024.63) | 154.59 |  | 7043 (4447-11120) | 3526.58 (2219.65-5580.00) | 12701 (7541-21774) | 2179.39 (1295.77-3708.51) | 80.34 |
| Nigeria | 73785 (36510-131684) | 2076.41 (1010.88-3724.43) | 110440 (56670-190846) | 1742.89 (878.00-3033.21) | 49.68 |  | 100458 (67647-143371) | 2850.24 (1921.61-4061.15) | 91913 (65918-124227) | 1481.51 (1074.13-1986.76) | -8.51 |
| Niue | 2 (1-4) | 967.30 (457.63-1743.85) | 2 (1-3) | 891.42 (398.59-1579.90) | -14.97 |  | 2 (1-2) | 786.51 (502.87-1136.51) | 1 (1-2) | 594.95 (354.76-917.81) | -28.79 |
| North Macedonia | 527 (275-876) | 353.10 (182.76-586.81) | 1076 (610-1679) | 342.33 (191.93-536.86) | 103.96 |  | 703 (446-1034) | 457.39 (289.87-674.71) | 1140 (693-1785) | 362.68 (219.74-570.80) | 62.12 |
| Northern Mariana Islands | 21 (13-33) | 2399.19 (1466.36-3758.27) | 84 (52-128) | 2282.81 (1420.05-3505.34) | 295.50 |  | 12 (8-17) | 1330.93 (870.34-1874.16) | 41 (28-59) | 1149.06 (775.87-1631.51) | 251.85 |
| Norway | 760 (321-1396) | 109.43 (46.58-200.53) | 833 (355-1543) | 87.07 (37.67-160.09) | 9.61 |  | 355 (277-453) | 51.38 (40.00-65.61) | 608 (467-777) | 63.23 (48.69-80.74) | 71.18 |
| Oman | 432 (203-750) | 927.10 (430.07-1622.36) | 951 (483-1623) | 803.71 (401.55-1380.43) | 120.15 |  | 635 (352-1063) | 1372.17 (755.82-2306.71) | 736 (426-1171) | 647.53 (371.00-1034.02) | 15.87 |
| Pakistan | 31838 (14841-57421) | 687.55 (313.93-1248.38) | 49295 (23270-85285) | 559.00 (259.23-975.48) | 54.83 |  | 54075 (27867-107718) | 1189.11 (607.01-2376.39) | 89686 (61115-127518) | 1049.91 (711.60-1494.52) | 65.85 |
| Palau | 9 (5-15) | 1144.07 (574.05-1940.35) | 19 (9-32) | 1104.30 (550.43-1890.61) | 104.86 |  | 8 (6-12) | 1007.23 (673.36-1426.62) | 12 (7-18) | 669.88 (408.78-997.69) | 41.11 |
| Palestine | 696 (427-1083) | 996.45 (607.78-1553.62) | 1738 (1106-2640) | 969.00 (615.77-1475.95) | 149.80 |  | 1187 (644-2181) | 1752.74 (944.09-3233.42) | 1301 (816-1923) | 757.30 (471.64-1126.38) | 9.62 |
| Panama | 136 (51-280) | 108.71 (40.80-225.15) | 336 (122-685) | 86.71 (31.63-176.59) | 147.32 |  | 182 (112-275) | 146.10 (89.59-221.49) | 344 (203-549) | 88.99 (52.59-141.82) | 89.27 |
| Papua New Guinea | 1441 (542-2918) | 1141.99 (415.13-2359.19) | 3374 (1307-6749) | 1019.35 (380.29-2070.72) | 134.16 |  | 741 (404-1365) | 571.21 (313.14-1044.77) | 1287 (739-2180) | 383.09 (220.04-647.04) | 73.64 |
| Paraguay | 611 (284-1078) | 334.14 (154.61-591.52) | 1441 (688-2472) | 296.78 (141.29-510.59) | 136.09 |  | 437 (252-669) | 237.50 (137.23-364.13) | 1267 (712-2075) | 258.48 (145.30-423.30) | 190.11 |
| Peru | 4098 (1797-7096) | 434.15 (189.16-753.56) | 12101 (6057-20179) | 425.74 (213.29-709.34) | 195.27 |  | 4219 (2351-7024) | 446.22 (248.63-742.92) | 9776 (5433-16190) | 344.44 (191.39-570.32) | 131.70 |
| Philippines | 34158 (16446-61146) | 1552.28 (732.42-2796.93) | 75930 (33865-139164) | 1198.52 (523.08-2217.75) | 122.29 |  | 18670 (12311-29605) | 877.39 (577.21-1397.71) | 43202 (33008-55243) | 678.72 (519.11-865.58) | 131.40 |
| Poland | 19579 (12744-29480) | 499.62 (324.00-753.34) | 10339 (5715-16524) | 143.67 (79.14-229.84) | -47.20 |  | 10248 (7524-13545) | 259.41 (190.28-343.05) | 13561 (10086-17948) | 186.20 (138.54-246.12) | 32.33 |
| Portugal | 2021 (765-3837) | 149.23 (55.75-284.20) | 3434 (1298-6347) | 146.36 (56.85-266.77) | 69.91 |  | 2670 (1491-4354) | 196.70 (110.00-320.67) | 2335 (1445-3519) | 100.77 (62.49-151.56) | -12.53 |
| Puerto Rico | 547 (205-1041) | 160.41 (59.73-305.78) | 965 (381-1805) | 137.11 (55.30-253.92) | 76.47 |  | 1148 (655-1859) | 339.17 (193.23-550.33) | 1126 (676-1755) | 160.78 (96.58-249.75) | -1.93 |
| Qatar | 65 (43-99) | 1369.91 (911.36-2084.23) | 508 (340-763) | 1357.45 (909.04-2028.23) | 683.21 |  | 113 (68-173) | 2562.21 (1539.40-3962.53) | 478 (289-763) | 1331.80 (804.49-2109.41) | 324.73 |
| Republic of Korea | 33468 (21821-51414) | 1492.43 (971.17-2290.40) | 137636 (91110-211501) | 1615.64 (1069.31-2482.22) | 311.24 |  | 80177 (57354-104399) | 3559.09 (2539.67-4652.85) | 90059 (61135-126434) | 1059.00 (718.09-1487.59) | 12.33 |
| Republic of Moldova | 2704 (1078-5642) | 721.20 (282.72-1518.15) | 2871 (1113-5975) | 513.55 (198.15-1073.56) | 6.17 |  | 1953 (1060-3298) | 498.15 (270.02-842.77) | 1539 (847-2570) | 271.64 (149.78-453.70) | -21.20 |
| Romania | 12945 (5069-24686) | 526.66 (205.32-1009.31) | 16866 (7177-31100) | 446.64 (190.51-823.18) | 30.29 |  | 17568 (9803-27116) | 699.51 (390.29-1080.77) | 22682 (12479-36156) | 603.29 (332.09-961.02) | 29.11 |
| Russian Federation | 35623 (15252-69815) | 238.65 (101.52-467.24) | 36439 (14988-72402) | 158.49 (64.49-316.83) | 2.29 |  | 15267 (11388-20143) | 101.21 (75.43-133.86) | 29714 (22364-38995) | 127.62 (96.24-167.04) | 94.63 |
| Rwanda | 1262 (524-2454) | 583.65 (237.58-1160.32) | 2334 (854-4732) | 492.08 (176.51-1013.74) | 84.86 |  | 5430 (3194-8200) | 2507.50 (1473.45-3798.69) | 6744 (3786-10909) | 1463.77 (822.18-2361.55) | 24.21 |
| Saint Kitts and Nevis | 8 (3-16) | 196.52 (74.26-383.82) | 11 (5-19) | 183.98 (79.69-342.74) | 30.45 |  | 17 (10-27) | 413.71 (244.33-646.91) | 15 (9-23) | 261.57 (158.31-409.57) | -12.72 |
| Saint Lucia | 17 (7-34) | 209.88 (84.55-405.53) | 39 (17-71) | 190.73 (83.17-346.70) | 125.59 |  | 29 (16-47) | 360.13 (203.12-581.82) | 34 (19-56) | 164.12 (91.56-271.66) | 16.45 |
| Saint Vincent and the Grenadines | 11 (4-22) | 162.05 (63.67-328.06) | 19 (7-40) | 151.80 (53.98-310.62) | 75.19 |  | 19 (11-27) | 271.74 (167.59-402.53) | 22 (13-35) | 172.25 (101.14-272.31) | 18.16 |
| Samoa | 87 (42-146) | 1284.15 (614.71-2192.04) | 125 (61-217) | 1114.58 (538.74-1943.86) | 44.34 |  | 59 (39-88) | 848.87 (555.63-1268.62) | 76 (50-109) | 665.93 (440.71-961.63) | 28.06 |
| San Marino | 7 (4-13) | 218.70 (110.70-380.84) | 14 (7-24) | 209.12 (106.56-352.53) | 94.23 |  | 6 (3-10) | 178.41 (101.64-291.04) | 6 (3-10) | 86.94 (47.27-145.68) | -0.02 |
| Sao Tome and Principe | 116 (58-206) | 2118.27 (1038.91-3757.20) | 159 (86-264) | 2009.37 (1075.90-3345.63) | 36.55 |  | 175 (112-252) | 3257.67 (2076.67-4701.69) | 176 (90-271) | 2270.45 (1162.38-3509.89) | 0.46 |
| Saudi Arabia | 3544 (1935-5871) | 881.03 (477.79-1464.77) | 9352 (5699-14883) | 892.82 (537.31-1423.58) | 163.90 |  | 11112 (5746-21552) | 2813.00 (1440.99-5493.37) | 11129 (6802-17396) | 1136.66 (686.95-1793.08) | 0.15 |
| Senegal | 4713 (2112-8538) | 1830.13 (806.79-3335.96) | 9657 (4261-17052) | 1625.40 (712.36-2889.08) | 104.91 |  | 6566 (4381-9456) | 2532.71 (1691.43-3645.18) | 9464 (5477-14143) | 1600.39 (922.52-2399.15) | 44.12 |
| Serbia | 2124 (1031-3733) | 220.71 (104.26-391.49) | 3610 (1822-6171) | 216.83 (109.32-370.80) | 69.97 |  | 3667 (2273-5498) | 376.69 (232.14-568.13) | 4807 (2853-7331) | 287.46 (170.49-438.48) | 31.09 |
| Seychelles | 38 (18-66) | 732.95 (338.06-1287.16) | 64 (30-113) | 692.43 (326.38-1227.85) | 68.70 |  | 67 (45-93) | 1299.07 (864.70-1804.81) | 69 (43-102) | 755.98 (473.88-1126.08) | 2.33 |
| Sierra Leone | 2788 (1215-5226) | 1609.12 (694.93-3026.53) | 3901 (1712-7337) | 1385.53 (597.65-2626.42) | 39.92 |  | 5397 (3428-8026) | 3112.93 (1973.87-4635.75) | 4817 (3061-7171) | 1715.00 (1091.26-2546.87) | -10.75 |
| Singapore | 2016 (1244-3179) | 1170.13 (718.12-1851.01) | 8469 (5189-13338) | 1094.89 (669.98-1725.29) | 320.17 |  | 1760 (1387-2164) | 999.07 (785.04-1230.88) | 4101 (3000-5295) | 536.14 (391.15-693.01) | 133.00 |
| Slovakia | 1355 (544-2473) | 242.65 (96.71-444.57) | 2177 (998-3865) | 228.79 (103.79-407.50) | 60.65 |  | 3086 (1896-4584) | 539.49 (332.01-800.80) | 3283 (1880-5206) | 340.35 (195.33-539.56) | 6.38 |
| Slovenia | 531 (254-931) | 246.56 (118.37-432.65) | 1187 (624-2044) | 277.18 (146.64-475.73) | 123.67 |  | 1197 (730-1790) | 554.73 (337.38-831.22) | 1513 (960-2237) | 356.09 (226.10-525.98) | 26.41 |
| Solomon Islands | 109 (40-233) | 1139.75 (405.42-2494.99) | 216 (75-474) | 922.65 (316.87-2048.00) | 98.24 |  | 130 (71-235) | 1346.99 (737.18-2409.76) | 238 (150-357) | 1025.54 (644.36-1533.14) | 82.53 |
| Somalia | 2254 (937-4294) | 1670.41 (672.26-3207.90) | 5968 (2489-11156) | 1430.42 (572.73-2734.63) | 164.80 |  | 5804 (3572-8947) | 4198.90 (2587.29-6475.50) | 15154 (9554-22296) | 3623.63 (2269.27-5346.96) | 161.08 |
| South Africa | 9716 (5338-16250) | 589.50 (321.52-987.29) | 13826 (7820-22042) | 367.88 (205.75-590.16) | 42.30 |  | 7994 (5136-12504) | 495.47 (318.32-774.23) | 18451 (14569-23086) | 498.80 (393.52-624.59) | 130.82 |
| South Sudan | 2525 (1021-4801) | 1140.11 (455.59-2185.82) | 2667 (1128-4882) | 1071.09 (445.90-1978.52) | 5.64 |  | 5620 (3331-8898) | 2506.92 (1484.21-3952.94) | 5896 (3618-8953) | 2342.56 (1436.34-3547.17) | 4.91 |
| Spain | 24098 (11427-43305) | 457.16 (216.43-821.04) | 37813 (16638-68336) | 426.72 (192.41-765.62) | 56.91 |  | 8970 (5279-13900) | 169.95 (99.94-263.60) | 7283 (4363-11136) | 82.42 (49.43-125.84) | -18.81 |
| Sri Lanka | 6709 (3870-11066) | 812.34 (464.73-1341.47) | 18811 (11774-29105) | 757.19 (471.65-1171.75) | 180.37 |  | 9966 (5742-15480) | 1225.44 (710.60-1889.64) | 10976 (5942-18159) | 447.88 (242.98-738.56) | 10.14 |
| Sudan | 9651 (3880-17614) | 1248.19 (486.69-2297.30) | 16082 (7949-27589) | 1136.88 (551.08-1961.67) | 66.64 |  | 18852 (9238-36457) | 2477.79 (1194.73-4834.29) | 16217 (8501-29528) | 1171.38 (610.96-2150.84) | -13.98 |
| Suriname | 38 (14-76) | 193.36 (68.83-383.92) | 85 (35-160) | 162.35 (65.57-306.58) | 121.76 |  | 76 (42-128) | 384.84 (211.18-644.41) | 118 (62-200) | 224.64 (118.24-382.25) | 54.02 |
| Sweden | 1235 (512-2246) | 81.32 (34.14-147.51) | 1915 (835-3406) | 91.84 (41.61-161.84) | 55.08 |  | 783 (575-1046) | 51.91 (38.14-69.44) | 1107 (800-1511) | 54.05 (39.14-73.81) | 41.37 |
| Switzerland | 1564 (709-2824) | 159.14 (73.16-286.03) | 2628 (1090-4842) | 158.50 (66.99-289.09) | 68.05 |  | 1058 (662-1601) | 109.09 (68.22-165.11) | 1598 (951-2454) | 97.59 (58.06-149.30) | 51.10 |
| Syrian Arab Republic | 4717 (2849-7389) | 1238.47 (744.69-1944.90) | 12134 (7644-18679) | 1180.83 (738.84-1832.83) | 157.24 |  | 4699 (2747-7643) | 1270.70 (740.03-2068.87) | 7966 (4811-12455) | 835.73 (500.43-1304.22) | 69.55 |
| Taiwan (Province of China) | 20662 (9641-36918) | 1561.47 (715.68-2812.08) | 55051 (26221-96570) | 1367.21 (647.32-2403.61) | 166.43 |  | 23475 (17876-28930) | 1841.96 (1394.18-2278.00) | 38824 (28325-50119) | 974.90 (712.59-1258.48) | 65.39 |
| Tajikistan | 1090 (533-1956) | 534.61 (260.47-958.54) | 1961 (1014-3261) | 521.04 (264.32-875.78) | 79.87 |  | 1124 (667-1796) | 550.63 (326.36-879.90) | 1441 (800-2396) | 378.93 (208.99-634.39) | 28.16 |
| Thailand | 20925 (10070-36337) | 810.77 (385.06-1421.03) | 74162 (35266-128131) | 766.17 (363.54-1326.68) | 254.43 |  | 34145 (22649-48916) | 1319.43 (872.01-1892.74) | 75997 (46946-113951) | 786.44 (486.38-1178.53) | 122.57 |
| Timor-Leste | 110 (43-209) | 664.50 (255.11-1283.67) | 453 (183-865) | 589.59 (233.98-1137.32) | 311.26 |  | 162 (80-326) | 979.23 (473.92-1969.91) | 600 (306-1107) | 788.47 (399.99-1452.78) | 270.21 |
| Togo | 1459 (565-2739) | 1633.74 (618.87-3093.53) | 3745 (1665-6776) | 1423.88 (614.20-2608.52) | 156.60 |  | 1801 (1136-2638) | 2007.11 (1266.80-2945.90) | 4020 (2384-6259) | 1529.20 (899.61-2383.48) | 123.23 |
| Tokelau | 2 (1-3) | 1310.35 (632.85-2338.04) | 2 (1-3) | 1129.48 (564.55-1912.41) | 1.90 |  | 1 (1-2) | 951.26 (556.87-1545.91) | 1 (1-1) | 668.25 (430.93-973.68) | -18.04 |
| Tonga | 88 (47-144) | 1997.32 (1055.74-3284.33) | 126 (73-200) | 1884.45 (1085.75-2991.88) | 43.66 |  | 113 (76-170) | 2496.39 (1682.99-3760.42) | 109 (69-162) | 1622.62 (1031.82-2419.03) | -3.21 |
| Trinidad and Tobago | 121 (44-232) | 158.51 (57.78-306.05) | 241 (90-456) | 132.92 (49.15-252.62) | 99.98 |  | 182 (109-289) | 241.73 (144.34-384.55) | 194 (114-315) | 106.54 (62.75-172.91) | 6.77 |
| Tunisia | 2922 (1580-4919) | 715.63 (380.20-1214.46) | 7416 (4143-11867) | 648.28 (359.87-1039.62) | 153.83 |  | 2479 (1392-4057) | 642.96 (358.36-1067.02) | 4140 (2304-6846) | 373.32 (206.80-618.89) | 67.00 |
| Turkey | 18127 (10167-29371) | 713.08 (395.40-1161.63) | 52377 (30762-81507) | 633.81 (369.72-990.08) | 188.94 |  | 30539 (20116-45062) | 1222.79 (803.77-1807.07) | 56274 (37255-78243) | 700.26 (462.82-974.74) | 84.27 |
| Turkmenistan | 700 (371-1157) | 501.14 (263.23-832.56) | 1389 (748-2334) | 476.98 (254.69-805.45) | 98.51 |  | 1021 (626-1575) | 724.44 (442.27-1119.83) | 1622 (938-2604) | 542.67 (313.16-874.73) | 58.78 |
| Tuvalu | 7 (3-13) | 1302.71 (549.95-2439.35) | 10 (5-20) | 1175.05 (515.71-2216.25) | 43.26 |  | 7 (4-10) | 1096.71 (659.29-1742.45) | 7 (4-10) | 726.51 (476.68-1073.47) | 1.53 |
| Uganda | 5730 (2461-10234) | 1140.75 (484.43-2055.07) | 10563 (4659-18751) | 1010.31 (437.72-1803.02) | 84.35 |  | 12022 (7773-17375) | 2393.14 (1535.28-3469.41) | 15321 (8821-24029) | 1480.94 (843.65-2327.29) | 27.45 |
| Ukraine | 8690 (3577-16919) | 138.00 (56.36-268.66) | 9240 (3714-17761) | 123.67 (49.72-238.46) | 6.33 |  | 6240 (4685-8136) | 97.42 (73.03-127.26) | 5974 (3987-8625) | 79.62 (53.16-114.91) | -4.27 |
| United Arab Emirates | 91 (58-143) | 422.68 (264.30-663.47) | 803 (510-1276) | 475.59 (294.93-768.78) | 777.87 |  | 317 (201-478) | 1451.07 (912.21-2201.20) | 1506 (940-2339) | 1069.71 (666.02-1674.19) | 375.23 |
| United Kingdom | 9645 (3960-18358) | 107.92 (44.70-205.26) | 17300 (7667-30687) | 140.96 (63.62-247.85) | 79.37 |  | 3993 (3053-5085) | 44.89 (34.30-57.15) | 12237 (8982-16043) | 99.63 (73.29-130.38) | 206.44 |
| United Republic of Tanzania | 7833 (3314-14458) | 907.91 (376.72-1693.99) | 15735 (7183-28026) | 834.17 (374.66-1498.32) | 100.88 |  | 18196 (11568-25717) | 2091.43 (1320.75-2974.38) | 26738 (15652-41933) | 1422.14 (830.19-2228.98) | 46.94 |
| United States of America | 25768 (11344-45967) | 82.41 (36.37-146.96) | 47853 (22851-83185) | 83.97 (40.07-145.75) | 85.71 |  | 21096 (16380-27141) | 67.51 (52.38-86.94) | 52989 (41470-66103) | 93.04 (72.80-116.01) | 151.18 |
| United States Virgin Islands | 12 (5-22) | 173.01 (67.03-324.97) | 29 (12-55) | 155.47 (65.86-291.50) | 150.02 |  | 19 (11-33) | 291.76 (158.54-498.32) | 24 (13-41) | 126.77 (69.53-215.53) | 22.97 |
| Uruguay | 484 (281-779) | 130.47 (75.67-210.20) | 679 (380-1118) | 130.08 (73.42-213.66) | 40.33 |  | 684 (382-1123) | 183.90 (102.55-302.21) | 695 (433-1065) | 136.51 (85.14-209.06) | 1.58 |
| Uzbekistan | 8024 (3334-16056) | 928.81 (388.28-1850.01) | 14883 (6477-27231) | 785.10 (334.89-1459.54) | 85.47 |  | 4725 (2797-7326) | 553.74 (327.47-859.95) | 7522 (4398-11773) | 386.82 (225.16-610.85) | 59.19 |
| Vanuatu | 63 (29-115) | 1385.84 (612.24-2554.70) | 158 (73-279) | 1240.83 (570.98-2226.39) | 150.16 |  | 59 (28-127) | 1263.35 (599.57-2716.53) | 119 (66-219) | 910.03 (501.74-1681.04) | 100.80 |
| Venezuela (Bolivarian Republic of) | 876 (311-1761) | 116.63 (41.11-235.56) | 2546 (940-5293) | 96.82 (35.37-201.93) | 190.60 |  | 1418 (875-2144) | 187.64 (115.69-283.97) | 2544 (1447-4288) | 96.75 (55.17-162.96) | 79.36 |
| Viet Nam | 51311 (24143-90305) | 1507.58 (699.14-2660.82) | 115993 (55041-194974) | 1478.82 (692.84-2500.01) | 126.06 |  | 69448 (42648-110927) | 2070.10 (1264.20-3332.65) | 92517 (55792-137394) | 1202.97 (724.72-1792.97) | 33.22 |
| Yemen | 4867 (2315-8520) | 1326.74 (611.72-2364.91) | 12694 (6414-21313) | 1213.78 (597.80-2060.10) | 160.80 |  | 10466 (5073-20834) | 2924.77 (1389.13-5907.63) | 12459 (7044-20631) | 1239.92 (689.52-2065.72) | 19.04 |
| Zambia | 2433 (1163-4319) | 1205.86 (568.81-2154.43) | 5013 (2781-8157) | 1075.25 (590.20-1760.28) | 106.09 |  | 6374 (4212-9316) | 3116.45 (2053.24-4547.69) | 8538 (4797-13344) | 1821.55 (1014.04-2857.84) | 33.95 |
| Zimbabwe | 7886 (3237-14614) | 2421.11 (948.30-4559.94) | 11470 (5309-20903) | 2211.02 (1006.91-4072.88) | 45.46 |  | 5376 (3503-7767) | 1664.79 (1082.60-2409.52) | 7237 (3924-11591) | 1409.68 (760.39-2264.50) | 34.63 |

DALYs disability-adjusted life-years

Table S6 Age-standardized prevalence and mortality of total burden related to hepatitis B in adults aged ≥65 years between 1990 and 2021 in 204 countries and territories

| location | Prevalence | | | | |  | Mortality | | | | |
| --- | --- | --- | --- | --- | --- | --- | --- | --- | --- | --- | --- |
|  | Cases in 1990 | Age standardized rate in 1990 (per 100 000) | Cases in 2021 | Age standardized rate in 2021 (per 100 000) | Cases change (%) |  | Cases in 1990 | Age standardized rate in 1990 (per 100 000) | Cases in 2021 | Age standardized rate in 2021 (per 100 000) | Cases change (%) |
| Afghanistan | 23847 (20482-27382) | 4289.88 (3677.94-4945.71) | 22754 (20044-25707) | 3600.53 (3164.36-4078.53) | -4.58 |  | 1211 (665-2049) | 239.05 (128.00-414.57) | 772 (459-1238) | 129.23 (76.13-209.41) | -36.25 |
| Albania | 2532 (2216-2917) | 1564.64 (1366.22-1807.23) | 5759 (5061-6590) | 1369.69 (1202.08-1570.16) | 127.45 |  | 69 (45-99) | 44.12 (28.53-63.64) | 79 (46-128) | 19.37 (11.28-31.16) | 15.40 |
| Algeria | 34281 (31263-37774) | 3348.43 (3049.10-3695.72) | 88490 (80819-96253) | 3075.90 (2807.39-3348.97) | 158.13 |  | 449 (244-770) | 60.59 (33.49-101.92) | 867 (491-1414) | 37.32 (20.66-61.41) | 93.03 |
| American Samoa | 81 (68-95) | 5069.26 (4215.86-5956.27) | 170 (147-199) | 4567.23 (3933.44-5354.82) | 110.02 |  | 1 (0-1) | 33.69 (22.40-48.40) | 1 (1-2) | 33.36 (22.57-47.26) | 131.69 |
| Andorra | 67 (57-79) | 1284.57 (1090.25-1510.43) | 156 (133-184) | 1149.19 (981.25-1354.42) | 131.46 |  | 1 (0-1) | 14.19 (7.91-23.77) | 1 (1-2) | 10.15 (5.53-17.34) | 98.29 |
| Angola | 36136 (31290-41278) | 14441.77 (12424.92-16647.44) | 88665 (76428-102261) | 11606.50 (9943.64-13479.63) | 145.37 |  | 384 (227-597) | 160.62 (94.28-253.67) | 581 (323-928) | 80.64 (44.84-129.37) | 51.57 |
| Antigua and Barbuda | 53 (46-63) | 1040.14 (886.37-1220.28) | 83 (71-95) | 917.63 (777.96-1053.95) | 55.77 |  | 1 (1-1) | 17.21 (10.34-26.96) | 1 (1-2) | 11.01 (6.64-17.26) | 7.28 |
| Argentina | 15003 (13231-16922) | 510.45 (449.31-577.19) | 27784 (23945-31799) | 530.34 (457.05-606.94) | 85.19 |  | 399 (210-668) | 13.90 (7.31-23.31) | 451 (247-736) | 8.59 (4.71-14.02) | 13.11 |
| Armenia | 4077 (3467-4700) | 2198.36 (1867.59-2535.80) | 7909 (6971-8963) | 1972.34 (1738.35-2236.63) | 94.01 |  | 42 (27-60) | 22.41 (14.49-32.22) | 74 (46-114) | 18.89 (11.64-29.09) | 76.76 |
| Australia | 44402 (37826-51744) | 2377.95 (2023.47-2774.34) | 94956 (81285-110309) | 2176.45 (1866.25-2524.21) | 113.86 |  | 140 (85-203) | 7.52 (4.57-10.87) | 334 (212-482) | 7.54 (4.79-10.87) | 137.46 |
| Austria | 9969 (8342-11839) | 864.61 (723.30-1025.93) | 13637 (11448-16237) | 811.01 (682.40-962.88) | 36.80 |  | 90 (49-153) | 7.72 (4.19-13.15) | 86 (51-136) | 4.98 (2.94-7.79) | -4.07 |
| Azerbaijan | 10856 (9571-12062) | 3134.90 (2763.52-3484.55) | 21799 (19439-24276) | 3049.21 (2721.37-3395.90) | 100.80 |  | 125 (73-198) | 36.94 (21.38-58.90) | 219 (121-370) | 32.77 (17.83-55.74) | 75.83 |
| Bahamas | 125 (105-148) | 1005.65 (846.54-1191.57) | 288 (245-340) | 889.73 (753.24-1049.35) | 131.39 |  | 2 (1-3) | 16.32 (9.73-25.74) | 3 (2-5) | 10.33 (6.16-16.46) | 61.35 |
| Bahrain | 399 (365-438) | 3650.20 (3331.33-4014.80) | 2019 (1823-2203) | 3542.77 (3200.84-3866.38) | 405.84 |  | 13 (9-19) | 151.96 (96.00-222.16) | 25 (15-39) | 66.55 (40.30-103.09) | 90.52 |
| Bangladesh | 161034 (143150-182264) | 4574.71 (4059.22-5187.42) | 431968 (393187-475711) | 3694.37 (3357.40-4075.13) | 168.25 |  | 3494 (2110-5201) | 104.47 (62.87-155.70) | 5793 (3324-9148) | 57.32 (32.61-90.46) | 65.77 |
| Barbados | 242 (203-285) | 826.57 (693.33-970.20) | 372 (319-438) | 753.37 (645.65-886.15) | 53.79 |  | 3 (2-5) | 10.74 (6.57-16.62) | 3 (2-5) | 6.54 (3.93-10.31) | -0.09 |
| Belarus | 14186 (11729-17057) | 1288.32 (1064.97-1549.59) | 14535 (12298-17156) | 972.31 (822.24-1148.23) | 2.46 |  | 52 (33-76) | 4.68 (2.96-6.89) | 72 (44-112) | 4.81 (2.93-7.48) | 39.27 |
| Belgium | 9975 (8544-11544) | 675.22 (578.23-781.39) | 13275 (11438-15431) | 602.62 (521.50-698.17) | 33.09 |  | 96 (58-148) | 6.45 (3.90-9.97) | 107 (65-164) | 4.72 (2.89-7.23) | 11.33 |
| Belize | 113 (96-133) | 1419.75 (1198.70-1674.55) | 270 (231-319) | 1186.55 (1017.47-1406.65) | 138.22 |  | 1 (1-2) | 16.28 (9.17-26.25) | 3 (2-4) | 12.48 (7.12-20.05) | 112.47 |
| Benin | 14984 (12427-17681) | 9111.29 (7539.39-10774.92) | 28254 (24168-32686) | 7432.56 (6351.96-8615.63) | 88.56 |  | 279 (179-405) | 176.91 (112.71-257.81) | 362 (237-518) | 102.06 (66.81-146.04) | 29.61 |
| Bermuda | 45 (37-53) | 821.80 (669.80-974.57) | 95 (81-114) | 731.01 (623.70-875.01) | 112.34 |  | 1 (0-1) | 13.42 (8.01-20.55) | 1 (0-1) | 4.64 (2.80-7.38) | -13.27 |
| Bhutan | 797 (685-931) | 4804.76 (4112.27-5663.55) | 1779 (1553-2050) | 3524.43 (3069.87-4069.83) | 123.20 |  | 11 (6-19) | 68.86 (37.11-122.80) | 29 (16-49) | 59.42 (33.17-101.25) | 172.97 |
| Bolivia (Plurinational State of) | 1700 (1540-1875) | 673.63 (609.07-744.42) | 5166 (4648-5722) | 670.37 (601.77-743.83) | 203.88 |  | 91 (41-169) | 38.11 (17.03-70.07) | 205 (101-369) | 28.30 (14.02-50.87) | 123.73 |
| Bosnia and Herzegovina | 3870 (3348-4518) | 1303.34 (1123.47-1528.72) | 6335 (5490-7351) | 1037.87 (897.98-1206.04) | 63.71 |  | 88 (56-128) | 29.76 (18.91-43.04) | 115 (68-174) | 19.03 (11.32-28.73) | 30.12 |
| Botswana | 2663 (2240-3127) | 6140.08 (5118.35-7251.82) | 4758 (4034-5563) | 4436.08 (3747.87-5217.67) | 78.68 |  | 29 (16-51) | 76.25 (41.29-134.69) | 38 (22-59) | 38.29 (22.31-60.43) | 30.13 |
| Brazil | 167262 (146721-191047) | 2371.90 (2073.64-2719.33) | 327005 (286188-373425) | 1495.45 (1307.84-1709.39) | 95.50 |  | 1196 (924-1498) | 17.61 (13.56-22.09) | 2334 (1807-2968) | 10.73 (8.30-13.64) | 95.07 |
| Brunei Darussalam | 308 (257-368) | 4213.26 (3506.30-5038.88) | 970 (832-1133) | 3825.03 (3262.46-4496.93) | 214.72 |  | 5 (3-7) | 66.15 (42.45-95.32) | 8 (5-12) | 35.17 (22.66-51.89) | 72.51 |
| Bulgaria | 35006 (29450-41380) | 2997.03 (2509.86-3561.55) | 34637 (29412-40916) | 2351.40 (1996.03-2778.12) | -1.05 |  | 309 (197-437) | 27.26 (17.45-38.76) | 281 (165-439) | 19.27 (11.29-30.06) | -8.80 |
| Burkina Faso | 45023 (39292-52050) | 13064.29 (11299.50-15207.87) | 78443 (68592-89740) | 11121.69 (9668.62-12774.72) | 74.23 |  | 599 (381-925) | 188.29 (119.74-288.54) | 804 (507-1197) | 121.94 (77.57-179.94) | 34.04 |
| Burundi | 6430 (5444-7437) | 3464.44 (2922.42-4029.14) | 11159 (9453-13184) | 3299.31 (2777.22-3924.99) | 73.54 |  | 253 (147-399) | 143.48 (82.83-226.42) | 291 (167-451) | 95.19 (53.96-147.99) | 14.94 |
| Cabo Verde | 1860 (1563-2185) | 8897.05 (7483.57-10455.49) | 2361 (2003-2739) | 7001.14 (5935.25-8120.39) | 26.96 |  | 19 (12-32) | 87.62 (54.30-146.19) | 28 (15-42) | 82.00 (45.52-122.75) | 46.82 |
| Cambodia | 26209 (22088-30303) | 7913.91 (6637.67-9219.16) | 61015 (52570-70146) | 6364.44 (5458.60-7349.62) | 132.80 |  | 609 (334-1093) | 197.49 (106.44-366.05) | 951 (537-1573) | 109.80 (61.27-183.00) | 56.13 |
| Cameroon | 29801 (25978-34075) | 9359.47 (8108.11-10761.93) | 67354 (59334-76095) | 7595.53 (6652.43-8631.33) | 126.01 |  | 414 (257-595) | 139.45 (85.88-201.08) | 724 (398-1121) | 88.73 (48.58-137.80) | 74.98 |
| Canada | 67651 (60973-75284) | 2196.70 (1978.36-2447.17) | 137474 (124093-153403) | 1960.46 (1771.41-2185.13) | 103.21 |  | 107 (63-175) | 3.48 (2.03-5.68) | 225 (135-350) | 3.18 (1.91-4.94) | 109.87 |
| Central African Republic | 8290 (7157-9569) | 10913.04 (9361.01-12715.17) | 12575 (10921-14656) | 9633.65 (8293.54-11317.83) | 51.69 |  | 95 (57-148) | 129.53 (76.92-205.17) | 124 (73-192) | 99.11 (56.76-156.15) | 30.81 |
| Chad | 32338 (27643-37326) | 13233.75 (11279.74-15348.58) | 49231 (41183-57148) | 11715.05 (9756.11-13651.05) | 52.24 |  | 426 (262-686) | 183.77 (111.99-297.09) | 631 (391-984) | 161.22 (99.67-252.12) | 48.06 |
| Chile | 5261 (4558-6076) | 616.36 (533.15-713.72) | 14643 (12609-16904) | 631.19 (543.57-728.43) | 178.36 |  | 202 (109-332) | 23.82 (12.90-39.24) | 327 (193-512) | 14.05 (8.30-22.00) | 61.86 |
| China | 5920751 (5240007-6684683) | 8778.08 (7737.24-9961.27) | 12873285 (11455477-14367767) | 6453.00 (5730.76-7220.85) | 117.43 |  | 65658 (56248-76287) | 108.53 (92.71-126.45) | 98822 (79228-121735) | 53.37 (42.77-65.77) | 50.51 |
| Colombia | 60686 (51814-70088) | 4502.95 (3840.93-5205.85) | 176731 (150897-207259) | 3657.79 (3124.87-4288.82) | 191.22 |  | 85 (54-127) | 6.41 (4.07-9.57) | 222 (136-343) | 4.64 (2.83-7.15) | 161.94 |
| Comoros | 831 (680-984) | 5594.28 (4554.48-6675.87) | 1722 (1439-2004) | 4567.94 (3809.29-5342.39) | 107.17 |  | 16 (9-23) | 113.40 (68.25-169.96) | 30 (17-46) | 83.75 (46.62-129.29) | 90.90 |
| Congo | 7454 (6354-8777) | 9291.20 (7863.61-11055.08) | 12856 (11112-15085) | 7244.50 (6211.32-8573.85) | 72.47 |  | 88 (53-131) | 116.79 (69.92-176.90) | 114 (64-187) | 68.34 (38.54-111.54) | 29.57 |
| Cook Islands | 41 (34-48) | 4146.55 (3389.62-4911.25) | 79 (67-92) | 3365.75 (2849.65-3928.41) | 92.27 |  | 0 (0-0) | 26.75 (15.24-42.76) | 0 (0-1) | 21.09 (11.26-35.58) | 88.85 |
| Costa Rica | 1704 (1431-2019) | 1181.98 (991.88-1400.84) | 4637 (3900-5523) | 965.02 (812.06-1149.24) | 172.05 |  | 12 (8-19) | 8.66 (5.34-13.06) | 38 (23-59) | 7.93 (4.78-12.28) | 204.37 |
| Coted'Ivoire | 26707 (23218-30349) | 10505.80 (9071.68-12021.44) | 63834 (55108-75227) | 8533.76 (7313.21-10133.90) | 139.02 |  | 319 (213-461) | 138.04 (91.45-198.54) | 534 (321-814) | 78.04 (46.60-118.39) | 67.15 |
| Croatia | 7813 (6673-9339) | 1505.21 (1280.27-1804.90) | 11826 (10118-13815) | 1330.88 (1139.84-1553.01) | 51.36 |  | 154 (91-233) | 30.28 (17.89-45.88) | 161 (99-242) | 17.94 (11.05-27.04) | 4.57 |
| Cuba | 8328 (7021-10044) | 893.77 (752.55-1079.10) | 13142 (10666-15510) | 750.24 (609.72-884.62) | 57.81 |  | 99 (61-153) | 10.77 (6.57-16.66) | 107 (63-170) | 6.03 (3.54-9.53) | 7.98 |
| Cyprus | 1311 (1121-1571) | 1648.56 (1400.73-1988.13) | 2670 (2311-3135) | 1341.28 (1158.86-1576.61) | 103.62 |  | 7 (4-11) | 10.33 (5.70-17.21) | 9 (5-15) | 4.71 (2.70-7.79) | 33.63 |
| Czechia | 12729 (10721-15281) | 970.25 (815.74-1166.93) | 17332 (14849-19961) | 787.40 (674.53-906.69) | 36.17 |  | 309 (202-436) | 23.37 (15.25-33.05) | 342 (204-522) | 15.56 (9.24-23.69) | 10.89 |
| Democratic People's Republic of Korea | 131141 (109342-156014) | 11330.89 (9408.05-13567.07) | 262099 (221780-314750) | 9752.08 (8239.58-11724.55) | 99.86 |  | 1004 (657-1479) | 92.88 (59.94-140.34) | 1529 (996-2253) | 57.54 (37.28-85.51) | 52.36 |
| Democratic Republic of the Congo | 175406 (152433-200987) | 16060.32 (13884.30-18549.45) | 338607 (296450-384245) | 14098.43 (12303.32-16062.35) | 93.04 |  | 801 (435-1342) | 77.14 (41.40-129.72) | 1353 (704-2261) | 58.19 (30.32-97.75) | 68.88 |
| Denmark | 6245 (5295-7496) | 781.18 (662.77-937.35) | 9024 (7589-10667) | 773.28 (651.39-912.73) | 44.49 |  | 30 (18-48) | 3.81 (2.28-6.02) | 62 (37-94) | 5.23 (3.14-7.97) | 102.10 |
| Djibouti | 441 (374-514) | 5295.96 (4465.27-6218.37) | 1971 (1639-2317) | 4751.22 (3931.92-5627.31) | 347.41 |  | 8 (5-13) | 108.21 (62.99-174.19) | 33 (18-52) | 89.50 (48.45-141.64) | 305.23 |
| Dominica | 63 (53-75) | 1103.59 (926.71-1304.32) | 70 (59-81) | 940.57 (799.71-1095.38) | 9.88 |  | 1 (1-2) | 16.87 (9.92-27.25) | 1 (0-1) | 10.98 (6.50-17.57) | -17.92 |
| Dominican Republic | 5457 (4640-6338) | 1857.94 (1574.64-2163.22) | 13057 (11114-15326) | 1546.22 (1316.01-1815.65) | 139.25 |  | 74 (38-127) | 27.79 (14.44-47.96) | 144 (67-265) | 17.22 (8.00-31.58) | 95.59 |
| Ecuador | 3900 (3530-4296) | 924.64 (836.52-1019.05) | 12935 (11741-14430) | 905.78 (821.88-1010.79) | 231.67 |  | 121 (77-181) | 29.32 (18.62-44.14) | 269 (162-421) | 19.29 (11.57-30.20) | 123.47 |
| Egypt | 82772 (66724-101212) | 4270.35 (3414.68-5261.64) | 145809 (116856-176914) | 3107.52 (2473.61-3809.73) | 76.16 |  | 10579 (6639-14836) | 793.46 (488.40-1124.03) | 10236 (6241-15258) | 282.01 (171.98-418.13) | -3.24 |
| El Salvador | 3325 (2776-3959) | 1349.83 (1126.42-1607.46) | 5847 (4929-6861) | 1059.79 (894.16-1241.96) | 75.88 |  | 21 (12-36) | 8.64 (4.84-14.85) | 33 (17-57) | 5.80 (3.07-10.15) | 53.98 |
| Equatorial Guinea | 578 (493-673) | 4173.03 (3545.97-4898.78) | 986 (872-1126) | 2839.02 (2499.28-3256.88) | 70.62 |  | 11 (5-18) | 78.06 (36.69-135.93) | 13 (6-24) | 40.66 (18.25-71.75) | 25.75 |
| Eritrea | 3934 (3273-4634) | 6455.97 (5334.94-7686.38) | 9766 (8138-11574) | 5554.66 (4608.92-6636.25) | 148.26 |  | 86 (53-129) | 155.06 (93.08-238.14) | 174 (102-260) | 110.46 (63.76-167.43) | 102.81 |
| Estonia | 2313 (1946-2831) | 1276.59 (1073.20-1562.80) | 2573 (2118-3102) | 960.91 (792.66-1156.72) | 11.21 |  | 9 (6-14) | 4.99 (3.13-7.50) | 15 (9-23) | 5.69 (3.53-8.72) | 66.94 |
| Eswatini | 1440 (1200-1717) | 6949.55 (5771.82-8331.89) | 2141 (1780-2517) | 5171.27 (4278.85-6123.21) | 48.70 |  | 18 (10-29) | 94.46 (53.01-158.03) | 28 (14-48) | 72.61 (37.73-123.15) | 55.63 |
| Ethiopia | 90533 (78608-104386) | 6141.78 (5291.62-7134.03) | 170633 (149552-193433) | 5176.68 (4525.72-5889.41) | 88.48 |  | 2228 (1413-3090) | 166.14 (104.56-231.43) | 2970 (2140-3841) | 96.14 (67.96-125.21) | 33.30 |
| Fiji | 791 (669-901) | 3338.52 (2805.58-3826.81) | 1753 (1508-2087) | 3064.79 (2620.51-3665.29) | 121.68 |  | 7 (4-11) | 29.01 (17.60-46.42) | 13 (8-19) | 23.89 (15.12-35.20) | 94.60 |
| Finland | 6213 (5147-7327) | 912.86 (755.96-1076.83) | 10586 (8806-12573) | 831.66 (692.19-986.66) | 70.39 |  | 37 (23-56) | 5.33 (3.33-8.16) | 87 (53-136) | 6.78 (4.10-10.57) | 138.43 |
| France | 56543 (49253-64859) | 718.12 (626.40-822.39) | 87848 (77221-99580) | 652.99 (576.52-737.38) | 55.36 |  | 708 (433-1075) | 9.06 (5.52-13.77) | 920 (548-1427) | 6.59 (3.93-10.16) | 30.00 |
| Gabon | 3696 (3218-4290) | 7810.34 (6773.56-9111.31) | 4857 (4222-5577) | 6311.39 (5452.13-7290.77) | 31.42 |  | 49 (28-75) | 107.26 (61.87-166.28) | 46 (25-75) | 63.40 (33.24-102.85) | -5.03 |
| Gambia | 2263 (2013-2539) | 8528.85 (7537.13-9611.93) | 5014 (4538-5540) | 6807.45 (6142.66-7554.06) | 121.55 |  | 38 (25-54) | 151.13 (99.89-216.76) | 90 (49-141) | 127.59 (68.47-200.99) | 137.32 |
| Georgia | 7824 (6708-9165) | 1536.41 (1315.61-1801.92) | 7907 (6864-9175) | 1389.66 (1207.24-1610.50) | 1.06 |  | 113 (67-176) | 22.31 (13.18-34.64) | 80 (47-130) | 14.03 (8.31-22.83) | -29.78 |
| Germany | 49361 (43314-57280) | 416.35 (365.60-482.76) | 72183 (63193-82618) | 400.16 (352.05-456.39) | 46.23 |  | 956 (564-1512) | 7.87 (4.63-12.45) | 1075 (651-1666) | 5.75 (3.49-8.89) | 12.44 |
| Ghana | 57727 (50391-65937) | 12803.58 (11101.71-14723.65) | 128052 (111129-146368) | 10390.60 (8946.73-11956.60) | 121.82 |  | 573 (364-854) | 139.21 (87.24-209.82) | 1119 (682-1649) | 102.86 (62.22-152.46) | 95.28 |
| Greece | 31122 (26827-35401) | 2206.71 (1900.09-2511.75) | 43518 (36858-50578) | 1902.08 (1621.97-2195.77) | 39.83 |  | 141 (95-202) | 10.14 (6.78-14.52) | 273 (172-394) | 11.11 (6.99-15.87) | 93.65 |
| Greenland | 34 (29-40) | 1603.98 (1352.28-1903.43) | 80 (66-96) | 1480.57 (1225.48-1795.43) | 135.51 |  | 0 (0-0) | 4.97 (2.91-8.02) | 0 (0-0) | 4.89 (2.58-8.50) | 145.07 |
| Grenada | 85 (70-103) | 1256.16 (1038.50-1513.23) | 97 (82-116) | 998.18 (839.61-1194.29) | 14.27 |  | 1 (1-2) | 14.60 (8.62-23.28) | 1 (1-2) | 11.78 (7.24-18.09) | 8.31 |
| Guam | 292 (255-329) | 5446.40 (4753.17-6148.38) | 928 (817-1059) | 4865.23 (4286.97-5547.74) | 218.13 |  | 3 (2-4) | 65.47 (44.07-88.72) | 6 (4-8) | 32.46 (22.21-43.96) | 97.31 |
| Guatemala | 5599 (4647-6671) | 2174.59 (1796.90-2608.66) | 15533 (13198-18795) | 1673.68 (1419.97-2028.00) | 177.40 |  | 44 (27-68) | 18.23 (11.12-28.13) | 116 (68-190) | 12.79 (7.51-20.93) | 163.10 |
| Guinea | 34329 (29904-40271) | 12071.40 (10453.07-14200.61) | 46473 (40335-53088) | 10701.64 (9253.18-12268.20) | 35.38 |  | 508 (349-720) | 189.41 (129.12-269.75) | 573 (380-830) | 139.09 (92.11-201.57) | 12.88 |
| Guinea-Bissau | 4002 (3303-4720) | 13340.29 (10956.23-15787.94) | 5335 (4566-6203) | 11018.08 (9340.86-12872.65) | 33.30 |  | 72 (46-102) | 252.19 (160.70-360.31) | 66 (43-95) | 150.91 (97.20-218.19) | -8.08 |
| Guyana | 522 (429-619) | 1748.92 (1434.36-2083.31) | 703 (602-825) | 1367.16 (1164.75-1612.98) | 34.81 |  | 9 (5-15) | 31.69 (17.23-53.46) | 8 (4-14) | 16.11 (8.67-28.05) | -11.55 |
| Haiti | 4537 (3843-5470) | 1820.72 (1531.57-2208.56) | 8080 (6888-9474) | 1540.86 (1305.86-1818.85) | 78.11 |  | 80 (39-148) | 34.26 (16.25-64.71) | 118 (55-226) | 23.85 (11.12-45.54) | 48.09 |
| Honduras | 2979 (2519-3561) | 1865.55 (1573.26-2237.49) | 7814 (6504-9271) | 1513.00 (1254.31-1801.05) | 162.33 |  | 17 (9-29) | 10.88 (5.76-18.79) | 57 (29-104) | 11.54 (5.77-20.99) | 240.86 |
| Hungary | 7987 (6982-9181) | 566.19 (494.03-651.83) | 10647 (9390-12079) | 531.64 (468.88-603.14) | 33.31 |  | 399 (238-606) | 28.38 (16.94-43.15) | 369 (214-575) | 18.43 (10.67-28.74) | -7.47 |
| Iceland | 194 (166-230) | 720.14 (618.28-855.77) | 358 (302-426) | 652.93 (551.58-776.86) | 84.84 |  | 1 (1-1) | 3.32 (2.02-5.06) | 2 (1-4) | 4.46 (2.48-7.43) | 176.34 |
| India | 1008462 (891429-1148379) | 3094.78 (2724.63-3539.91) | 2616699 (2332736-2920279) | 2634.51 (2344.05-2949.52) | 159.47 |  | 10789 (7748-14982) | 35.22 (25.11-49.07) | 29965 (19288-41416) | 32.27 (20.61-44.73) | 177.73 |
| Indonesia | 349694 (311863-395618) | 5029.88 (4470.48-5712.09) | 804333 (721180-893703) | 4466.26 (3992.04-4982.79) | 130.01 |  | 6490 (4119-10168) | 105.71 (65.86-166.66) | 12970 (9244-17330) | 87.92 (62.44-117.83) | 99.85 |
| Iran (Islamic Republic of) | 69104 (62282-76604) | 3531.42 (3171.27-3937.90) | 153646 (139517-168546) | 2540.32 (2304.39-2789.82) | 122.34 |  | 691 (489-957) | 42.60 (29.62-59.64) | 1385 (1104-1788) | 24.20 (19.19-31.40) | 100.34 |
| Iraq | 7410 (6793-8119) | 1192.74 (1093.06-1307.00) | 19621 (18165-21321) | 1141.80 (1055.28-1242.16) | 164.79 |  | 323 (187-513) | 52.57 (30.41-83.60) | 624 (367-954) | 40.10 (23.47-61.52) | 93.20 |
| Ireland | 3151 (2667-3751) | 778.73 (658.33-928.45) | 5154 (4392-6059) | 688.24 (586.91-808.62) | 63.56 |  | 14 (9-21) | 3.41 (2.13-5.17) | 26 (15-42) | 3.49 (1.99-5.55) | 93.09 |
| Israel | 2884 (2540-3348) | 614.86 (539.84-715.30) | 6500 (5721-7426) | 545.55 (480.74-622.45) | 125.33 |  | 33 (20-50) | 7.13 (4.42-10.75) | 46 (29-71) | 3.84 (2.37-5.84) | 40.38 |
| Italy | 235455 (207782-267400) | 2748.52 (2425.24-3122.12) | 306771 (267097-351755) | 2237.65 (1956.31-2557.47) | 30.29 |  | 1375 (1098-1709) | 15.99 (12.74-19.91) | 1003 (762-1271) | 6.65 (5.10-8.40) | -27.06 |
| Jamaica | 1961 (1651-2280) | 1161.31 (978.68-1349.77) | 2615 (2219-3072) | 980.88 (834.44-1150.34) | 33.36 |  | 11 (7-17) | 6.61 (3.93-10.21) | 12 (7-19) | 4.54 (2.71-7.18) | 8.63 |
| Japan | 433281 (390117-481632) | 2868.82 (2578.95-3192.72) | 845837 (749416-956487) | 2344.95 (2090.30-2637.40) | 95.22 |  | 2836 (2325-3416) | 19.09 (15.57-23.06) | 4687 (3516-5881) | 11.41 (8.75-14.22) | 65.26 |
| Jordan | 4010 (3451-4674) | 4508.75 (3866.12-5289.56) | 18853 (16399-21743) | 3627.94 (3141.06-4207.82) | 370.18 |  | 48 (29-76) | 61.10 (36.24-97.18) | 93 (55-146) | 20.57 (12.06-32.25) | 91.74 |
| Kazakhstan | 10950 (9550-12490) | 1154.48 (1005.87-1317.91) | 15399 (13589-17556) | 1051.82 (926.26-1205.02) | 40.63 |  | 234 (154-337) | 24.71 (16.22-35.73) | 415 (235-678) | 30.71 (17.40-50.28) | 77.91 |
| Kenya | 39294 (34618-44495) | 6210.90 (5451.74-7052.66) | 86437 (76380-97977) | 5027.42 (4425.42-5727.31) | 119.97 |  | 799 (497-1375) | 137.02 (85.10-234.37) | 1896 (1318-2779) | 125.69 (86.81-184.55) | 137.21 |
| Kiribati | 225 (191-264) | 8268.60 (6940.46-9770.57) | 352 (300-405) | 7337.39 (6226.69-8520.22) | 56.47 |  | 2 (1-3) | 67.43 (42.24-107.19) | 2 (2-4) | 53.06 (33.57-80.67) | 34.01 |
| Kuwait | 477 (427-534) | 1368.29 (1221.62-1537.48) | 2141 (1934-2416) | 1247.77 (1127.10-1409.56) | 349.19 |  | 9 (6-13) | 28.87 (18.82-40.83) | 28 (16-44) | 17.18 (10.04-27.23) | 196.31 |
| Kyrgyzstan | 5539 (4629-6491) | 2408.04 (2010.33-2824.88) | 7745 (6520-9270) | 2216.20 (1859.57-2660.92) | 39.82 |  | 73 (45-113) | 32.18 (19.62-50.10) | 69 (40-111) | 20.44 (11.90-32.98) | -5.30 |
| Lao People's Democratic Republic | 10037 (8495-11906) | 6197.01 (5212.42-7400.76) | 16975 (14192-20287) | 5050.40 (4204.47-6074.18) | 69.12 |  | 154 (86-264) | 102.37 (55.05-181.12) | 184 (106-295) | 59.11 (33.92-94.79) | 19.72 |
| Latvia | 4027 (3386-4769) | 1268.71 (1066.88-1502.49) | 3712 (3202-4314) | 957.33 (826.81-1109.88) | -7.81 |  | 16 (10-23) | 5.02 (3.27-7.22) | 18 (11-27) | 4.54 (2.76-6.97) | 10.78 |
| Lebanon | 5083 (4621-5528) | 3009.90 (2732.23-3276.06) | 15160 (13765-16546) | 2795.44 (2539.79-3049.44) | 198.26 |  | 104 (60-164) | 69.16 (39.74-109.61) | 197 (118-302) | 33.86 (20.34-51.89) | 88.31 |
| Lesotho | 5004 (4182-5860) | 7124.76 (5937.20-8375.62) | 4695 (3935-5477) | 5406.45 (4506.87-6339.42) | -6.17 |  | 57 (30-111) | 86.29 (44.45-169.72) | 66 (37-107) | 80.39 (45.08-131.01) | 14.71 |
| Liberia | 13901 (11552-16282) | 13925.14 (11541.64-16394.11) | 17458 (15136-20071) | 12355.55 (10671.31-14264.37) | 25.58 |  | 169 (110-247) | 184.51 (118.65-267.23) | 167 (106-242) | 127.53 (81.26-184.52) | -1.42 |
| Libya | 4074 (3540-4700) | 2900.56 (2516.44-3351.19) | 8624 (7656-9765) | 2449.32 (2171.73-2777.30) | 111.69 |  | 104 (48-218) | 76.68 (35.37-161.89) | 191 (103-324) | 56.48 (30.23-96.76) | 83.65 |
| Lithuania | 4280 (3529-5114) | 1084.69 (893.91-1295.83) | 4463 (3750-5415) | 809.73 (682.51-980.18) | 4.27 |  | 15 (10-23) | 3.88 (2.46-5.81) | 27 (17-40) | 4.90 (3.04-7.38) | 75.19 |
| Luxembourg | 461 (387-545) | 916.40 (770.07-1084.52) | 813 (699-967) | 855.89 (737.65-1015.53) | 76.45 |  | 4 (3-7) | 8.61 (5.05-13.65) | 6 (3-9) | 5.68 (3.45-8.78) | 28.25 |
| Madagascar | 32262 (28059-37650) | 8430.95 (7302.52-9916.40) | 52981 (45930-60986) | 7458.61 (6426.69-8674.09) | 64.22 |  | 387 (227-641) | 107.27 (62.65-177.44) | 496 (283-813) | 77.34 (43.99-126.21) | 28.31 |
| Malawi | 17680 (15182-20627) | 5990.06 (5115.35-7037.49) | 28669 (24572-32955) | 5127.11 (4372.61-5935.38) | 62.16 |  | 401 (249-576) | 148.63 (91.88-214.36) | 606 (375-900) | 118.05 (71.74-177.24) | 50.91 |
| Malaysia | 21913 (19504-24516) | 3226.17 (2870.28-3610.90) | 70163 (63825-77195) | 2938.03 (2669.19-3237.52) | 220.19 |  | 392 (274-530) | 58.12 (40.59-78.70) | 1343 (898-1860) | 60.40 (40.11-84.07) | 242.42 |
| Maldives | 207 (177-242) | 3563.86 (3027.05-4178.99) | 595 (496-700) | 2681.55 (2232.78-3157.76) | 187.21 |  | 4 (2-6) | 76.11 (45.89-120.70) | 6 (4-9) | 28.51 (17.67-42.15) | 54.51 |
| Mali | 25193 (21615-29612) | 8400.15 (7134.59-9937.40) | 49273 (41926-56769) | 7539.77 (6373.69-8736.62) | 95.58 |  | 463 (305-654) | 167.11 (108.32-237.81) | 847 (543-1220) | 141.82 (90.91-204.75) | 82.74 |
| Malta | 292 (240-349) | 739.91 (607.34-887.08) | 654 (554-766) | 657.66 (558.51-770.12) | 124.17 |  | 2 (1-3) | 5.22 (3.17-8.03) | 4 (2-6) | 3.56 (2.12-5.55) | 74.27 |
| Marshall Islands | 77 (66-90) | 6032.23 (5119.15-7092.88) | 128 (108-151) | 5335.43 (4484.94-6345.25) | 65.06 |  | 1 (0-1) | 60.16 (33.78-101.32) | 1 (1-2) | 45.50 (25.89-74.73) | 30.66 |
| Mauritania | 14954 (12943-16907) | 17600.02 (15179.44-19969.91) | 25245 (22034-28518) | 14785.45 (12855.07-16761.51) | 68.82 |  | 213 (113-358) | 260.87 (139.15-440.70) | 206 (125-314) | 128.30 (77.62-195.38) | -3.00 |
| Mauritius | 1893 (1619-2180) | 3196.81 (2728.46-3691.33) | 4679 (3980-5443) | 2802.20 (2378.68-3266.03) | 147.16 |  | 29 (19-39) | 52.61 (34.37-71.15) | 29 (18-43) | 18.30 (11.16-26.91) | 0.73 |
| Mexico | 25762 (23126-28776) | 767.30 (687.71-858.54) | 56673 (50817-63464) | 532.27 (476.70-596.63) | 119.98 |  | 346 (246-476) | 10.93 (7.75-15.03) | 761 (557-1019) | 7.28 (5.34-9.75) | 119.95 |
| Micronesia (Federated States of) | 250 (214-292) | 6153.72 (5245.06-7221.43) | 272 (232-318) | 5465.32 (4646.76-6416.68) | 9.02 |  | 2 (1-3) | 51.74 (31.26-81.83) | 2 (1-3) | 37.51 (22.39-58.42) | -14.18 |
| Monaco | 60 (51-71) | 852.93 (713.61-1003.36) | 77 (65-92) | 815.34 (683.29-966.98) | 28.00 |  | 0 (0-1) | 6.75 (3.75-10.95) | 1 (0-1) | 7.99 (4.37-13.69) | 61.42 |
| Mongolia | 6485 (5735-7251) | 7485.33 (6613.65-8392.63) | 10175 (9079-11200) | 6847.01 (6109.23-7549.77) | 56.89 |  | 105 (65-159) | 123.54 (76.21-187.23) | 150 (88-244) | 106.20 (62.30-172.96) | 42.83 |
| Montenegro | 440 (374-518) | 878.61 (746.51-1035.41) | 705 (612-822) | 735.13 (635.36-859.60) | 60.50 |  | 7 (4-10) | 13.04 (8.06-19.55) | 13 (8-20) | 14.06 (8.67-21.72) | 98.50 |
| Morocco | 33515 (30242-37370) | 2902.83 (2612.17-3245.87) | 67443 (60668-74771) | 2423.77 (2175.71-2692.02) | 101.23 |  | 588 (323-998) | 54.96 (29.75-94.19) | 1015 (560-1664) | 40.06 (21.88-65.79) | 72.59 |
| Mozambique | 48663 (41968-56205) | 11075.04 (9500.81-12857.60) | 74016 (63882-85052) | 9356.61 (8032.31-10832.56) | 52.10 |  | 535 (359-751) | 136.86 (91.24-193.05) | 752 (434-1225) | 106.94 (60.71-175.09) | 40.67 |
| Myanmar | 29584 (25398-34172) | 1657.36 (1416.30-1922.75) | 56280 (48433-65312) | 1432.57 (1231.51-1666.15) | 90.24 |  | 840 (434-1568) | 47.61 (24.34-90.11) | 1221 (640-2004) | 32.05 (16.63-52.82) | 45.39 |
| Namibia | 3494 (3114-3942) | 6776.91 (5995.27-7712.20) | 5487 (4866-6201) | 5313.13 (4685.75-6038.89) | 57.06 |  | 31 (16-59) | 68.28 (34.69-130.25) | 46 (27-74) | 48.28 (27.75-78.14) | 46.67 |
| Nauru | 20 (17-24) | 6303.73 (5304.09-7473.19) | 24 (20-28) | 5754.33 (4907.93-6826.49) | 18.33 |  | 0 (0-0) | 60.71 (37.24-91.55) | 0 (0-0) | 38.61 (18.09-62.83) | -14.07 |
| Nepal | 12011 (10956-13170) | 1772.81 (1610.75-1949.31) | 30579 (27839-34032) | 1561.62 (1418.81-1740.96) | 154.58 |  | 371 (186-717) | 59.57 (29.34-116.02) | 985 (564-1571) | 56.17 (31.98-89.58) | 165.77 |
| Netherlands | 9185 (8124-10445) | 479.66 (424.06-545.50) | 14924 (13200-16881) | 432.46 (382.91-488.63) | 62.47 |  | 59 (36-90) | 3.07 (1.86-4.73) | 127 (77-193) | 3.63 (2.20-5.52) | 116.04 |
| New Zealand | 8430 (7257-9790) | 2239.47 (1925.66-2603.00) | 18164 (15755-20844) | 2252.72 (1954.84-2583.38) | 115.47 |  | 19 (15-24) | 5.12 (3.96-6.47) | 40 (31-51) | 4.89 (3.80-6.21) | 107.28 |
| Nicaragua | 1038 (897-1205) | 875.87 (755.61-1018.68) | 2780 (2357-3272) | 703.50 (595.86-829.11) | 167.74 |  | 11 (6-18) | 9.52 (5.34-15.84) | 26 (15-42) | 6.66 (3.82-10.98) | 135.59 |
| Niger | 27505 (23648-31743) | 14077.55 (12031.48-16410.88) | 73657 (64189-85019) | 12542.39 (10856.63-14543.44) | 167.80 |  | 350 (221-554) | 193.76 (120.90-308.23) | 655 (389-1116) | 123.67 (73.55-208.99) | 87.21 |
| Nigeria | 463721 (405913-527104) | 13106.26 (11447.60-14942.60) | 665098 (585265-754017) | 10542.07 (9258.05-11979.46) | 43.43 |  | 5238 (3529-7462) | 159.34 (107.38-226.60) | 4982 (3630-6655) | 85.99 (63.43-113.91) | -4.89 |
| Niue | 10 (8-12) | 4921.56 (4095.39-5886.51) | 8 (7-9) | 4357.06 (3715.07-5058.31) | -18.84 |  | 0 (0-0) | 41.94 (26.73-60.85) | 0 (0-0) | 32.46 (19.00-50.61) | -30.93 |
| North Macedonia | 2022 (1748-2368) | 1347.42 (1164.52-1583.23) | 3577 (3116-4118) | 1125.08 (974.76-1301.45) | 76.93 |  | 35 (22-52) | 23.81 (15.09-35.31) | 58 (35-91) | 20.04 (12.09-31.74) | 63.47 |
| Northern Mariana Islands | 65 (57-73) | 7418.75 (6498.86-8310.18) | 258 (231-292) | 7038.42 (6276.65-7999.65) | 294.11 |  | 1 (0-1) | 74.77 (48.58-106.07) | 2 (1-3) | 63.63 (42.38-90.98) | 247.75 |
| Norway | 5002 (4353-5796) | 717.25 (624.40-830.73) | 5602 (4872-6473) | 581.40 (506.47-671.13) | 12.00 |  | 19 (15-24) | 2.71 (2.10-3.45) | 34 (26-44) | 3.47 (2.64-4.43) | 80.22 |
| Oman | 2082 (1769-2443) | 4503.05 (3813.05-5307.61) | 4447 (3801-5158) | 3781.68 (3217.87-4407.84) | 113.57 |  | 34 (19-57) | 77.21 (42.00-131.71) | 40 (23-64) | 38.03 (21.31-61.30) | 17.45 |
| Pakistan | 169552 (147251-194528) | 3678.42 (3182.22-4235.13) | 286594 (250926-326055) | 3285.01 (2868.02-3754.70) | 69.03 |  | 2918 (1475-5858) | 68.66 (34.18-138.57) | 4820 (3259-6876) | 61.30 (41.10-87.60) | 65.18 |
| Palau | 41 (36-47) | 5178.84 (4432.09-5924.06) | 81 (70-95) | 4775.90 (4075.02-5604.32) | 96.56 |  | 0 (0-1) | 53.39 (35.40-75.95) | 1 (0-1) | 36.22 (21.79-54.20) | 38.10 |
| Palestine | 2430 (2172-2697) | 3470.03 (3094.89-3863.63) | 5678 (5151-6342) | 3154.80 (2858.87-3530.48) | 133.69 |  | 67 (36-125) | 106.38 (56.30-197.97) | 72 (45-107) | 45.47 (27.97-68.44) | 6.12 |
| Panama | 1197 (1015-1408) | 963.14 (816.17-1134.82) | 3062 (2592-3617) | 789.58 (668.74-932.17) | 155.82 |  | 10 (6-15) | 8.18 (4.99-12.51) | 19 (11-31) | 4.90 (2.89-7.83) | 91.48 |
| Papua New Guinea | 14491 (12786-16314) | 11883.01 (10430.62-13483.31) | 33842 (29941-38437) | 10503.39 (9264.15-11974.19) | 133.54 |  | 35 (19-65) | 30.38 (16.66-55.47) | 63 (36-107) | 20.49 (11.70-34.61) | 79.16 |
| Paraguay | 3135 (2642-3736) | 1722.04 (1448.97-2055.27) | 7257 (6259-8548) | 1499.89 (1292.72-1767.77) | 131.50 |  | 23 (13-36) | 12.84 (7.38-19.86) | 66 (37-108) | 13.74 (7.72-22.52) | 184.91 |
| Peru | 19419 (16397-23603) | 2063.37 (1739.69-2510.26) | 52004 (44287-61492) | 1828.76 (1558.25-2161.41) | 167.80 |  | 229 (127-382) | 24.59 (13.70-41.05) | 547 (304-905) | 19.22 (10.69-31.78) | 139.32 |
| Philippines | 220172 (193258-251756) | 10144.23 (8882.86-11631.56) | 531001 (466506-603793) | 8532.90 (7479.09-9727.37) | 141.18 |  | 986 (648-1574) | 50.72 (33.16-81.60) | 2224 (1698-2836) | 37.21 (28.42-47.31) | 125.57 |
| Poland | 46674 (42826-50916) | 1185.12 (1086.75-1293.69) | 38957 (34344-44319) | 541.45 (477.35-616.00) | -16.53 |  | 545 (401-717) | 14.07 (10.35-18.54) | 670 (501-881) | 9.27 (6.93-12.17) | 23.07 |
| Portugal | 13463 (11379-16055) | 1000.31 (844.04-1194.70) | 20590 (17355-24399) | 867.63 (734.67-1025.43) | 52.94 |  | 140 (79-229) | 10.59 (5.97-17.35) | 133 (82-202) | 5.43 (3.34-8.21) | -4.77 |
| Puerto Rico | 3387 (2816-4007) | 995.30 (826.38-1179.34) | 5826 (4982-6852) | 824.04 (707.70-966.33) | 71.99 |  | 63 (36-101) | 18.90 (10.81-30.81) | 64 (38-100) | 8.72 (5.24-13.60) | 2.25 |
| Qatar | 158 (145-174) | 3266.34 (2988.53-3583.00) | 1248 (1152-1366) | 3250.67 (2987.17-3570.46) | 688.34 |  | 6 (4-9) | 156.08 (92.54-244.20) | 24 (15-39) | 77.39 (46.45-122.39) | 300.96 |
| Republic of Korea | 107366 (99976-114885) | 4776.92 (4432.90-5123.68) | 349316 (329183-373751) | 4095.67 (3858.86-4383.49) | 225.35 |  | 4109 (2938-5370) | 197.37 (140.27-259.64) | 5230 (3524-7386) | 62.00 (41.71-87.63) | 27.31 |
| Republic of Moldova | 17966 (14642-21886) | 4884.75 (3962.98-5975.38) | 20369 (16827-24189) | 3660.29 (3021.88-4351.04) | 13.38 |  | 94 (51-160) | 25.13 (13.66-42.61) | 76 (42-127) | 13.66 (7.54-22.83) | -19.06 |
| Romania | 95208 (84069-108861) | 3905.44 (3436.43-4482.15) | 112791 (99812-128931) | 2985.88 (2643.42-3411.25) | 18.47 |  | 871 (488-1345) | 35.64 (19.99-55.06) | 1161 (642-1859) | 30.76 (17.03-49.22) | 33.25 |
| Russian Federation | 253051 (221725-289032) | 1704.72 (1491.32-1949.59) | 283308 (249928-321720) | 1239.10 (1092.90-1407.95) | 11.96 |  | 786 (585-1036) | 5.27 (3.92-6.97) | 1502 (1131-1964) | 6.59 (4.97-8.60) | 91.22 |
| Rwanda | 10813 (9068-12632) | 5086.13 (4247.16-5973.54) | 21198 (17665-24748) | 4541.20 (3769.60-5337.01) | 96.05 |  | 268 (158-405) | 137.99 (80.75-210.04) | 346 (194-559) | 83.09 (46.55-134.17) | 28.96 |
| Saint Kitts and Nevis | 51 (43-60) | 1257.14 (1043.08-1482.60) | 60 (51-70) | 1064.84 (902.50-1250.68) | 17.29 |  | 1 (1-1) | 22.90 (13.64-35.95) | 1 (0-1) | 13.97 (8.47-21.90) | -19.50 |
| Saint Lucia | 103 (87-121) | 1242.62 (1053.29-1465.43) | 216 (185-258) | 1049.04 (896.14-1252.80) | 110.55 |  | 2 (1-3) | 21.17 (11.98-34.35) | 2 (1-3) | 9.37 (5.21-15.67) | 19.81 |
| Saint Vincent and the Grenadines | 77 (64-94) | 1133.62 (941.23-1384.50) | 131 (109-156) | 1028.58 (857.16-1228.51) | 70.11 |  | 1 (1-1) | 15.05 (9.28-22.42) | 1 (1-2) | 9.52 (5.62-15.07) | 18.03 |
| Samoa | 404 (339-500) | 6040.79 (5044.40-7497.12) | 595 (509-693) | 5320.33 (4530.93-6203.81) | 47.21 |  | 3 (2-4) | 45.02 (29.23-67.71) | 4 (3-6) | 36.21 (23.60-52.87) | 33.76 |
| San Marino | 34 (29-41) | 1017.83 (864.07-1214.41) | 66 (57-80) | 962.40 (824.87-1146.66) | 92.42 |  | 0 (0-1) | 10.42 (5.86-17.16) | 0 (0-1) | 4.96 (2.68-8.31) | 5.94 |
| Sao Tome and Principe | 624 (519-725) | 11393.69 (9453.74-13288.22) | 764 (662-884) | 9629.87 (8332.28-11196.46) | 22.37 |  | 10 (6-14) | 190.38 (120.21-276.89) | 10 (5-15) | 133.13 (67.08-206.67) | -0.47 |
| Saudi Arabia | 18528 (16442-20855) | 4626.12 (4091.56-5228.13) | 38211 (34827-41984) | 3655.31 (3311.95-4040.60) | 106.23 |  | 616 (313-1205) | 165.80 (83.08-328.34) | 581 (352-915) | 68.28 (40.60-108.82) | -5.67 |
| Senegal | 28425 (24212-32728) | 11067.16 (9388.56-12804.78) | 55630 (46705-65013) | 9361.15 (7826.77-10988.97) | 95.71 |  | 338 (225-487) | 139.52 (92.96-201.01) | 501 (287-753) | 89.99 (51.21-135.98) | 48.02 |
| Serbia | 10427 (8704-12260) | 1084.14 (900.44-1283.17) | 15137 (13068-17782) | 909.04 (784.18-1068.71) | 45.17 |  | 179 (110-269) | 19.90 (12.20-30.18) | 246 (146-375) | 14.77 (8.79-22.55) | 37.51 |
| Seychelles | 198 (170-232) | 3834.89 (3283.40-4490.16) | 316 (267-366) | 3433.53 (2899.65-3989.00) | 59.39 |  | 4 (2-5) | 72.00 (47.69-100.63) | 4 (2-6) | 43.53 (27.05-64.94) | 2.93 |
| Sierra Leone | 18613 (15809-21852) | 10758.02 (9107.13-12663.63) | 26038 (21843-30626) | 9242.88 (7725.37-10916.49) | 39.89 |  | 286 (181-424) | 173.68 (109.49-258.21) | 255 (162-377) | 96.09 (61.25-142.04) | -10.98 |
| Singapore | 7319 (6556-8092) | 4274.41 (3816.26-4737.69) | 27556 (24177-30517) | 3565.49 (3121.37-3957.62) | 276.50 |  | 90 (71-111) | 53.03 (41.40-65.69) | 222 (161-290) | 29.91 (21.59-39.03) | 146.78 |
| Slovakia | 7234 (6179-8469) | 1297.59 (1105.14-1522.94) | 10146 (8568-11892) | 1068.62 (900.15-1255.26) | 40.26 |  | 154 (95-229) | 27.37 (16.88-40.70) | 163 (94-259) | 17.23 (9.97-27.32) | 6.15 |
| Slovenia | 2695 (2284-3204) | 1250.35 (1059.72-1487.42) | 4699 (4036-5452) | 1096.98 (944.69-1269.19) | 74.39 |  | 60 (37-90) | 27.91 (17.07-41.80) | 82 (52-122) | 18.89 (11.97-28.03) | 36.35 |
| Solomon Islands | 887 (759-1028) | 9434.82 (8007.34-11050.19) | 1867 (1587-2185) | 8038.69 (6795.61-9446.58) | 110.43 |  | 6 (3-11) | 73.28 (40.22-130.57) | 12 (8-18) | 57.40 (35.91-85.75) | 94.35 |
| Somalia | 16883 (14608-19364) | 12646.34 (10886.78-14582.10) | 45592 (39546-52249) | 11150.39 (9592.26-12898.61) | 170.04 |  | 289 (178-445) | 227.14 (139.72-351.04) | 725 (456-1067) | 196.25 (121.89-291.07) | 151.14 |
| South Africa | 39482 (35093-44712) | 2400.65 (2130.34-2724.56) | 61125 (54538-69036) | 1634.57 (1454.75-1849.98) | 54.82 |  | 448 (287-699) | 29.21 (18.68-45.58) | 985 (777-1232) | 28.49 (22.42-35.72) | 120.08 |
| South Sudan | 15964 (13239-18855) | 7252.94 (5985.45-8609.03) | 17217 (14519-19910) | 6942.17 (5831.42-8061.95) | 7.85 |  | 282 (167-445) | 136.09 (80.34-213.76) | 301 (184-457) | 128.76 (78.52-194.85) | 6.77 |
| Spain | 123089 (106137-142567) | 2341.61 (2017.38-2714.30) | 186684 (161433-214986) | 2066.70 (1796.21-2367.62) | 51.67 |  | 477 (279-748) | 9.13 (5.32-14.35) | 423 (251-652) | 4.46 (2.65-6.86) | -11.29 |
| Sri Lanka | 24569 (21727-27917) | 2956.00 (2607.02-3370.32) | 61656 (55125-69312) | 2472.61 (2206.21-2785.47) | 150.95 |  | 537 (313-825) | 71.67 (42.02-109.22) | 596 (323-985) | 25.93 (14.05-42.69) | 10.96 |
| Sudan | 66405 (57811-77157) | 8674.37 (7508.94-10132.82) | 94661 (83296-108041) | 6719.09 (5886.73-7693.84) | 42.55 |  | 994 (478-1933) | 142.27 (66.57-281.12) | 897 (464-1662) | 68.82 (35.29-129.03) | -9.74 |
| Suriname | 265 (220-316) | 1339.78 (1113.09-1600.42) | 570 (481-667) | 1091.21 (919.04-1278.60) | 115.25 |  | 4 (2-7) | 20.72 (11.31-35.00) | 6 (3-11) | 12.09 (6.40-20.68) | 55.17 |
| Sweden | 7438 (6437-8683) | 487.23 (421.93-568.75) | 10343 (8941-11943) | 491.02 (425.71-565.63) | 39.06 |  | 43 (31-57) | 2.77 (2.03-3.72) | 62 (45-85) | 2.89 (2.08-3.94) | 45.36 |
| Switzerland | 8548 (7258-10024) | 864.70 (735.42-1012.54) | 13546 (11342-16118) | 808.96 (680.87-959.04) | 58.46 |  | 57 (36-87) | 5.72 (3.57-8.68) | 91 (54-141) | 5.26 (3.12-8.10) | 59.39 |
| Syrian Arab Republic | 18030 (16097-20225) | 4747.65 (4231.38-5336.96) | 43802 (39793-48332) | 4265.93 (3860.29-4725.90) | 142.94 |  | 270 (155-443) | 77.47 (44.39-127.42) | 444 (267-693) | 52.61 (31.10-82.13) | 64.71 |
| Taiwan (Province of China) | 159090 (149397-169855) | 12095.21 (11309.03-12948.23) | 372451 (347225-396624) | 9264.95 (8633.65-9867.54) | 134.11 |  | 1229 (931-1519) | 106.45 (79.78-132.41) | 2203 (1597-2866) | 55.98 (40.73-72.78) | 79.29 |
| Tajikistan | 5415 (4606-6412) | 2653.72 (2255.46-3146.40) | 9153 (7757-10648) | 2412.06 (2036.18-2818.18) | 69.03 |  | 62 (36-99) | 30.61 (18.05-48.98) | 73 (40-123) | 20.78 (11.21-35.23) | 18.61 |
| Thailand | 128421 (116967-140239) | 5014.05 (4549.85-5495.53) | 415509 (375892-458145) | 4299.68 (3888.10-4743.63) | 223.55 |  | 1777 (1170-2558) | 72.19 (47.31-104.08) | 4203 (2593-6308) | 43.82 (27.08-65.71) | 136.49 |
| Timor-Leste | 818 (679-962) | 5004.17 (4128.23-5913.61) | 3257 (2747-3867) | 4265.03 (3578.16-5099.44) | 297.97 |  | 8 (4-17) | 54.20 (25.50-110.19) | 31 (16-57) | 44.19 (22.14-81.45) | 275.89 |
| Togo | 9690 (8176-11673) | 10913.11 (9162.40-13218.24) | 23607 (20406-27852) | 9007.32 (7720.24-10684.33) | 143.62 |  | 92 (58-135) | 110.75 (69.64-163.59) | 203 (119-316) | 84.86 (49.25-132.70) | 120.46 |
| Tokelau | 7 (6-8) | 6039.66 (5126.50-7196.43) | 7 (6-8) | 5187.43 (4458.47-6006.39) | 1.69 |  | 0 (0-0) | 51.44 (29.84-84.81) | 0 (0-0) | 35.72 (22.92-52.17) | -17.28 |
| Tonga | 348 (306-395) | 7914.80 (6942.24-8997.06) | 480 (413-557) | 7170.45 (6165.31-8313.33) | 38.10 |  | 6 (4-9) | 133.85 (89.19-202.10) | 6 (4-9) | 87.93 (55.68-131.35) | 2.86 |
| Trinidad and Tobago | 790 (663-938) | 1043.79 (874.00-1243.15) | 1561 (1325-1834) | 863.72 (731.94-1016.06) | 97.70 |  | 10 (6-15) | 13.40 (8.01-21.37) | 10 (6-16) | 5.70 (3.36-9.29) | 4.83 |
| Tunisia | 16226 (14503-18107) | 4032.58 (3584.68-4528.64) | 38095 (34169-42704) | 3352.44 (3001.32-3766.89) | 134.77 |  | 138 (77-227) | 39.47 (21.75-66.74) | 240 (132-400) | 22.74 (12.39-38.09) | 74.51 |
| Turkey | 95678 (87585-104205) | 3789.12 (3460.94-4135.62) | 234924 (215142-255204) | 2855.86 (2610.06-3108.81) | 145.54 |  | 1706 (1114-2538) | 71.32 (46.40-106.34) | 3258 (2143-4553) | 42.52 (27.90-59.54) | 90.90 |
| Turkmenistan | 3313 (2790-3866) | 2372.10 (1994.11-2776.34) | 6135 (5156-7149) | 2101.63 (1762.84-2459.37) | 85.18 |  | 53 (32-82) | 39.11 (23.72-60.79) | 82 (47-132) | 28.82 (16.52-46.76) | 53.95 |
| Tuvalu | 39 (33-46) | 7025.62 (5917.48-8383.17) | 54 (45-64) | 6033.21 (5089.05-7272.62) | 37.60 |  | 0 (0-0) | 57.84 (34.06-93.40) | 0 (0-0) | 39.25 (25.43-58.24) | 7.93 |
| Uganda | 39067 (33962-44602) | 7862.94 (6801.56-9021.18) | 63432 (55995-72914) | 6134.95 (5391.74-7087.12) | 62.37 |  | 619 (397-897) | 132.77 (84.02-193.62) | 805 (457-1268) | 83.34 (46.45-131.88) | 30.17 |
| Ukraine | 56565 (49065-65892) | 901.25 (780.60-1051.16) | 62763 (54609-71823) | 841.49 (731.88-963.46) | 10.96 |  | 311 (233-405) | 4.88 (3.65-6.38) | 291 (194-419) | 3.89 (2.60-5.61) | -6.38 |
| United Arab Emirates | 252 (226-281) | 1144.41 (1021.13-1279.81) | 2211 (1983-2459) | 1208.14 (1075.33-1349.35) | 775.72 |  | 16 (10-25) | 79.47 (49.09-121.64) | 70 (43-109) | 61.69 (37.99-97.11) | 329.67 |
| United Kingdom | 73366 (63788-85149) | 818.83 (711.91-950.56) | 102816 (90271-118057) | 831.55 (731.76-953.44) | 40.14 |  | 211 (161-270) | 2.34 (1.79-3.00) | 703 (513-924) | 5.49 (4.01-7.21) | 233.20 |
| United Republic of Tanzania | 53598 (46833-60751) | 6275.84 (5450.27-7162.94) | 95885 (84493-108051) | 5112.46 (4487.14-5788.57) | 78.90 |  | 916 (579-1299) | 114.55 (71.42-164.13) | 1408 (821-2206) | 79.38 (45.97-124.35) | 53.69 |
| United States of America | 142679 (124352-165504) | 456.13 (397.50-529.12) | 252965 (221307-292425) | 443.39 (388.01-512.35) | 77.30 |  | 1111 (861-1421) | 3.54 (2.74-4.53) | 2805 (2177-3505) | 4.92 (3.82-6.14) | 152.42 |
| United States Virgin Islands | 68 (58-79) | 1011.18 (860.91-1182.68) | 159 (134-191) | 845.48 (709.46-1015.78) | 134.27 |  | 1 (1-2) | 16.29 (8.81-27.98) | 1 (1-2) | 6.99 (3.82-11.97) | 27.48 |
| Uruguay | 1601 (1372-1845) | 431.23 (369.17-497.18) | 2358 (2052-2795) | 453.96 (396.02-536.66) | 47.25 |  | 36 (20-60) | 9.86 (5.48-16.22) | 38 (23-58) | 7.12 (4.42-10.94) | 3.31 |
| Uzbekistan | 52847 (44156-61250) | 6107.02 (5103.84-7073.92) | 98662 (84496-115591) | 5278.89 (4504.19-6203.59) | 86.69 |  | 248 (147-385) | 28.94 (17.09-45.08) | 362 (211-572) | 19.76 (11.42-31.47) | 46.13 |
| Vanuatu | 346 (293-407) | 7701.68 (6485.95-9106.60) | 844 (712-987) | 6689.86 (5599.73-7876.81) | 143.91 |  | 3 (1-6) | 68.00 (31.85-147.67) | 6 (3-11) | 49.07 (26.83-91.26) | 104.39 |
| Venezuela (Bolivarian Republic of) | 8821 (7459-10402) | 1181.19 (997.39-1394.70) | 27082 (23217-31647) | 1037.73 (888.37-1214.18) | 207.03 |  | 74 (45-112) | 9.98 (6.12-15.17) | 132 (75-223) | 5.20 (2.97-8.75) | 78.66 |
| Viet Nam | 321626 (284542-361107) | 9536.16 (8412.41-10736.02) | 607625 (533725-687284) | 7823.91 (6848.87-8879.38) | 88.92 |  | 3802 (2298-6187) | 118.95 (71.27-195.71) | 5086 (3047-7613) | 69.73 (41.70-104.78) | 33.76 |
| Yemen | 32673 (29004-36830) | 9147.47 (8070.27-10394.90) | 74954 (66570-83530) | 7279.60 (6442.20-8152.97) | 129.41 |  | 534 (255-1075) | 166.32 (76.78-342.66) | 673 (372-1121) | 73.29 (39.62-123.53) | 25.91 |
| Zambia | 12338 (10696-14039) | 6096.98 (5254.59-6981.21) | 21294 (18724-24356) | 4539.94 (3966.88-5226.40) | 72.59 |  | 327 (216-477) | 171.80 (112.69-250.30) | 442 (246-694) | 101.35 (55.47-160.35) | 34.94 |
| Zimbabwe | 50769 (43007-59512) | 15977.47 (13453.30-18843.25) | 73311 (62148-85762) | 14527.05 (12265.45-17117.59) | 44.40 |  | 274 (178-397) | 92.91 (60.13-134.97) | 361 (194-580) | 77.99 (41.56-125.97) | 31.65 |

Table S7 The AAPCs of age-standardized incidence, prevalence, mortality, and DALYs of total burden related to hepatitis B in adults aged ≥65 years from 1990 to 2021 in 204 countries and territories

| location | Incidence | |  | DALYs | |  | Prevalence | |  | Mortality | |
| --- | --- | --- | --- | --- | --- | --- | --- | --- | --- | --- | --- |
|  | Incidence AAPC (95% CI) | P value |  | DALYs AAPC (95% CI) | P value |  | Prevalence AAPC (95% CI) | P value |  | Mortality AAPC (95% CI) | P value |
| Afghanistan | -0.27  (-0.29 to -0.26) | <0.001 |  | -2.00  (-2.03 to -1.97) | <0.001 |  | -0.57  (-0.58 to -0.55) | <0.001 |  | -1.93  (-1.96 to -1.91) | <0.001 |
| Albania | -0.18  (-0.21 to -0.14) | <0.001 |  | -2.81  (-2.92 to -2.72) | <0.001 |  | -0.43  (-0.44 to -0.43) | <0.001 |  | -2.70  (-2.80 to -2.62) | <0.001 |
| Algeria | -0.09  (-0.11 to -0.08) | <0.001 |  | -1.57  (-1.59 to -1.55) | <0.001 |  | -0.26  (-0.27 to -0.26) | <0.001 |  | -1.55  (-1.57 to -1.52) | <0.001 |
| American Samoa | -0.22  (-0.30 to -0.15) | <0.001 |  | -0.05  (-0.14 to 0.04) | 0.230 |  | -0.38  (-0.43 to -0.33) | <0.001 |  | 0.00  (-0.09 to 0.09) | 0.948 |
| Andorra | -0.37  (-0.41 to -0.33) | <0.001 |  | -1.19  (-1.26 to -1.13) | <0.001 |  | -0.36  (-0.37 to -0.36) | <0.001 |  | -1.10  (-1.17 to -1.04) | <0.001 |
| Angola | -0.30  (-0.35 to -0.25) | <0.001 |  | -2.22  (-2.25 to -2.19) | <0.001 |  | -0.70  (-0.70 to -0.69) | <0.001 |  | -2.19  (-2.22 to -2.15) | <0.001 |
| Antigua and Barbuda | -0.08  (-0.09 to -0.07) | <0.001 |  | -1.51  (-1.82 to -1.09) | <0.001 |  | -0.39  (-0.41 to -0.38) | <0.001 |  | -1.45  (-1.74 to -1.04) | <0.001 |
| Argentina | 0.02  (-0.03 to 0.04) | 0.210 |  | -1.27  (-1.41 to -1.15) | <0.001 |  | 0.11  (0.10 to 0.12) | <0.001 |  | -1.39  (-1.53 to -1.23) | <0.001 |
| Armenia | -0.12  (-0.13 to -0.11) | <0.001 |  | -0.82  (-1.09 to -0.54) | <0.001 |  | -0.35  (-0.36 to -0.35) | <0.001 |  | -0.48  (-0.83 to -0.14) | 0.006 |
| Australia | -0.04  (-0.07 to -0.01) | 0.008 |  | -0.18  (-0.29 to -0.06) | 0.009 |  | -0.29  (-0.30 to -0.28) | <0.001 |  | 0.00  (-0.10 to 0.10) | 0.938 |
| Austria | -0.19  (-0.28 to -0.14) | <0.001 |  | -1.45  (-1.53 to -1.34) | <0.001 |  | -0.22  (-0.23 to -0.21) | <0.001 |  | -1.41  (-1.50 to -1.33) | <0.001 |
| Azerbaijan | 0.07  (0.07 to 0.08) | <0.001 |  | -0.26  (-0.33 to -0.19) | <0.001 |  | -0.09  (-0.10 to -0.08) | <0.001 |  | -0.39  (-0.46 to -0.34) | <0.001 |
| Bahamas | -0.36  (-0.40 to -0.33) | <0.001 |  | -1.53  (-1.68 to -1.38) | <0.001 |  | -0.38  (-0.40 to -0.36) | <0.001 |  | -1.44  (-1.59 to -1.30) | <0.001 |
| Bahrain | -0.07  (-0.08 to -0.06) | <0.001 |  | -2.90  (-3.01 to -2.79) | <0.001 |  | -0.10  (-0.10 to -0.09) | <0.001 |  | -2.71  (-2.84 to -2.61) | <0.001 |
| Bangladesh | -0.25  (-0.28 to -0.22) | <0.001 |  | -2.19  (-2.28 to -2.10) | <0.001 |  | -0.70  (-0.71 to -0.68) | <0.001 |  | -1.80  (-1.91 to -1.69) | <0.001 |
| Barbados | -0.07  (-0.09 to -0.05) | <0.001 |  | -1.45  (-1.59 to -1.29) | <0.001 |  | -0.30  (-0.31 to -0.29) | <0.001 |  | -1.45  (-1.59 to -1.29) | <0.001 |
| Belarus | -0.94  (-0.99 to -0.90) | <0.001 |  | -0.03  (-0.23 to 0.17) | 0.808 |  | -0.88  (-0.91 to -0.85) | <0.001 |  | -0.04  (-0.25 to 0.15) | 0.707 |
| Belgium | -0.01  (-0.05 to 0.03) | 0.664 |  | -0.90  (-1.00 to -0.78) | <0.001 |  | -0.35  (-0.36 to -0.34) | <0.001 |  | -1.04  (-1.17 to -0.90) | <0.001 |
| Belize | -0.25  (-0.27 to -0.24) | <0.001 |  | -0.83  (-1.06 to -0.63) | <0.001 |  | -0.57  (-0.59 to -0.55) | <0.001 |  | -0.87  (-1.09 to -0.67) | <0.001 |
| Benin | -0.34  (-0.45 to -0.28) | <0.001 |  | -1.80  (-1.84 to -1.77) | <0.001 |  | -0.63  (-0.66 to -0.60) | <0.001 |  | -1.75  (-1.78 to -1.72) | <0.001 |
| Bermuda | -0.37  (-0.43 to -0.33) | <0.001 |  | -3.58  (-3.74 to -3.45) | <0.001 |  | -0.38  (-0.41 to -0.37) | <0.001 |  | -3.53  (-3.68 to -3.41) | <0.001 |
| Bhutan | -0.26  (-0.28 to -0.24) | <0.001 |  | -0.67  (-0.69 to -0.64) | <0.001 |  | -1.00  (-1.00 to -0.99) | <0.001 |  | -0.47  (-0.48 to -0.44) | <0.001 |
| Bolivia (Plurinational State of) | -0.02  (-0.03 to -0.01) | <0.001 |  | -1.08  (-1.11 to -1.05) | <0.001 |  | -0.00  (-0.02 to 0.01) | 0.467 |  | -1.00  (-1.03 to -0.97) | <0.001 |
| Bosnia and Herzegovina | -0.26  (-0.29 to -0.23) | <0.001 |  | -1.55  (-1.64 to -1.47) | <0.001 |  | -0.74  (-0.75 to -0.73) | <0.001 |  | -1.42  (-1.51 to -1.32) | <0.001 |
| Botswana | -0.61  (-0.64 to -0.58) | <0.001 |  | -2.22  (-2.28 to -2.16) | <0.001 |  | -1.02  (-1.05 to -0.99) | <0.001 |  | -2.27  (-2.33 to -2.20) | <0.001 |
| Brazil | -2.57  (-2.60 to -2.53) | <0.001 |  | -1.47  (-1.55 to -1.39) | <0.001 |  | -1.47  (-1.49 to -1.46) | <0.001 |  | -1.54  (-1.62 to -1.47) | <0.001 |
| Brunei Darussalam | -0.23  (-0.26 to -0.21) | <0.001 |  | -2.25  (-2.39 to -2.15) | <0.001 |  | -0.31  (-0.33 to -0.30) | <0.001 |  | -2.15  (-2.29 to -2.04) | <0.001 |
| Bulgaria | -0.45  (-0.48 to -0.41) | <0.001 |  | -0.78  (-0.93 to -0.64) | <0.001 |  | -0.77  (-0.78 to -0.75) | <0.001 |  | -0.96  (-1.12 to -0.84) | <0.001 |
| Burkina Faso | -0.32  (-0.34 to -0.29) | <0.001 |  | -1.45  (-1.50 to -1.40) | <0.001 |  | -0.51  (-0.52 to -0.50) | <0.001 |  | -1.44  (-1.50 to -1.38) | <0.001 |
| Burundi | 0.19  (0.06 to 0.29) | 0.005 |  | -1.39  (-1.41 to -1.36) | <0.001 |  | -0.12  (-0.15 to -0.09) | <0.001 |  | -1.32  (-1.34 to -1.29) | <0.001 |
| Cabo Verde | -0.60  (-0.64 to -0.57) | <0.001 |  | -0.21  (-0.42 to 0.02) | 0.070 |  | -0.78  (-0.79 to -0.77) | <0.001 |  | -0.17  (-0.38 to 0.06) | 0.107 |
| Cambodia | -0.57  (-0.59 to -0.54) | <0.001 |  | -1.97  (-1.99 to -1.95) | <0.001 |  | -0.70  (-0.71 to -0.68) | <0.001 |  | -1.87  (-1.89 to -1.84) | <0.001 |
| Cameroon | -0.20  (-0.25 to -0.16) | <0.001 |  | -1.44  (-1.47 to -1.42) | <0.001 |  | -0.67  (-0.68 to -0.67) | <0.001 |  | -1.44  (-1.47 to -1.41) | <0.001 |
| Canada | -0.11  (-0.21 to -0.01) | 0.032 |  | -0.45  (-0.52 to -0.38) | <0.001 |  | -0.40  (-0.42 to -0.38) | <0.001 |  | -0.29  (-0.36 to -0.23) | <0.001 |
| Central African Republic | -0.24  (-0.30 to -0.19) | <0.001 |  | -0.88  (-0.90 to -0.86) | <0.001 |  | -0.39  (-0.40 to -0.38) | <0.001 |  | -0.86  (-0.88 to -0.85) | <0.001 |
| Chad | -0.14  (-0.17 to -0.11) | <0.001 |  | -0.44  (-0.48 to -0.41) | <0.001 |  | -0.38  (-0.40 to -0.35) | <0.001 |  | -0.43  (-0.46 to -0.39) | <0.001 |
| Chile | -0.02  (-0.05 to 0.00) | 0.068 |  | -1.68  (-1.80 to -1.47) | <0.001 |  | 0.08  (0.08 to 0.09) | <0.001 |  | -1.72  (-1.87 to -1.55) | <0.001 |
| China | -1.77  (-1.78 to -1.75) | <0.001 |  | -2.36  (-2.41 to -2.30) | <0.001 |  | -0.99  (-1.00 to -0.98) | <0.001 |  | -2.27  (-2.33 to -2.20) | <0.001 |
| Colombia | -0.70  (-0.80 to -0.64) | <0.001 |  | -1.29  (-1.47 to -1.05) | <0.001 |  | -0.67  (-0.68 to -0.66) | <0.001 |  | -1.24  (-1.41 to -1.02) | <0.001 |
| Comoros | -0.33  (-0.36 to -0.30) | <0.001 |  | -1.11  (-1.14 to -1.07) | <0.001 |  | -0.64  (-0.66 to -0.61) | <0.001 |  | -1.02  (-1.06 to -0.99) | <0.001 |
| Congo | -0.47  (-0.56 to -0.41) | <0.001 |  | -1.72  (-1.75 to -1.69) | <0.001 |  | -0.80  (-0.80 to -0.79) | <0.001 |  | -1.69  (-1.72 to -1.66) | <0.001 |
| Cook Islands | -0.55  (-0.59 to -0.49) | <0.001 |  | -0.75  (-0.88 to -0.64) | <0.001 |  | -0.70  (-0.76 to -0.65) | <0.001 |  | -0.80  (-0.94 to -0.70) | <0.001 |
| Costa Rica | -0.60  (-0.73 to -0.51) | <0.001 |  | -0.05  (-0.34 to 0.37) | 0.760 |  | -0.66  (-0.71 to -0.64) | <0.001 |  | -0.06  (-0.37 to 0.39) | 0.707 |
| Coted'Ivoire | -0.60  (-0.66 to -0.56) | <0.001 |  | -1.87  (-1.90 to -1.84) | <0.001 |  | -0.66  (-0.67 to -0.64) | <0.001 |  | -1.83  (-1.87 to -1.80) | <0.001 |
| Croatia | 0.18  (0.11 to 0.23) | <0.001 |  | -1.70  (-1.78 to -1.59) | <0.001 |  | -0.39  (-0.41 to -0.38) | <0.001 |  | -1.69  (-1.78 to -1.57) | <0.001 |
| Cuba | -0.61  (-0.69 to -0.51) | <0.001 |  | -1.43  (-1.62 to -1.19) | <0.001 |  | -0.54  (-0.57 to -0.51) | <0.001 |  | -1.68  (-1.88 to -1.44) | <0.001 |
| Cyprus | -0.28  (-0.30 to -0.26) | <0.001 |  | -2.29  (-2.37 to -2.20) | <0.001 |  | -0.66  (-0.67 to -0.65) | <0.001 |  | -2.54  (-2.61 to -2.45) | <0.001 |
| Czechia | -0.06  (-0.12 to 0.02) | 0.086 |  | -1.18  (-1.27 to -1.09) | <0.001 |  | -0.68  (-0.70 to -0.65) | <0.001 |  | -1.25  (-1.34 to -1.16) | <0.001 |
| Democratic People's Republic of Korea | -0.39  (-0.46 to -0.31) | <0.001 |  | -1.49  (-1.53 to -1.47) | <0.001 |  | -0.46  (-0.48 to -0.43) | <0.001 |  | -1.53  (-1.56 to -1.51) | <0.001 |
| Democratic Republic of the Congo | -0.19  (-0.22 to -0.16) | <0.001 |  | -0.87  (-0.91 to -0.83) | <0.001 |  | -0.42  (-0.43 to -0.41) | <0.001 |  | -0.90  (-0.94 to -0.86) | <0.001 |
| Denmark | 0.03  (-0.10 to 0.14) | 0.261 |  | 1.27  (1.07 to 1.50) | <0.001 |  | -0.04  (-0.06 to -0.02) | <0.001 |  | 1.29  (1.10 to 1.51) | <0.001 |
| Djibouti | -0.26  (-0.29 to -0.21) | <0.001 |  | -0.64  (-0.67 to -0.61) | <0.001 |  | -0.33  (-0.35 to -0.30) | <0.001 |  | -0.60  (-0.62 to -0.58) | <0.001 |
| Dominica | -0.21  (-0.24 to -0.19) | <0.001 |  | -1.42  (-1.48 to -1.35) | <0.001 |  | -0.52  (-0.53 to -0.51) | <0.001 |  | -1.41  (-1.47 to -1.35) | <0.001 |
| Dominican Republic | -0.34  (-0.43 to -0.30) | <0.001 |  | -1.24  (-1.36 to -1.09) | <0.001 |  | -0.59  (-0.60 to -0.58) | <0.001 |  | -1.55  (-1.70 to -1.41) | <0.001 |
| Ecuador | -0.07  (-0.07 to -0.07) | <0.001 |  | -1.44  (-1.62 to -1.29) | <0.001 |  | -0.06  (-0.07 to -0.05) | <0.001 |  | -1.48  (-1.67 to -1.30) | <0.001 |
| Egypt | -0.89  (-0.95 to -0.85) | <0.001 |  | -2.91  (-3.02 to -2.79) | <0.001 |  | -1.01  (-1.03 to -0.99) | <0.001 |  | -3.27  (-3.40 to -3.17) | <0.001 |
| El Salvador | -0.76  (-0.89 to -0.68) | <0.001 |  | -1.29  (-1.49 to -0.99) | <0.001 |  | -0.84  (-0.89 to -0.81) | <0.001 |  | -1.24  (-1.42 to -0.96) | <0.001 |
| Equatorial Guinea | -0.15  (-0.17 to -0.13) | <0.001 |  | -2.20  (-2.24 to -2.16) | <0.001 |  | -1.24  (-1.25 to -1.23) | <0.001 |  | -2.06  (-2.10 to -2.02) | <0.001 |
| Eritrea | -0.29  (-0.39 to -0.23) | <0.001 |  | -1.19  (-1.21 to -1.17) | <0.001 |  | -0.51  (-0.54 to -0.49) | <0.001 |  | -1.07  (-1.09 to -1.04) | <0.001 |
| Estonia | -0.92  (-0.98 to -0.87) | <0.001 |  | 0.61  (0.38 to 0.86) | 0.001 |  | -0.89  (-0.93 to -0.86) | <0.001 |  | 0.52  (0.29 to 0.72) | <0.001 |
| Eswatini | -0.60  (-0.77 to -0.44) | <0.001 |  | -0.75  (-0.80 to -0.69) | <0.001 |  | -0.95  (-0.96 to -0.94) | <0.001 |  | -0.84  (-0.88 to -0.78) | <0.001 |
| Ethiopia | -0.45  (-0.48 to -0.43) | <0.001 |  | -1.90  (-1.91 to -1.87) | <0.001 |  | -0.54  (-0.57 to -0.52) | <0.001 |  | -1.75  (-1.77 to -1.72) | <0.001 |
| Fiji | -0.21  (-0.29 to -0.16) | <0.001 |  | -0.73  (-0.86 to -0.60) | <0.001 |  | -0.29  (-0.33 to -0.24) | <0.001 |  | -0.62  (-0.73 to -0.50) | <0.001 |
| Finland | -0.22  (-0.28 to -0.17) | <0.001 |  | 0.91  (0.76 to 1.05) | <0.001 |  | -0.30  (-0.31 to -0.30) | <0.001 |  | 0.77  (0.63 to 0.93) | <0.001 |
| France | 0.06  (0.03 to 0.09) | 0.001 |  | -1.11  (-1.17 to -1.05) | <0.001 |  | -0.30  (-0.31 to -0.30) | <0.001 |  | -1.05  (-1.12 to -0.99) | <0.001 |
| Gabon | -0.15  (-0.17 to -0.14) | <0.001 |  | -1.64  (-1.67 to -1.61) | <0.001 |  | -0.67  (-0.69 to -0.66) | <0.001 |  | -1.66  (-1.69 to -1.63) | <0.001 |
| Gambia | -0.16  (-0.18 to -0.14) | <0.001 |  | -0.58  (-0.69 to -0.45) | <0.001 |  | -0.72  (-0.73 to -0.70) | <0.001 |  | -0.52  (-0.62 to -0.42) | <0.001 |
| Georgia | -0.09  (-0.12 to -0.07) | <0.001 |  | -1.51  (-1.81 to -1.24) | <0.001 |  | -0.33  (-0.33 to -0.32) | <0.001 |  | -1.41  (-1.80 to -1.09) | <0.001 |
| Germany | 0.38  (0.37 to 0.38) | <0.001 |  | -0.92  (-0.99 to -0.85) | <0.001 |  | -0.11  (-0.12 to -0.10) | <0.001 |  | -0.95  (-1.04 to -0.86) | <0.001 |
| Ghana | -0.31  (-0.36 to -0.27) | <0.001 |  | -1.07  (-1.12 to -1.02) | <0.001 |  | -0.66  (-0.68 to -0.65) | <0.001 |  | -0.95  (-1.00 to -0.90) | <0.001 |
| Greece | -0.25  (-0.26 to -0.23) | <0.001 |  | 0.29  (0.10 to 0.46) | 0.003 |  | -0.48  (-0.48 to -0.48) | <0.001 |  | 0.16  (-0.03 to 0.32) | 0.084 |
| Greenland | -0.17  (-0.19 to -0.15) | <0.001 |  | -0.04  (-0.17 to 0.06) | 0.394 |  | -0.25  (-0.26 to -0.24) | <0.001 |  | 0.00  (-0.15 to 0.12) | 0.945 |
| Grenada | -0.56  (-0.62 to -0.50) | <0.001 |  | -0.69  (-0.91 to -0.46) | <0.001 |  | -0.72  (-0.74 to -0.71) | <0.001 |  | -0.64  (-0.93 to -0.34) | <0.001 |
| Guam | -0.20  (-0.25 to -0.14) | <0.001 |  | -2.14  (-2.35 to -1.98) | <0.001 |  | -0.38  (-0.44 to -0.33) | <0.001 |  | -2.33  (-2.61 to -2.09) | <0.001 |
| Guatemala | -0.62  (-0.72 to -0.52) | <0.001 |  | -0.97  (-1.23 to -0.66) | <0.001 |  | -0.84  (-0.86 to -0.83) | <0.001 |  | -1.02  (-1.29 to -0.67) | <0.001 |
| Guinea | -0.27  (-0.31 to -0.23) | <0.001 |  | -1.02  (-1.06 to -0.99) | <0.001 |  | -0.37  (-0.39 to -0.36) | <0.001 |  | -1.02  (-1.06 to -0.99) | <0.001 |
| Guinea-Bissau | -0.53  (-0.63 to -0.44) | <0.001 |  | -1.77  (-1.81 to -1.73) | <0.001 |  | -0.52  (-0.56 to -0.48) | <0.001 |  | -1.67  (-1.71 to -1.64) | <0.001 |
| Guyana | -0.57  (-0.62 to -0.52) | <0.001 |  | -1.96  (-2.24 to -1.55) | <0.001 |  | -0.77  (-0.79 to -0.74) | <0.001 |  | -2.14  (-2.41 to -1.75) | <0.001 |
| Haiti | -0.39  (-0.41 to -0.37) | <0.001 |  | -1.15  (-1.18 to -1.13) | <0.001 |  | -0.53  (-0.54 to -0.52) | <0.001 |  | -1.13  (-1.16 to -1.11) | <0.001 |
| Honduras | -0.69  (-0.73 to -0.66) | <0.001 |  | 0.23  (0.18 to 0.29) | <0.001 |  | -0.70  (-0.74 to -0.67) | <0.001 |  | 0.21  (0.16 to 0.26) | <0.001 |
| Hungary | 0.12 (0.11 to 0.14) | <0.001 |  | -1.22  (-1.41 to -1.00) | <0.001 |  | -0.20  (-0.21 to -0.19) | <0.001 |  | -1.30  (-1.49 to -1.07) | <0.001 |
| Iceland | -0.16  (-0.24 to -0.10) | <0.001 |  | 0.96  (0.84 to 1.09) | <0.001 |  | -0.33  (-0.34 to -0.31) | <0.001 |  | 1.15  (1.03 to 1.29) | <0.001 |
| India | -0.38  (-0.39 to -0.36) | <0.001 |  | -0.46  (-0.55 to -0.35) | <0.001 |  | -0.51  (-0.51 to -0.50) | <0.001 |  | -0.35  (-0.49 to -0.20) | <0.001 |
| Indonesia | -0.30  (-0.32 to -0.28) | <0.001 |  | -0.76  (-0.79 to -0.72) | <0.001 |  | -0.38  (-0.39 to -0.37) | <0.001 |  | -0.60  (-0.64 to -0.56) | <0.001 |
| Iran (Islamic Republic of) | -1.37  (-1.40 to -1.34) | <0.001 |  | -1.88  (-1.92 to -1.84) | <0.001 |  | -1.05  (-1.07 to -1.04) | <0.001 |  | -1.83  (-1.87 to -1.79) | <0.001 |
| Iraq | -0.08  (-0.11 to -0.06) | <0.001 |  | -0.92  (-0.99 to -0.85) | <0.001 |  | -0.14  (-0.15 to -0.13) | <0.001 |  | -0.79  (-0.86 to -0.71) | <0.001 |
| Ireland | -0.06  (-0.09 to -0.03) | 0.001 |  | 0.10  (0.00 to 0.19) | 0.049 |  | -0.39  (-0.40 to -0.39) | <0.001 |  | 0.05  (-0.11 to 0.16) | 0.462 |
| Israel | -0.22  (-0.26 to -0.20) | <0.001 |  | -2.15  (-2.27 to -2.01) | <0.001 |  | -0.38  (-0.38 to -0.37) | <0.001 |  | -2.10  (-2.20 to -1.98) | <0.001 |
| Italy | -1.02  (-1.06 to -0.99) | <0.001 |  | -3.07  (-3.17 to -2.96) | <0.001 |  | -0.66  (-0.67 to -0.65) | <0.001 |  | -2.82  (-2.93 to -2.71) | <0.001 |
| Jamaica | -0.24  (-0.28 to -0.21) | <0.001 |  | -1.34  (-1.74 to -0.93) | <0.001 |  | -0.54  (-0.55 to -0.53) | <0.001 |  | -1.27  (-1.66 to -0.87) | <0.001 |
| Japan | -0.92  (-0.95 to -0.90) | <0.001 |  | -1.79  (-1.87 to -1.72) | <0.001 |  | -0.65  (-0.66 to -0.64) | <0.001 |  | -1.65  (-1.78 to -1.55) | <0.001 |
| Jordan | -0.37  (-0.39 to -0.35) | <0.001 |  | -3.55  (-3.64 to -3.47) | <0.001 |  | -0.72  (-0.74 to -0.70) | <0.001 |  | -3.44  (-3.57 to -3.34) | <0.001 |
| Kazakhstan | -0.02  (-0.03 to -0.01) | <0.001 |  | 0.36  (0.20 to 0.53) | <0.001 |  | -0.27  (-0.29 to -0.25) | <0.001 |  | 0.64  (0.47 to 0.83) | <0.001 |
| Kenya | -1.43  (-1.45 to -1.41) | <0.001 |  | -0.28  (-0.31 to -0.25) | <0.001 |  | -0.68  (-0.68 to -0.67) | <0.001 |  | -0.27  (-0.30 to -0.24) | <0.001 |
| Kiribati | -0.34  (-0.38 to -0.31) | <0.001 |  | -0.91  (-0.94 to -0.87) | <0.001 |  | -0.40  (-0.44 to -0.36) | <0.001 |  | -0.81  (-0.85 to -0.78) | <0.001 |
| Kuwait | -0.17  (-0.21 to -0.12) | <0.001 |  | -1.91  (-2.31 to -1.45) | <0.001 |  | -0.30  (-0.32 to -0.28) | <0.001 |  | -1.58  (-1.97 to -1.10) | <0.001 |
| Kyrgyzstan | -0.15  (-0.17 to -0.13) | <0.001 |  | -1.49  (-1.67 to -1.35) | <0.001 |  | -0.25  (-0.26 to -0.22) | <0.001 |  | -1.56  (-1.75 to -1.34) | <0.001 |
| Lao People's Democratic Republic | -0.40  (-0.50 to -0.28) | <0.001 |  | -1.86  (-1.89 to -1.82) | <0.001 |  | -0.66  (-0.67 to -0.65) | <0.001 |  | -1.75  (-1.79 to -1.71) | <0.001 |
| Latvia | -1.33  (-1.38 to -1.29) | <0.001 |  | -0.19  (-0.41 to 0.04) | 0.101 |  | -0.86  (-0.91 to -0.81) | <0.001 |  | -0.13  (-0.34 to 0.11) | 0.241 |
| Lebanon | -0.13  (-0.14 to -0.12) | <0.001 |  | -2.40  (-2.45 to -2.35) | <0.001 |  | -0.23  (-0.24 to -0.21) | <0.001 |  | -2.29  (-2.33 to -2.25) | <0.001 |
| Lesotho | -0.76  (-0.82 to -0.72) | <0.001 |  | -0.05  (-0.10 to -0.00) | 0.043 |  | -0.87  (-0.89 to -0.85) | <0.001 |  | -0.18  (-0.22 to -0.14) | <0.001 |
| Liberia | -0.27  (-0.33 to -0.22) | <0.001 |  | -1.22  (-1.29 to -1.17) | <0.001 |  | -0.37  (-0.38 to -0.36) | <0.001 |  | -1.20  (-1.25 to -1.16) | <0.001 |
| Libya | -0.24  (-0.29 to -0.19) | <0.001 |  | -0.98  (-1.07 to -0.90) | <0.001 |  | -0.54  (-0.54 to -0.54) | <0.001 |  | -1.00  (-1.09 to -0.92) | <0.001 |
| Lithuania | -1.07  (-1.17 to -0.98) | <0.001 |  | 0.88  (0.73 to 1.07) | <0.001 |  | -0.92  (-0.94 to -0.89) | <0.001 |  | 0.87  (0.73 to 1.06) | <0.001 |
| Luxembourg | -0.09  (-0.23 to 0.01) | 0.065 |  | -1.44  (-1.58 to -1.28) | <0.001 |  | -0.22  (-0.25 to -0.20) | <0.001 |  | -1.33  (-1.43 to -1.23) | <0.001 |
| Madagascar | -0.18  (-0.27 to -0.09) | <0.001 |  | -1.09  (-1.14 to -1.04) | <0.001 |  | -0.40  (-0.43 to -0.38) | <0.001 |  | -1.05  (-1.10 to -1.00) | <0.001 |
| Malawi | -0.30  (-0.35 to -0.26) | <0.001 |  | -0.73  (-0.76 to -0.70) | <0.001 |  | -0.46  (-0.49 to -0.40) | <0.001 |  | -0.70  (-0.73 to -0.67) | <0.001 |
| Malaysia | 0.04  (0.02 to 0.06) | <0.001 |  | -0.29  (-0.44 to -0.12) | 0.002 |  | -0.30  (-0.31 to -0.29) | <0.001 |  | -0.04  (-0.27 to 0.15) | 0.742 |
| Maldives | -0.54  (-0.56 to -0.52) | <0.001 |  | -3.43  (-3.52 to -3.34) | <0.001 |  | -0.91  (-0.93 to -0.89) | <0.001 |  | -3.22  (-3.33 to -3.12) | <0.001 |
| Mali | -0.32  (-0.35 to -0.29) | <0.001 |  | -0.59  (-0.62 to -0.56) | <0.001 |  | -0.34  (-0.36 to -0.33) | <0.001 |  | -0.55  (-0.58 to -0.51) | <0.001 |
| Malta | 0.03  (-0.00 to 0.06) | 0.068 |  | -1.19  (-1.29 to -1.09) | <0.001 |  | -0.38  (-0.39 to -0.38) | <0.001 |  | -1.25  (-1.37 to -1.16) | <0.001 |
| Marshall Islands | -0.29  (-0.40 to -0.21) | 0.001 |  | -0.97  (-1.08 to -0.87) | <0.001 |  | -0.39  (-0.45 to -0.34) | <0.001 |  | -0.89  (-0.98 to -0.80) | <0.001 |
| Mauritania | -0.37  (-0.45 to -0.31) | <0.001 |  | -2.40  (-2.45 to -2.35) | <0.001 |  | -0.55  (-0.56 to -0.55) | <0.001 |  | -2.24  (-2.27 to -2.20) | <0.001 |
| Mauritius | -0.10  (-0.15 to -0.06) | <0.001 |  | -3.41  (-3.64 to -3.18) | <0.001 |  | -0.41  (-0.43 to -0.39) | <0.001 |  | -3.44  (-3.71 to -3.14) | <0.001 |
| Mexico | -1.95  (-2.00 to -1.90) | <0.001 |  | -1.29  (-1.37 to -1.19) | <0.001 |  | -1.18  (-1.20 to -1.17) | <0.001 |  | -1.32  (-1.41 to -1.19) | <0.001 |
| Micronesia (Federated States of) | -0.28  (-0.33 to -0.25) | <0.001 |  | -1.07  (-1.09 to -1.04) | <0.001 |  | -0.36  (-0.41 to -0.32) | <0.001 |  | -1.03  (-1.05 to -1.01) | <0.001 |
| Monaco | 0.02  (-0.01 to 0.04) | 0.113 |  | 0.51  (0.47 to 0.54) | <0.001 |  | -0.14  (-0.15 to -0.12) | <0.001 |  | 0.58  (0.55 to 0.61) | <0.001 |
| Mongolia | -0.04  (-0.05 to -0.03) | <0.001 |  | -0.69  (-0.83 to -0.54) | <0.001 |  | -0.29  (-0.29 to -0.28) | <0.001 |  | -0.56  (-0.76 to -0.39) | <0.001 |
| Montenegro | -0.21  (-0.26 to -0.16) | <0.001 |  | 0.13  (0.05 to 0.20) | 0.005 |  | -0.57  (-0.59 to -0.56) | <0.001 |  | 0.25  (0.17 to 0.33) | <0.001 |
| Morocco | -0.21  (-0.22 to -0.20) | <0.001 |  | -1.02  (-1.05 to -0.99) | <0.001 |  | -0.57  (-0.58 to -0.56) | <0.001 |  | -1.00  (-1.03 to -0.96) | <0.001 |
| Mozambique | -0.38  (-0.40 to -0.36) | <0.001 |  | -0.78  (-0.81 to -0.75) | <0.001 |  | -0.54  (-0.54 to -0.53) | <0.001 |  | -0.80  (-0.84 to -0.77) | <0.001 |
| Myanmar | -0.05  (-0.09 to -0.01) | 0.020 |  | -1.41  (-1.44 to -1.37) | <0.001 |  | -0.44  (-0.47 to -0.40) | <0.001 |  | -1.29  (-1.32 to -1.26) | <0.001 |
| Namibia | -0.41  (-0.45 to -0.38) | <0.001 |  | -1.13  (-1.16 to -1.09) | <0.001 |  | -0.78  (-0.78 to -0.78) | <0.001 |  | -1.11  (-1.14 to -1.09) | <0.001 |
| Nauru | -0.40  (-0.47 to -0.34) | <0.001 |  | -1.41  (-1.43 to -1.39) | <0.001 |  | -0.31  (-0.36 to -0.26) | <0.001 |  | -1.47  (-1.49 to -1.46) | <0.001 |
| Nepal | -0.22  (-0.22 to -0.21) | <0.001 |  | -0.35  (-0.38 to -0.32) | <0.001 |  | -0.40  (-0.41 to -0.39) | <0.001 |  | -0.18  (-0.20 to -0.15) | <0.001 |
| Netherlands | 0.06  (0.02 to 0.08) | 0.002 |  | 0.44  (0.37 to 0.50) | <0.001 |  | -0.33  (-0.34 to -0.33) | <0.001 |  | 0.46  (0.39 to 0.53) | <0.001 |
| New Zealand | 0.05  (0.01 to 0.09) | 0.012 |  | -0.43  (-0.63 to -0.24) | <0.001 |  | 0.01  (0.01 to 0.02) | <0.001 |  | -0.26  (-0.46 to -0.06) | 0.010 |
| Nicaragua | -0.50  (-0.53 to -0.48) | <0.001 |  | -1.19  (-1.33 to -1.08) | <0.001 |  | -0.70  (-0.70 to -0.69) | <0.001 |  | -1.27  (-1.40 to -1.16) | <0.001 |
| Niger | -0.53  (-0.62 to -0.45) | <0.001 |  | -1.55  (-1.60 to -1.52) | <0.001 |  | -0.36  (-0.38 to -0.35) | <0.001 |  | -1.45  (-1.49 to -1.42) | <0.001 |
| Nigeria | -0.54  (-0.55 to -0.52) | <0.001 |  | -2.09  (-2.12 to -2.06) | <0.001 |  | -0.69  (-0.70 to -0.68) | <0.001 |  | -1.98  (-2.00 to -1.95) | <0.001 |
| Niue | -0.27  (-0.28 to -0.26) | <0.001 |  | -0.89  (-0.91 to -0.87) | <0.001 |  | -0.40  (-0.44 to -0.36) | <0.001 |  | -0.83  (-0.84 to -0.81) | <0.001 |
| North Macedonia | -0.11  (-0.14 to -0.09) | <0.001 |  | -0.80  (-0.89 to -0.69) | <0.001 |  | -0.58  (-0.59 to -0.58) | <0.001 |  | -0.57  (-0.64 to -0.49) | <0.001 |
| Northern Mariana Islands | -0.14  (-0.18 to -0.10) | <0.001 |  | -0.53  (-0.60 to -0.46) | <0.001 |  | -0.20  (-0.26 to -0.15) | <0.001 |  | -0.57  (-0.67 to -0.43) | <0.001 |
| Norway | -0.65  (-0.71 to -0.58) | <0.001 |  | 0.79  (0.52 to 0.97) | <0.001 |  | -0.67  (-0.69 to -0.65) | <0.001 |  | 0.91  (0.68 to 1.08) | <0.001 |
| Oman | -0.47  (-0.49 to -0.43) | <0.001 |  | -2.38  (-2.45 to -2.31) | <0.001 |  | -0.53  (-0.56 to -0.49) | <0.001 |  | -2.21  (-2.33 to -2.09) | <0.001 |
| Pakistan | -0.67  (-0.70 to -0.64) | <0.001 |  | -0.38  (-0.40 to -0.34) | <0.001 |  | -0.36  (-0.39 to -0.34) | <0.001 |  | -0.34  (-0.36 to -0.31) | <0.001 |
| Palau | -0.13  (-0.14 to -0.12) | <0.001 |  | -1.38  (-1.45 to -1.32) | <0.001 |  | -0.27  (-0.30 to -0.24) | <0.001 |  | -1.31  (-1.39 to -1.24) | <0.001 |
| Palestine | -0.10  (-0.11 to -0.08) | <0.001 |  | -2.73  (-2.83 to -2.64) | <0.001 |  | -0.29  (-0.31 to -0.28) | <0.001 |  | -2.78  (-2.86 to -2.70) | <0.001 |
| Panama | -0.75  (-0.79 to -0.72) | <0.001 |  | -1.56  (-1.65 to -1.45) | <0.001 |  | -0.63  (-0.65 to -0.62) | <0.001 |  | -1.61  (-1.70 to -1.48) | <0.001 |
| Papua New Guinea | -0.37  (-0.40 to -0.35) | <0.001 |  | -1.27  (-1.30 to -1.23) | <0.001 |  | -0.40  (-0.40 to -0.39) | <0.001 |  | -1.25  (-1.28 to -1.22) | <0.001 |
| Paraguay | -0.36  (-0.41 to -0.31) | <0.001 |  | 0.41  (0.33 to 0.51) | <0.001 |  | -0.43  (-0.45 to -0.41) | <0.001 |  | 0.39  (0.30 to 0.49) | <0.001 |
| Peru | -0.10  (-0.13 to -0.06) | <0.001 |  | -0.62  (-0.91 to -0.31) | 0.002 |  | -0.42  (-0.44 to -0.41) | <0.001 |  | -0.56  (-0.86 to -0.24) | 0.008 |
| Philippines | -0.82  (-0.83 to -0.80) | <0.001 |  | -0.74  (-0.80 to -0.66) | <0.001 |  | -0.55  (-0.57 to -0.53) | <0.001 |  | -0.87  (-0.94 to -0.77) | <0.001 |
| Poland | -3.91  (-3.95 to -3.87) | <0.001 |  | -1.08  (-1.18 to -1.00) | <0.001 |  | -2.49  (-2.52 to -2.47) | <0.001 |  | -1.37  (-1.47 to -1.28) | <0.001 |
| Portugal | -0.06  (-0.07 to -0.05) | <0.001 |  | -2.19  (-2.27 to -2.09) | <0.001 |  | -0.44  (-0.45 to -0.42) | <0.001 |  | -2.16  (-2.24 to -2.07) | <0.001 |
| Puerto Rico | -0.45  (-0.51 to -0.40) | <0.001 |  | -2.31  (-2.53 to -2.04) | <0.001 |  | -0.60  (-0.61 to -0.59) | <0.001 |  | -2.41  (-2.65 to -2.13) | <0.001 |
| Qatar | -0.03  (-0.06 to -0.00) | 0.030 |  | -2.09  (-2.45 to -1.73) | <0.001 |  | -0.01  (-0.02 to 0.01) | 0.213 |  | -2.23  (-2.64 to -1.81) | <0.001 |
| Republic of Korea | 0.24  (0.20 to 0.27) | <0.001 |  | -3.90  (-3.95 to -3.84) | <0.001 |  | -0.50  (-0.50 to -0.49) | <0.001 |  | -3.73  (-3.80 to -3.65) | <0.001 |
| Republic of Moldova | -1.09  (-1.13 to -1.05) | <0.001 |  | -1.89  (-2.12 to -1.65) | <0.001 |  | -0.91  (-0.94 to -0.87) | <0.001 |  | -1.86  (-2.08 to -1.64) | <0.001 |
| Romania | -0.54  (-0.55 to -0.52) | <0.001 |  | -0.45  (-0.59 to -0.30) | <0.001 |  | -0.86  (-0.87 to -0.86) | <0.001 |  | -0.45  (-0.58 to -0.31) | <0.001 |
| Russian Federation | -1.31  (-1.35 to -1.28) | <0.001 |  | 0.88  (0.71 to 1.07) | <0.001 |  | -1.00  (-1.02 to -0.97) | <0.001 |  | 0.73  (0.56 to 0.90) | <0.001 |
| Rwanda | -0.51  (-0.57 to -0.46) | <0.001 |  | -1.71  (-1.75 to -1.68) | <0.001 |  | -0.36  (-0.38 to -0.34) | <0.001 |  | -1.60  (-1.64 to -1.58) | <0.001 |
| Saint Kitts and Nevis | -0.26  (-0.30 to -0.21) | <0.001 |  | -1.37  (-1.56 to -1.06) | <0.001 |  | -0.53  (-0.55 to -0.51) | <0.001 |  | -1.46  (-1.64 to -1.18) | <0.001 |
| Saint Lucia | -0.33  (-0.40 to -0.28) | <0.001 |  | -2.54  (-2.70 to -2.37) | <0.001 |  | -0.53  (-0.55 to -0.51) | <0.001 |  | -2.65  (-2.83 to -2.47) | <0.001 |
| Saint Vincent and the Grenadines | -0.18  (-0.22 to -0.15) | <0.001 |  | -1.53  (-1.65 to -1.43) | <0.001 |  | -0.31  (-0.33 to -0.29) | <0.001 |  | -1.54  (-1.65 to -1.44) | <0.001 |
| Samoa | -0.27  (-0.40 to -0.14) | <0.001 |  | -0.81  (-0.83 to -0.78) | <0.001 |  | -0.43  (-0.48 to -0.40) | <0.001 |  | -0.73  (-0.75 to -0.70) | <0.001 |
| San Marino | -0.15  (-0.16 to -0.14) | <0.001 |  | -2.51  (-2.78 to -2.36) | <0.001 |  | -0.17  (-0.18 to -0.15) | <0.001 |  | -2.60  (-2.91 to -2.43) | <0.001 |
| Sao Tome and Principe | -0.16  (-0.18 to -0.14) | <0.001 |  | -1.13  (-1.16 to -1.09) | <0.001 |  | -0.54  (-0.55 to -0.52) | <0.001 |  | -1.15  (-1.21 to -1.10) | <0.001 |
| Saudi Arabia | 0.05  (0.02 to 0.06) | <0.001 |  | -2.87  (-2.91 to -2.84) | <0.001 |  | -0.76  (-0.77 to -0.76) | <0.001 |  | -2.82  (-2.86 to -2.79) | <0.001 |
| Senegal | -0.41  (-0.46 to -0.37) | <0.001 |  | -1.57  (-1.64 to -1.50) | <0.001 |  | -0.55  (-0.56 to -0.54) | <0.001 |  | -1.42  (-1.52 to -1.32) | <0.001 |
| Serbia | -0.20  (-0.27 to -0.13) | <0.001 |  | -0.88  (-0.97 to -0.78) | <0.001 |  | -0.59  (-0.63 to -0.56) | <0.001 |  | -0.89  (-1.00 to -0.79) | <0.001 |
| Seychelles | -0.21  (-0.26 to -0.15) | <0.001 |  | -1.94  (-2.26 to -1.75) | <0.001 |  | -0.35  (-0.36 to -0.34) | <0.001 |  | -1.75  (-2.08 to -1.53) | <0.001 |
| Sierra Leone | -0.34  (-0.47 to -0.18) | 0.001 |  | -1.88  (-1.92 to -1.85) | <0.001 |  | -0.47  (-0.51 to -0.42) | <0.001 |  | -1.87  (-1.92 to -1.83) | <0.001 |
| Singapore | -0.23  (-0.26 to -0.20) | <0.001 |  | -2.11  (-2.34 to -1.94) | <0.001 |  | -0.58  (-0.60 to -0.57) | <0.001 |  | -1.96  (-2.18 to -1.79) | <0.001 |
| Slovakia | -0.17  (-0.28 to -0.05) | 0.017 |  | -1.53  (-1.61 to -1.46) | <0.001 |  | -0.64  (-0.68 to -0.61) | <0.001 |  | -1.54  (-1.62 to -1.47) | <0.001 |
| Slovenia | 0.39  (0.38 to 0.40) | <0.001 |  | -1.54  (-1.78 to -1.32) | <0.001 |  | -0.42  (-0.44 to -0.40) | <0.001 |  | -1.31  (-1.49 to -1.11) | <0.001 |
| Solomon Islands | -0.68  (-0.71 to -0.66) | <0.001 |  | -0.94  (-1.02 to -0.86) | <0.001 |  | -0.54  (-0.60 to -0.50) | <0.001 |  | -0.84  (-0.91 to -0.78) | <0.001 |
| Somalia | -0.48  (-0.52 to -0.44) | <0.001 |  | -0.46  (-0.49 to -0.44) | <0.001 |  | -0.41  (-0.41 to -0.40) | <0.001 |  | -0.45  (-0.47 to -0.43) | <0.001 |
| South Africa | -1.50  (-1.59 to -1.42) | <0.001 |  | -0.01  (-0.12 to 0.13) | 0.960 |  | -1.23  (-1.25 to -1.21) | <0.001 |  | -0.10  (-0.22 to 0.04) | 0.109 |
| South Sudan | -0.26  (-0.30 to -0.21) | <0.001 |  | -0.21  (-0.24 to -0.19) | <0.001 |  | -0.14  (-0.15 to -0.14) | <0.001 |  | -0.18  (-0.20 to -0.16) | <0.001 |
| Spain | -0.24  (-0.26 to -0.23) | <0.001 |  | -2.31  (-2.35 to -2.27) | <0.001 |  | -0.40  (-0.41 to -0.39) | <0.001 |  | -2.27  (-2.32 to -2.23) | <0.001 |
| Sri Lanka | -0.22  (-0.24 to -0.21) | <0.001 |  | -3.07  (-3.19 to -2.94) | <0.001 |  | -0.57  (-0.57 to -0.56) | <0.001 |  | -3.21  (-3.36 to -3.03) | <0.001 |
| Sudan | -0.30  (-0.31 to -0.30) | <0.001 |  | -2.39  (-2.41 to -2.38) | <0.001 |  | -0.82  (-0.83 to -0.81) | <0.001 |  | -2.32  (-2.34 to -2.30) | <0.001 |
| Suriname | -0.53  (-0.58 to -0.47) | <0.001 |  | -1.55  (-1.68 to -1.36) | <0.001 |  | -0.64  (-0.67 to -0.61) | <0.001 |  | -1.64  (-1.80 to -1.47) | <0.001 |
| Sweden | 0.36  (0.33 to 0.39) | <0.001 |  | 0.29  (0.12 to 0.44) | 0.001 |  | 0.02  (0.01 to 0.03) | <0.001 |  | 0.30  (0.13 to 0.45) | 0.002 |
| Switzerland | 0.03  (-0.09 to 0.14) | 0.323 |  | -0.45  (-0.64 to -0.26) | <0.001 |  | -0.23  (-0.24 to -0.21) | <0.001 |  | -0.33  (-0.49 to -0.17) | 0.002 |
| Syrian Arab Republic | -0.17  (-0.18 to -0.15) | <0.001 |  | -1.40  (-1.47 to -1.34) | <0.001 |  | -0.34  (-0.36 to -0.33) | <0.001 |  | -1.23  (-1.33 to -1.13) | <0.001 |
| Taiwan (Province of China) | -0.45  (-0.48 to -0.43) | <0.001 |  | -2.09  (-2.30 to -1.94) | <0.001 |  | -0.86  (-0.86 to -0.85) | <0.001 |  | -2.13  (-2.31 to -1.98) | <0.001 |
| Tajikistan | -0.03  (-0.07 to 0.03) | 0.234 |  | -1.23  (-1.32 to -1.13) | <0.001 |  | -0.28  (-0.31 to -0.26) | <0.001 |  | -1.26  (-1.35 to -1.15) | <0.001 |
| Thailand | -0.19  (-0.27 to -0.12) | <0.001 |  | -1.68  (-1.77 to -1.59) | <0.001 |  | -0.49  (-0.49 to -0.48) | <0.001 |  | -1.62  (-1.72 to -1.52) | <0.001 |
| Timor-Leste | -0.40  (-0.42 to -0.39) | <0.001 |  | -0.73  (-0.78 to -0.68) | <0.001 |  | -0.50  (-0.52 to -0.47) | <0.001 |  | -0.72  (-0.78 to -0.68) | <0.001 |
| Togo | -0.43  (-0.47 to -0.40) | <0.001 |  | -0.89  (-0.93 to -0.86) | <0.001 |  | -0.60  (-0.66 to -0.55) | <0.001 |  | -0.88  (-0.94 to -0.84) | <0.001 |
| Tokelau | -0.50  (-0.60 to -0.42) | <0.001 |  | -1.17  (-1.22 to -1.12) | <0.001 |  | -0.50  (-0.54 to -0.46) | <0.001 |  | -1.19  (-1.23 to -1.16) | <0.001 |
| Tonga | -0.20  (-0.29 to -0.15) | <0.001 |  | -1.29  (-1.39 to -1.19) | <0.001 |  | -0.33  (-0.37 to -0.29) | <0.001 |  | -1.26  (-1.36 to -1.16) | <0.001 |
| Trinidad and Tobago | -0.59  (-0.62 to -0.56) | <0.001 |  | -2.73  (-2.96 to -2.51) | <0.001 |  | -0.59  (-0.63 to -0.57) | <0.001 |  | -2.82  (-3.06 to -2.59) | <0.001 |
| Tunisia | -0.35  (-0.41 to -0.31) | <0.001 |  | -1.79  (-1.82 to -1.76) | <0.001 |  | -0.60  (-0.60 to -0.59) | <0.001 |  | -1.80  (-1.84 to -1.75) | <0.001 |
| Turkey | -0.41  (-0.44 to -0.39) | <0.001 |  | -1.77  (-1.84 to -1.69) | <0.001 |  | -0.91  (-0.92 to -0.90) | <0.001 |  | -1.63  (-1.75 to -1.54) | <0.001 |
| Turkmenistan | -0.21  (-0.32 to -0.15) | <0.001 |  | -0.96  (-1.09 to -0.80) | <0.001 |  | -0.39  (-0.40 to -0.37) | <0.001 |  | -0.98  (-1.10 to -0.86) | <0.001 |
| Tuvalu | -0.31  (-0.34 to -0.30) | <0.001 |  | -1.34  (-1.35 to -1.33) | <0.001 |  | -0.52  (-0.57 to -0.47) | <0.001 |  | -1.26  (-1.27 to -1.25) | <0.001 |
| Uganda | -0.39  (-0.42 to -0.37) | <0.001 |  | -1.54  (-1.58 to -1.51) | <0.001 |  | -0.80  (-0.80 to -0.80) | <0.001 |  | -1.48  (-1.53 to -1.45) | <0.001 |
| Ukraine | -0.37  (-0.42 to -0.34) | <0.001 |  | -0.75  (-0.98 to -0.51) | <0.001 |  | -0.17  (-0.21 to -0.13) | <0.001 |  | -0.74  (-0.97 to -0.53) | <0.001 |
| United Arab Emirates | 0.35  (0.29 to 0.39) | <0.001 |  | -1.04  (-1.66 to -0.55) | 0.002 |  | 0.19  (0.17 to 0.20) | <0.001 |  | -0.88  (-1.55 to -0.33) | 0.008 |
| United Kingdom | 0.89  (0.86 to 0.92) | <0.001 |  | 2.58  (2.48 to 2.65) | <0.001 |  | 0.05  (0.05 to 0.06) | <0.001 |  | 2.75  (2.66 to 2.83) | <0.001 |
| United Republic of Tanzania | -0.24  (-0.26 to -0.21) | <0.001 |  | -1.23  (-1.28 to -1.19) | <0.001 |  | -0.66  (-0.66 to -0.65) | <0.001 |  | -1.15  (-1.21 to -1.10) | <0.001 |
| United States of America | 0.01  (-0.07 to 0.06) | 0.629 |  | 1.03  (0.97 to 1.08) | <0.001 |  | -0.10  (-0.12 to -0.09) | <0.001 |  | 1.05  (0.99 to 1.10) | <0.001 |
| United States Virgin Islands | -0.31  (-0.35 to -0.27) | <0.001 |  | -2.68  (-2.78 to -2.57) | <0.001 |  | -0.57  (-0.59 to -0.55) | <0.001 |  | -2.71  (-2.81 to -2.61) | <0.001 |
| Uruguay | 0.00  (-0.01 to 0.01) | 0.745 |  | -1.06  (-1.17 to -0.91) | <0.001 |  | 0.16  (0.14 to 0.17) | <0.001 |  | -1.13  (-1.24 to -0.98) | <0.001 |
| Uzbekistan | -0.54  (-0.61 to -0.49) | <0.001 |  | -1.28  (-1.39 to -1.17) | <0.001 |  | -0.48  (-0.50 to -0.47) | <0.001 |  | -1.22  (-1.32 to -1.13) | <0.001 |
| Vanuatu | -0.36  (-0.48 to -0.31) | <0.001 |  | -1.08  (-1.11 to -1.04) | <0.001 |  | -0.45  (-0.48 to -0.41) | <0.001 |  | -1.06  (-1.08 to -1.03) | <0.001 |
| Venezuela (Bolivarian Republic of) | -0.63  (-0.71 to -0.56) | <0.001 |  | -2.23  (-2.44 to -2.01) | <0.001 |  | -0.42  (-0.44 to -0.39) | <0.001 |  | -2.18  (-2.38 to -1.95) | <0.001 |
| Viet Nam | -0.08  (-0.11 to -0.04) | <0.001 |  | -1.73  (-1.74 to -1.71) | <0.001 |  | -0.64  (-0.64 to -0.63) | <0.001 |  | -1.69  (-1.71 to -1.66) | <0.001 |
| Yemen | -0.29  (-0.31 to -0.27) | <0.001 |  | -2.74  (-2.77 to -2.71) | <0.001 |  | -0.74  (-0.74 to -0.73) | <0.001 |  | -2.63  (-2.66 to -2.59) | <0.001 |
| Zambia | -0.37  (-0.39 to -0.35) | <0.001 |  | -1.70  (-1.75 to -1.67) | <0.001 |  | -0.94  (-0.95 to -0.94) | <0.001 |  | -1.66  (-1.70 to -1.61) | <0.001 |
| Zimbabwe | -0.32  (-0.38 to -0.27) | <0.001 |  | -0.52  (-0.57 to -0.48) | <0.001 |  | -0.32  (-0.33 to -0.31) | <0.001 |  | -0.54  (-0.59 to -0.50) | <0.001 |

Table S8 Prediction of age-standardized incidence, prevalence, mortality, and DALYs of total burden related to hepatitis B in adults aged ≥65 years from 2022 to 2030

|  |  | Incidence | Prevalence | Mortality | DALYs (Disability-Adjusted Life Years) |
| --- | --- | --- | --- | --- | --- |
| year | sex | ASR | ASR | ASR | ASR |
| 2022 | Male | 785.55 (769.01-802.09) | 4314.35 (4237.41-4391.30) | 49.04 (47.57-50.50) | 880.20 (845.60-914.79) |
| 2023 | Male | 779.40 (753.80-805.00) | 4321.51 (4204.17-4438.85) | 48.56 (46.50-50.62) | 871.63 (826.34-916.92) |
| 2024 | Male | 773.59 (736.51-810.67) | 4330.47 (4160.84-4500.10) | 48.10 (45.28-50.91) | 863.24 (803.93-922.55) |
| 2025 | Male | 768.00 (717.66-818.33) | 4340.71 (4109.27-4572.14) | 47.64 (43.95-51.33) | 854.97 (779.01-930.94) |
| 2026 | Male | 762.59 (697.44-827.73) | 4351.64 (4049.56-4653.72) | 47.18 (42.51-51.85) | 846.79 (751.91-941.68) |
| 2027 | Male | 757.50 (676.05-838.95) | 4363.63 (3982.07-4745.18) | 46.71 (40.97-52.46) | 838.70 (722.85-954.55) |
| 2028 | Male | 752.66 (653.64-851.68) | 4376.69 (3907.80-4845.58) | 46.26 (39.36-53.16) | 830.74 (692.25-969.23) |
| 2029 | Male | 748.04 (630.32-865.75) | 4390.90 (3827.29-4954.52) | 45.82 (37.70-53.94) | 822.90 (660.29-985.51) |
| 2030 | Male | 743.55 (606.08-881.02) | 4405.93 (3740.36-5071.50) | 45.38 (35.97-54.78) | 815.16 (627.06-1003.26) |
| 2022 | Female | 424.90 (416.59-433.20) | 2944.05 (2894.24-2993.86) | 20.36 (19.86-20.85) | 363.60 (353.57-373.63) |
| 2023 | Female | 422.95 (410.08-435.82) | 2958.77 (2881.98-3035.57) | 20.02 (19.31-20.73) | 358.02 (344.11-371.94) |
| 2024 | Female | 420.89 (402.23-439.55) | 2974.21 (2862.50-3085.92) | 19.69 (18.72-20.67) | 352.61 (333.71-371.52) |
| 2025 | Female | 418.74 (393.35-444.12) | 2990.70 (2837.49-3143.91) | 19.39 (18.12-20.66) | 347.46 (322.70-372.23) |
| 2026 | Female | 416.76 (383.78-449.75) | 3008.36 (2807.13-3209.58) | 19.09 (17.48-20.70) | 342.59 (311.13-374.04) |
| 2027 | Female | 414.81 (373.37-456.26) | 3026.59 (2770.49-3282.69) | 18.80 (16.81-20.79) | 337.82 (298.85-376.79) |
| 2028 | Female | 412.83 (362.21-463.45) | 3045.00 (2727.99-3362.00) | 18.51 (16.12-20.91) | 333.12 (286.00-380.23) |
| 2029 | Female | 410.79 (350.36-471.22) | 3063.93 (2680.32-3447.54) | 18.24 (15.40-21.07) | 328.52 (272.72-384.32) |
| 2030 | Female | 408.70 (337.88-479.52) | 3083.58 (2627.67-3539.49) | 17.97 (14.68-21.26) | 324.10 (259.09-389.11) |
| 2022 | Both | 589.30 (578.28-600.31) | 3573.55 (3514.40-3632.69) | 33.10 (32.24-33.96) | 599.89 (582.12-617.66) |
| 2023 | Both | 585.38 (568.24-602.52) | 3584.71 (3493.31-3676.11) | 32.69 (31.46-33.93) | 592.77 (567.81-617.74) |
| 2024 | Both | 581.58 (556.67-606.48) | 3597.04 (3463.94-3730.14) | 32.29 (30.58-34.00) | 585.75 (551.52-619.98) |
| 2025 | Both | 577.80 (543.90-611.71) | 3610.35 (3427.85-3792.86) | 31.90 (29.65-34.16) | 578.86 (533.76-623.96) |
| 2026 | Both | 574.16 (530.12-618.21) | 3624.38 (3385.03-3863.73) | 31.52 (28.65-34.38) | 572.09 (514.65-629.53) |
| 2027 | Both | 570.68 (515.38-625.99) | 3639.15 (3335.25-3943.05) | 31.13 (27.58-34.68) | 565.38 (494.19-636.56) |
| 2028 | Both | 567.31 (499.81-634.81) | 3654.46 (3279.22-4029.70) | 30.75 (26.47-35.02) | 558.72 (472.69-644.75) |
| 2029 | Both | 564.01 (483.50-644.53) | 3670.53 (3217.59-4123.47) | 30.37 (25.32-35.42) | 552.13 (450.30-653.95) |
| 2030 | Both | 560.75 (466.44-655.06) | 3687.24 (3150.31-4224.17) | 30.01 (24.14-35.87) | 545.63 (427.12-664.13) |

ASR age-standardized rate

Figure S1 The changes in the proportion of cases among total burden related to hepatitis B patients aged ≥65 years to the overall age of patients from 1990 to 2021

DALYs disability-adjusted life-years


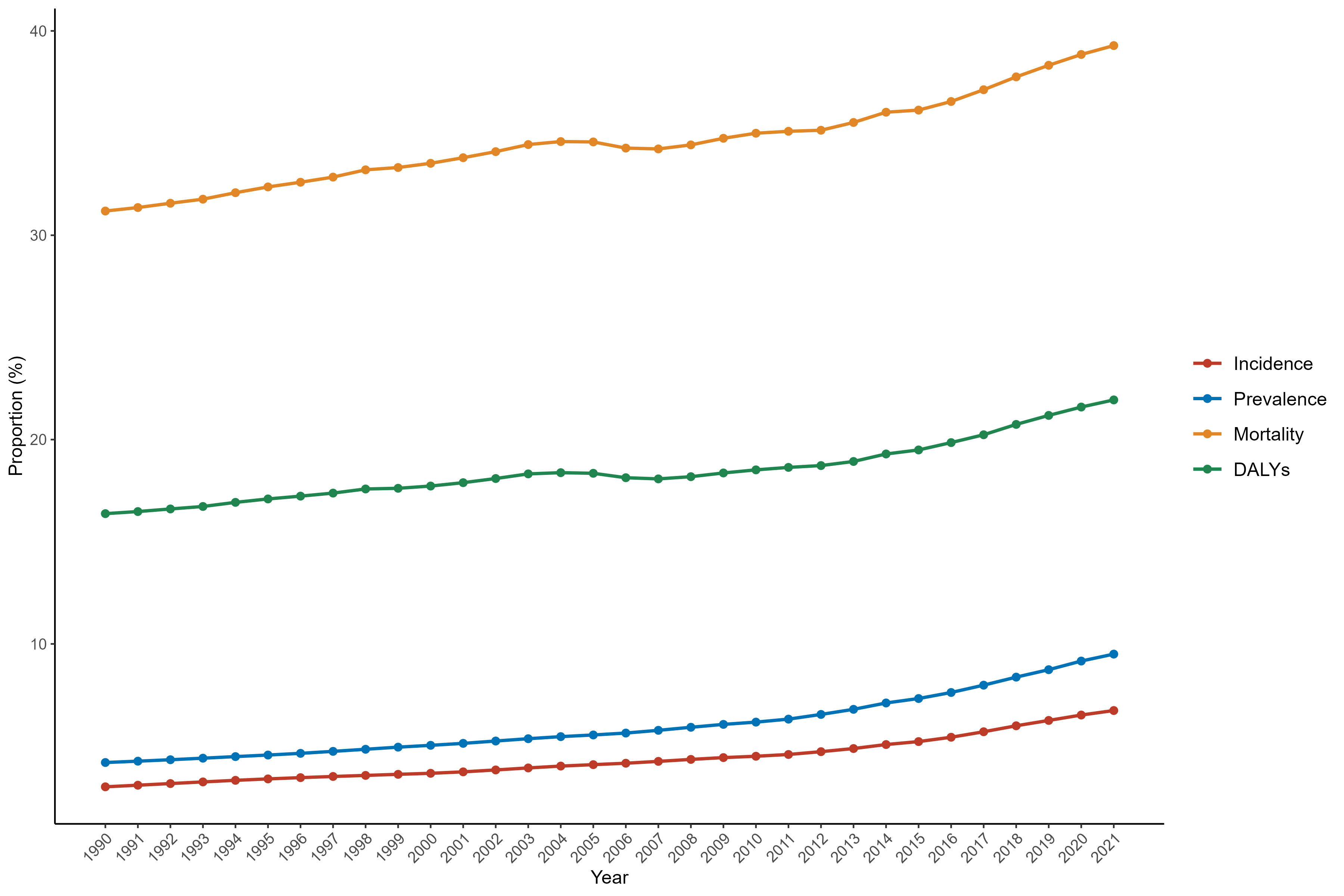


Figure S2 Temporal trend of age-standardized incidence, prevalence, mortality, and DALYs for total burden related to hepatitis B patients aged ≥65 years to the overall age of patients from 1990 to 2021

AAPC average annual percentage change, DALYs disability-adjusted life-years


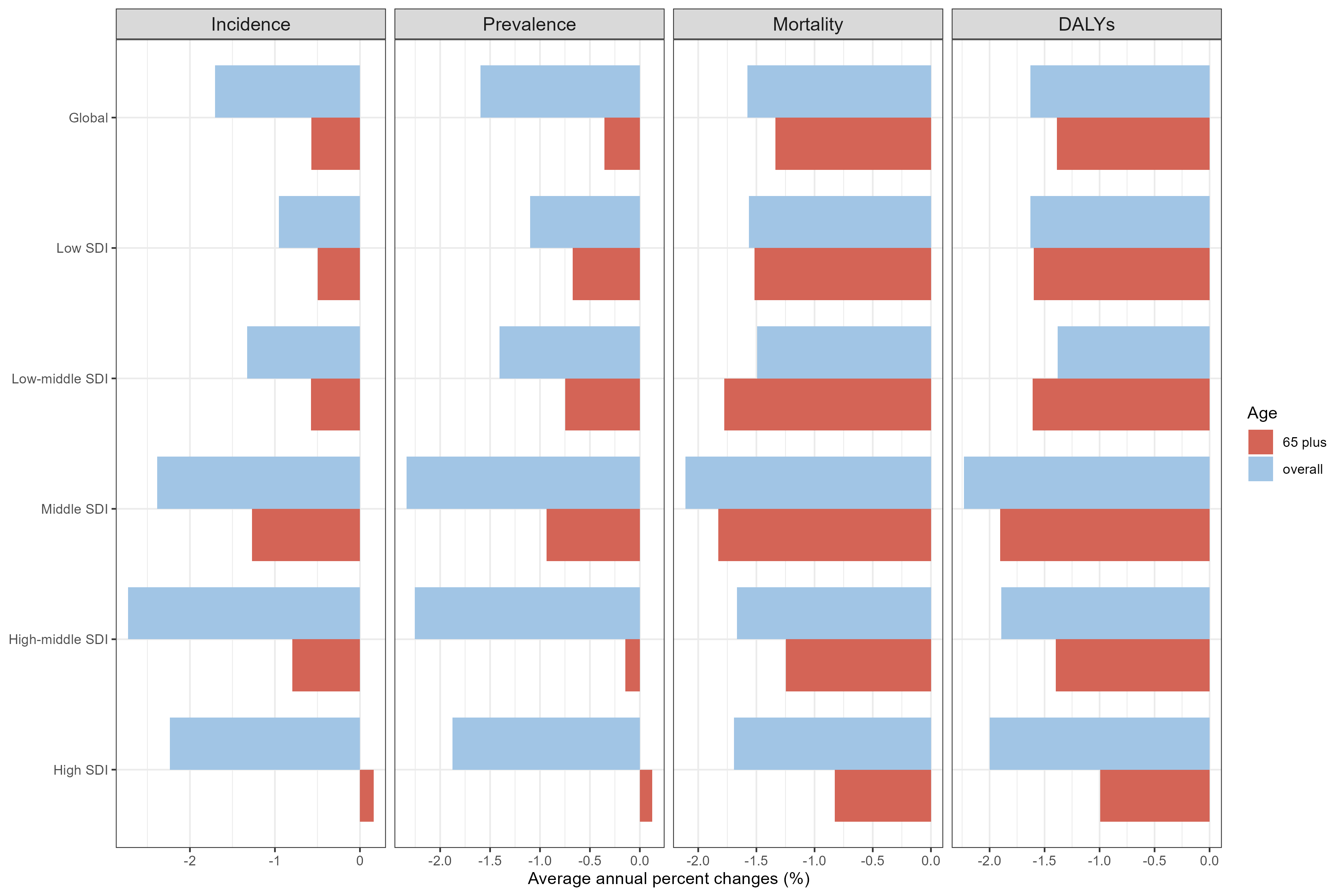


Figure S3 Temporal trend of age-standardized incidence, prevalence, mortality, and DALYs of total burden related to hepatitis B in adults aged ≥65 years from 1990 to 2021 at the global and SDI levels by sex

DALYs disability-adjusted life-years, SDI socio-demographic index


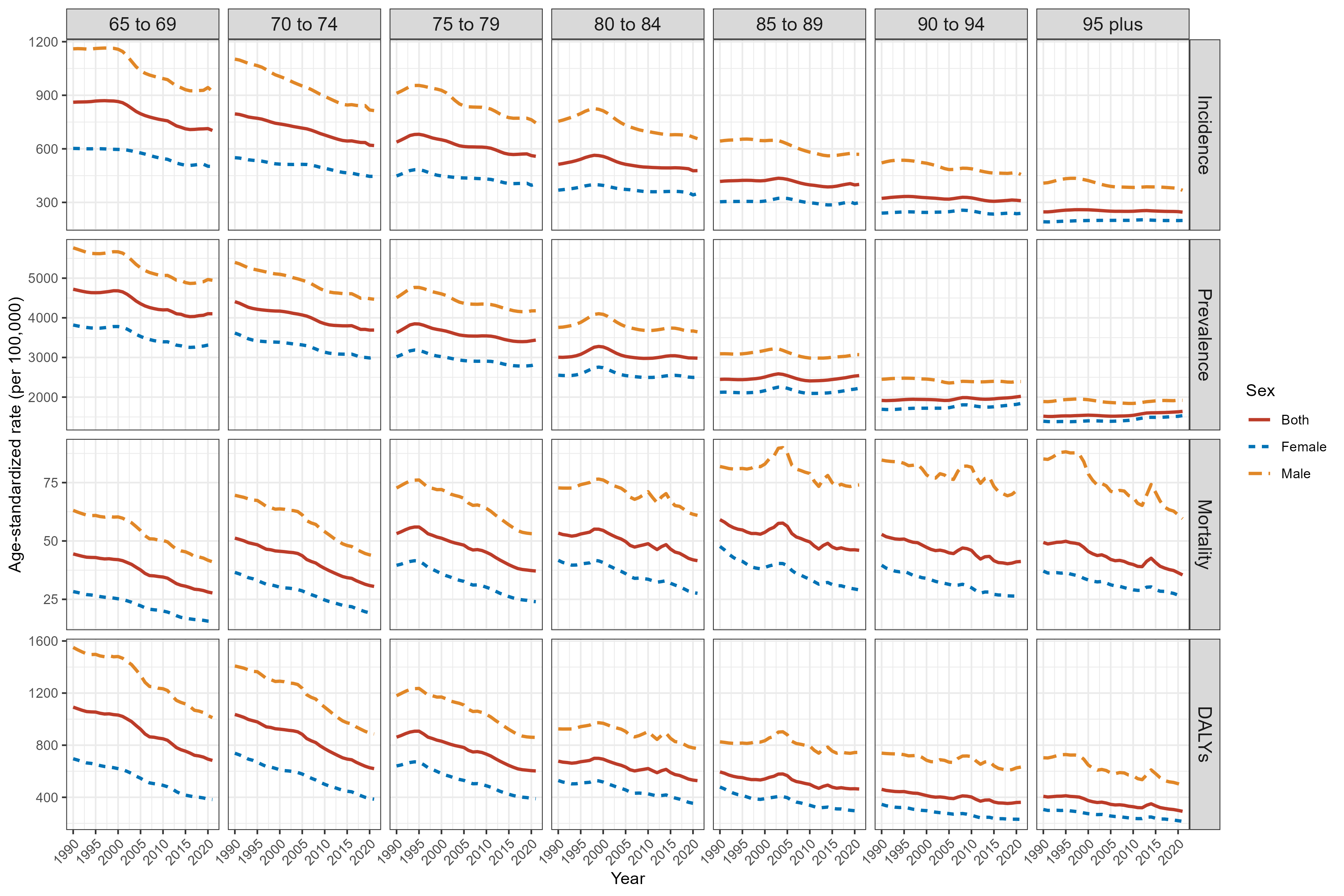


Figure S4 Temporal trend of age-standardized incidence, prevalence, mortality, and DALYs of total burden related to hepatitis B in children and adolescents from 1990 to 2021 at the global and SDI levels by sex

DALYs disability-adjusted life-years, SDI socio-demographic index


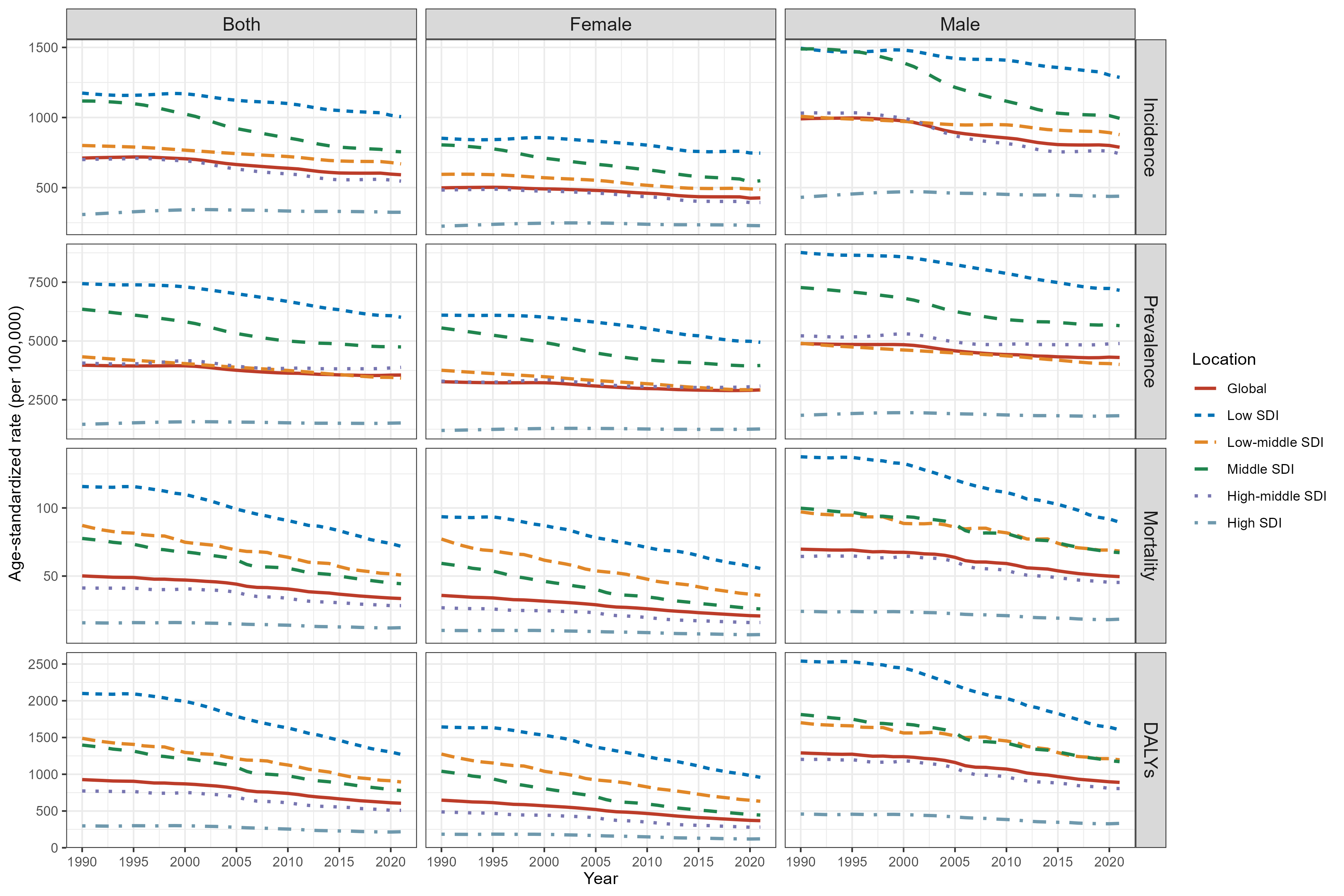


Figure S5 The AAPCs of age-standardized incidence, prevalence, mortality, and DALYs of total burden related to hepatitis B in adults aged ≥65 years from 1990 to 2021 at the global and SDI levels by sex

AAPC average annual percentage change, DALYs disability-adjusted life-years, SDI socio-demographic index


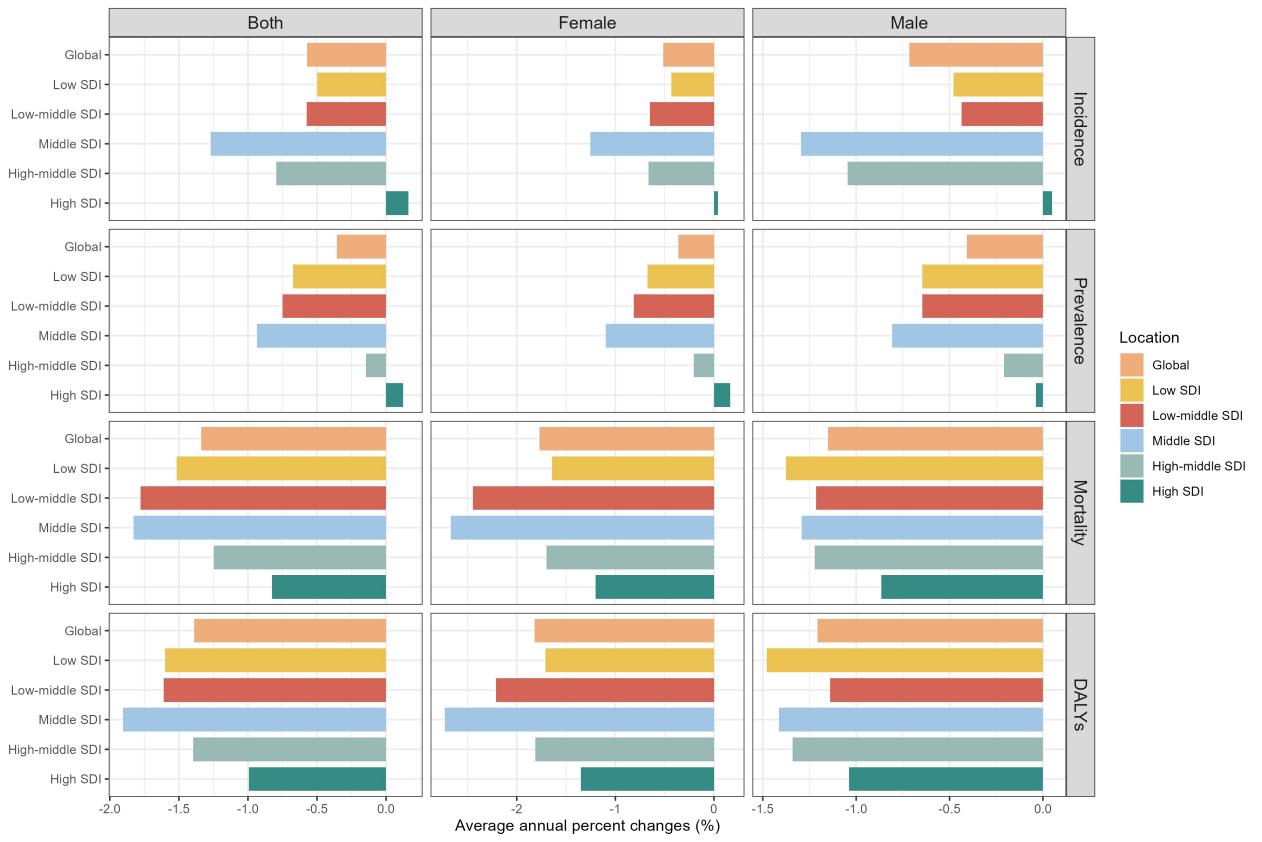


Figure S6 The AAPCs of age-standardized incidence, prevalence, mortality, and DALYs of total burden related to hepatitis B in adults aged ≥65 years from 1990 to 2021 by sex and age

AAPC average annual percentage change, DALYs disability-adjusted life-years


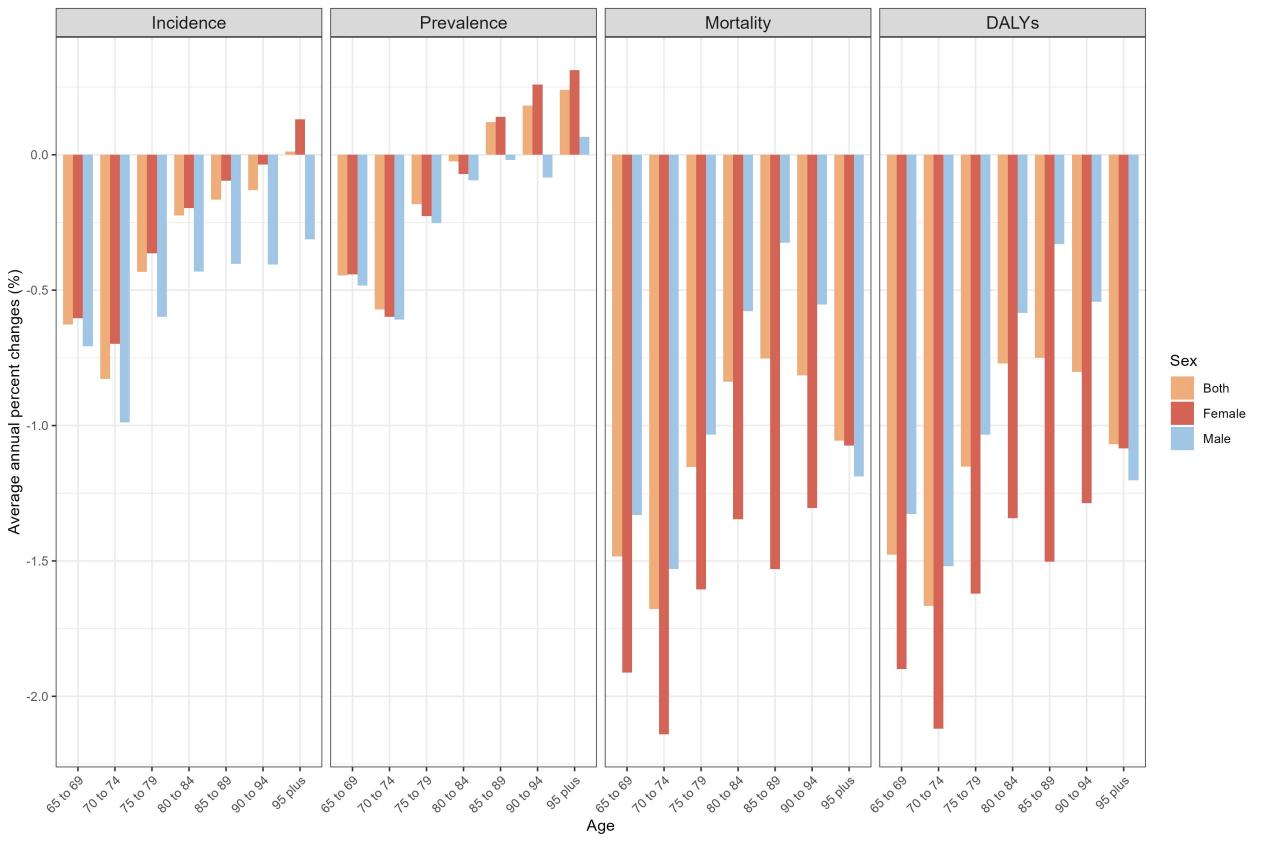


Figure S7 Prevalence and mortality of total burden related to hepatitis B in adults aged ≥65 years from 1990 to 2021 at the global level

(A) Cases and age-standardized prevalence by sex; (B) Cases and age-standardized mortality by sex; (C) Cases and age standardized prevalence by age; (D) Cases and age-standardized mortality by age.


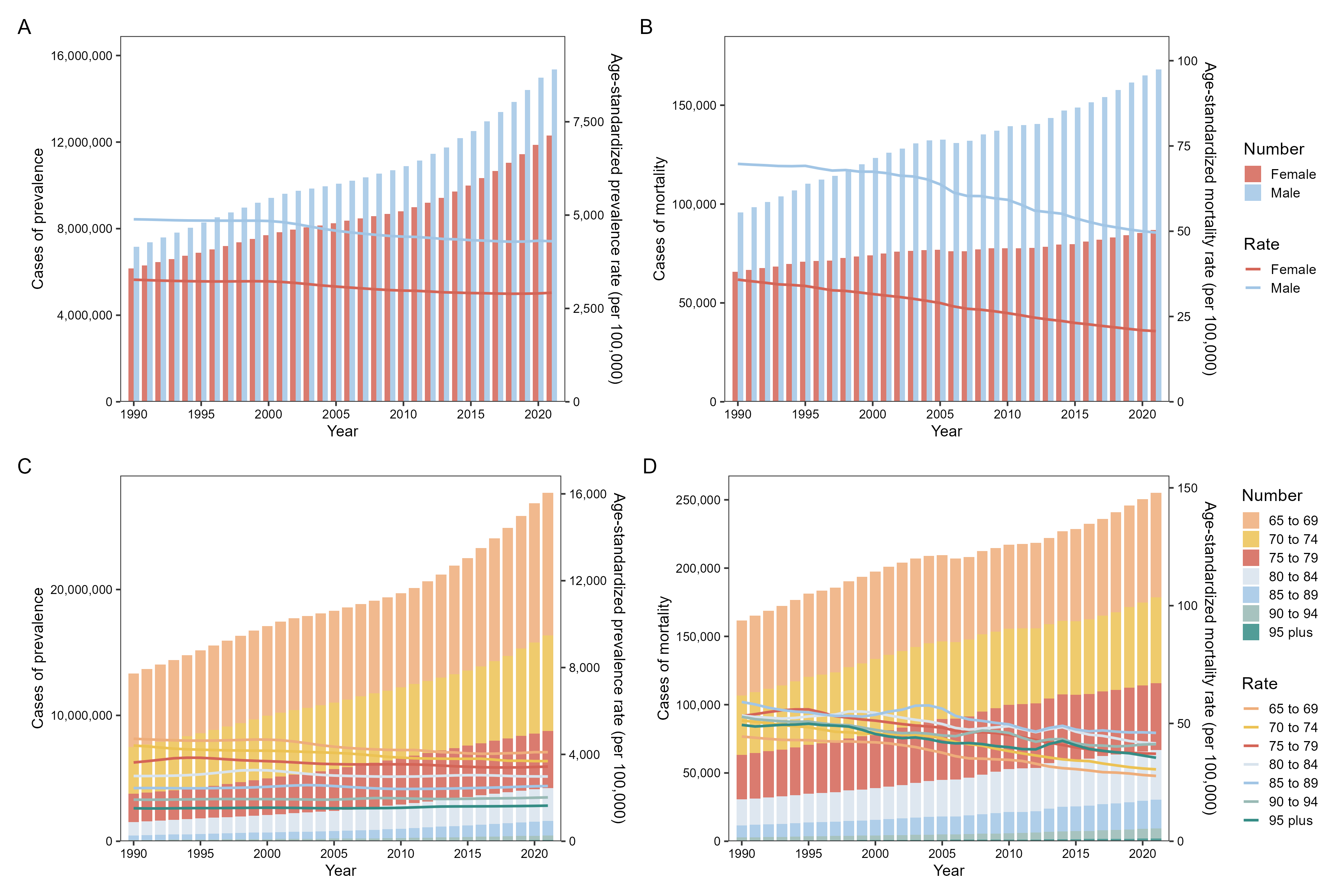


Figure S8 Temporal trend of age-standardized incidence, prevalence, mortality, and DALYs of total burden related to hepatitis B aged ≥65 years and overall age of patients from 1990 to 2021 at the global and SDI levels

DALYs disability-adjusted life-years, SDI socio-demographic index


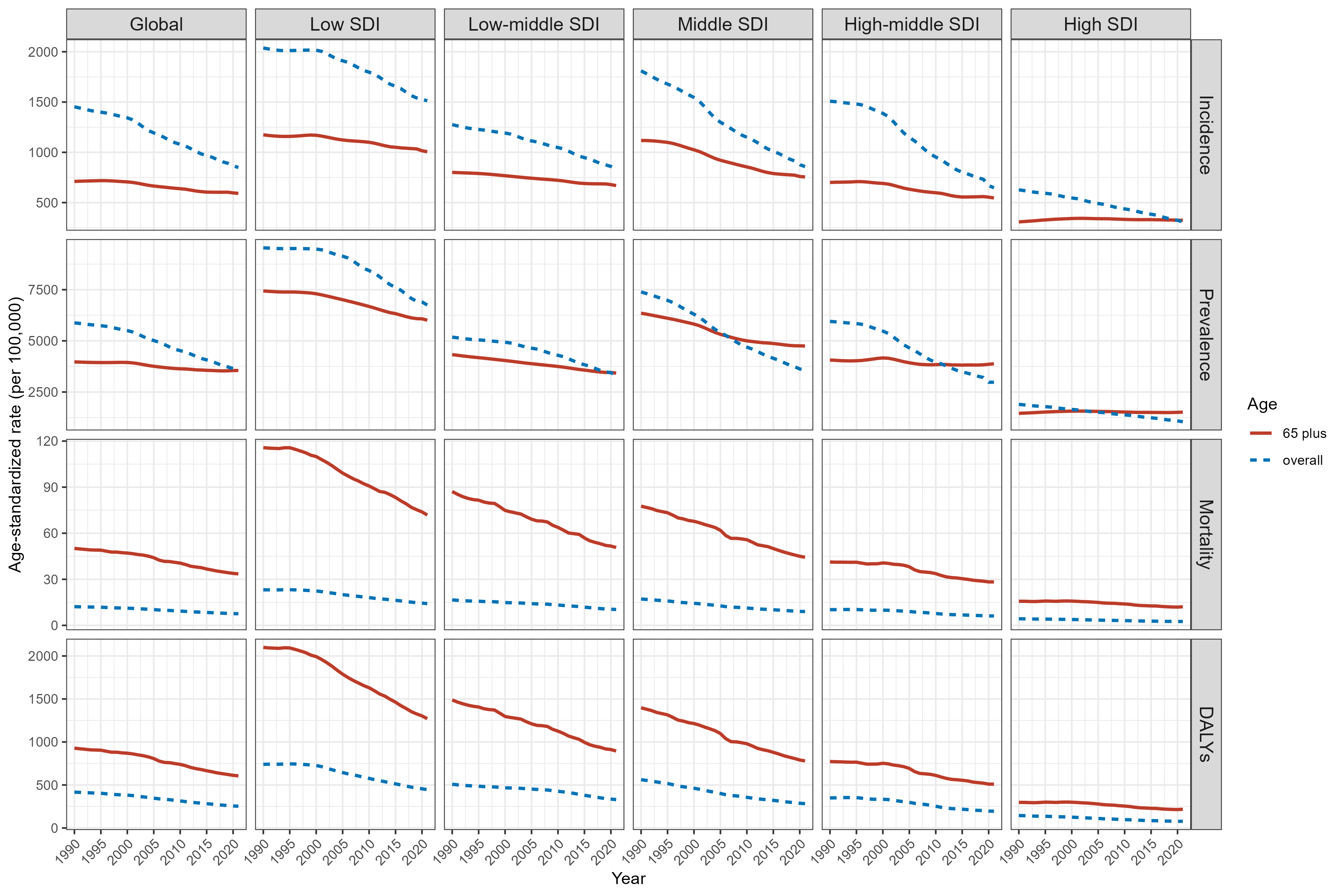


Figure S9 The AAPCs of age-standardized incidence, prevalence, mortality, and DALYs of total burden related to hepatitis B in adults aged ≥65 years from 1990 to 2021 at the regional level by sex

AAPC average annual percentage change, DALYs disability-adjusted life-years


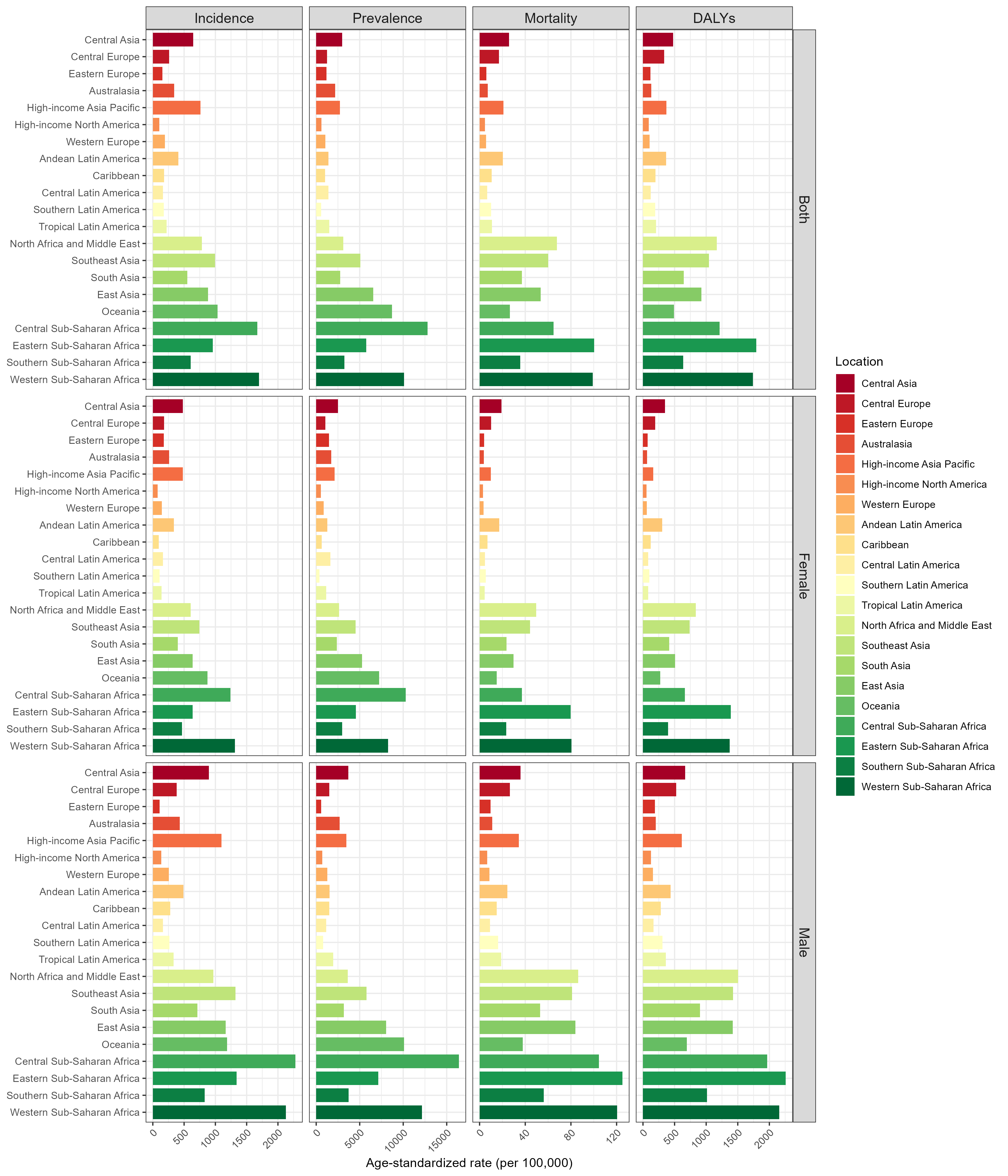


Figure S10 The cases of change of total burden related to hepatitis B in adults aged ≥65 years between 1990 and 2021 in 204 countries and territories

(A) Incidence; (B) Prevalence; (C) Mortality; (D) DALYs. DALYs disability-adjusted life-years


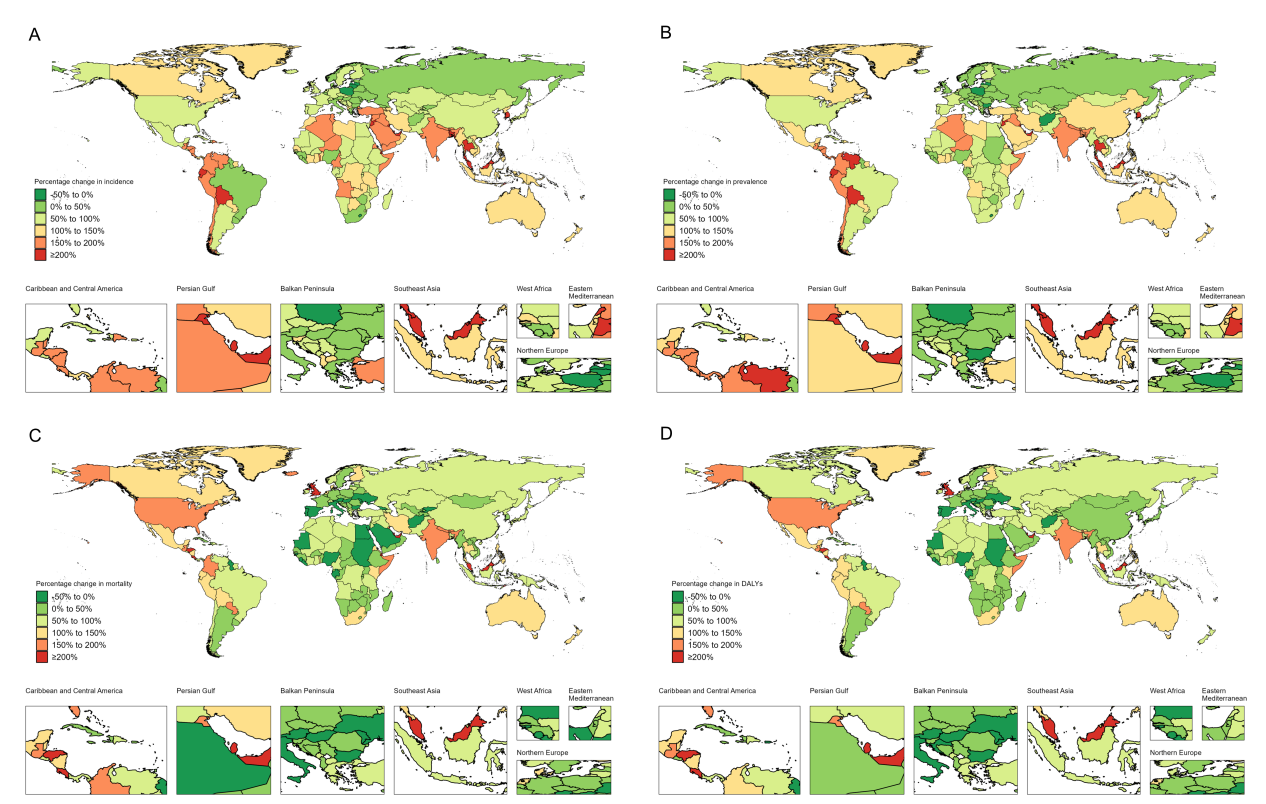


Figure S11 Age-standardized prevalence and mortality of total burden related to hepatitis B in adults aged ≥65 years in 204 countries and territories

(A) Incidence; (B) Prevalence; (C) Mortality; (D) DALYs. DALYs disability-adjusted life-years


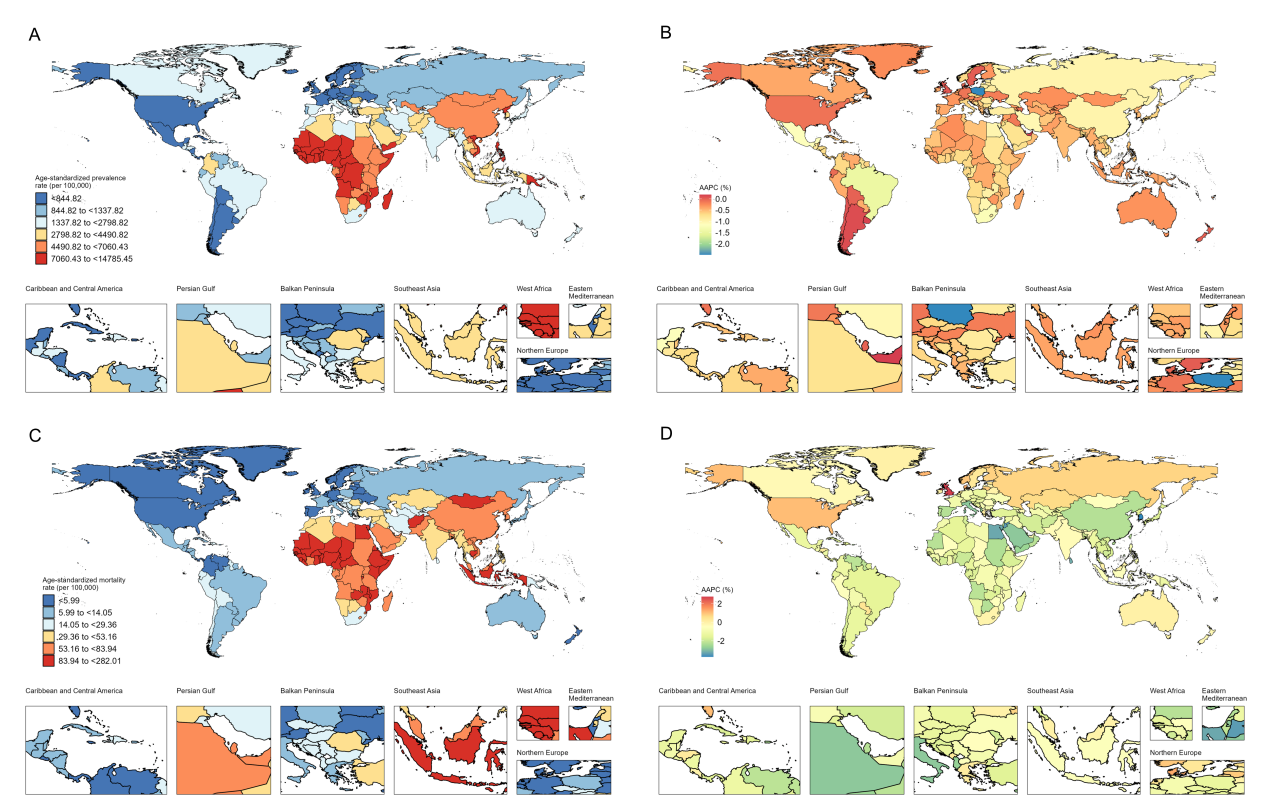


Figure S12 Global, regional, and national levels of total burden related to hepatitis B in adults aged ≥65 years by SDI

(A) Age-standardized incidence from 1990 to 2021 at the global and 21 regions levels; (B) Age-standardized incidence in 2021 in 204 countries and territories; (C) Age-standardized DALYs from 1990 to 2021 at the global and 21 regions levels; (D) Age-standardized DALYs in 2021 in 204 countries and territories. For each region, points from left to right depict estimates from each year from 1990 to 2021. Expected trends based on SDI and disease age-standardized rates in all locations were shown as the black line with LOWESS (locally weighted scatterplot smoothing) methods. DALYs disability-adjusted life-years, SDI socio-demographic index


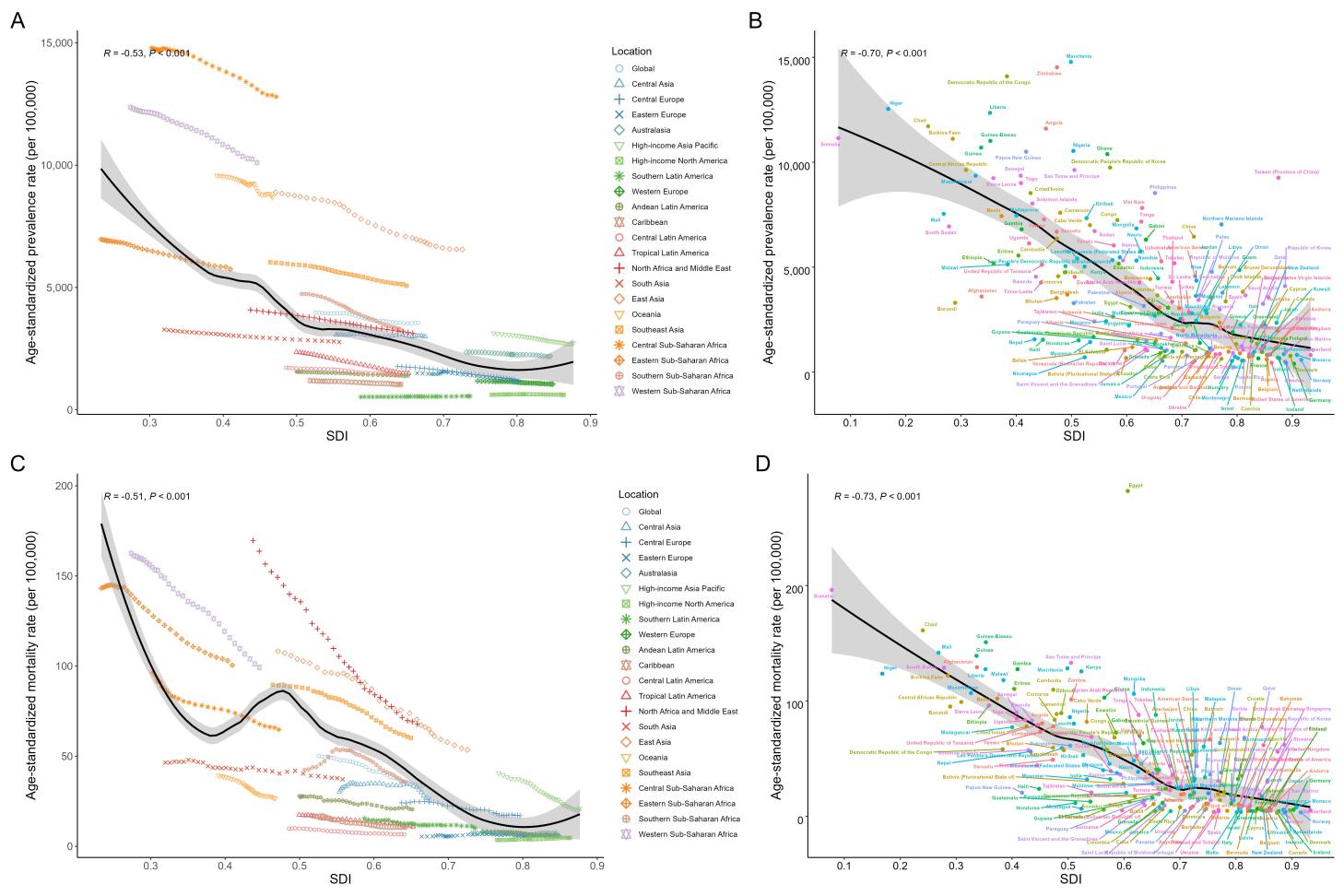

Supplement: Supplementary file 1 [file Supplementary_file_1.docx]
